# Examination of Acetylated Monosaccharides as Metabolic Probes in Bacteria

**DOI:** 10.1021/acsinfecdis.5c00765

**Published:** 2025-10-13

**Authors:** Sophia E. Nigrovic, Ankita Paul, Soumyakanta Maji, Antara Ghosh, Jack Tran, Phuong Luong, William J. Rackear, Elizabeth A. Stemmler, Karen D. Moulton, Suvarn S. Kulkarni, Danielle H. Dube

**Affiliations:** † Department of Chemistry & Biochemistry, 2050 Bowdoin College, 6600 College Station, Brunswick, Maine 04011, United States; ‡ Department of Chemistry, 29491 Indian Institute of Technology Bombay, Powai, Mumbai 400-076, India

**Keywords:** glycan, azide, metabolic labeling, bioorthogonal chemistry, esterase

## Abstract

Bacterial glycans are validated antibiotic targets due to their crucial roles in supporting bacterial fitness and survival. The array of exclusively bacterial monosaccharides and their variable expression across bacterial species and serotypes present challenges in studying these structurally diverse molecules. Probes based on bacterial sugars have emerged as useful tools in metabolic labeling studies. Prior to the metabolic processing of probes by bacteria, most metabolic probes must be transported across the bacterial cell envelope. Probe acetylation has been used as one strategy to ease passive diffusion across the lipophilic cell membrane and relies on deacetylation by esterases within cells before subsequent metabolic processing into glycans is possible. However, inefficient probe deacetylation has the potential to yield artifactual labeling rather than physiological glycan labeling. Here, we systematically explored probe acetylation as a design criterion for metabolic labeling experiments in four bacterial species. *Plesiomonas shigelloides*, *Vibrio vulnificus*, and *Helicobacter pylori* exhibited a strong preference for metabolic incorporation of acetylated probes relative to unprotected probes, whereas *Bacteroides fragilis* incorporated both unprotected and acetylated probes at comparable levels. Curiously, only *B. fragilis* had sufficient esterase activity to quantitatively deacetylate a peracetylated monosaccharide probe in situ. These findings suggest the importance of validating acetylated probes on a case-by-case basis to ensure physiologically relevant bacterial glycan labeling.

## Graphical abstract

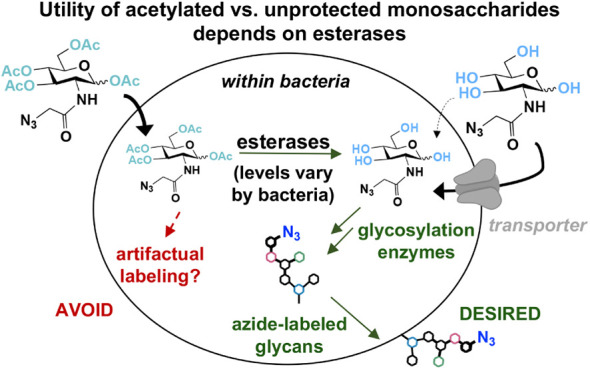

Bacteria coat themselves with a dense array of cell envelope glycans that mediate interactions with other cells and maintain a barrier from the environment, attributes that enhance bacterial fitness and promote survival. As a testament to their functional importance, bacterial glycans have served as targets of blockbuster antibiotics^1–4^ and highly effective vaccines.^5–7^ Taking stock of which glycan structures are present on which bacterial cells reveals fundamental insights into the role of these structures, as well as aids in antibiotic and vaccine development. Unfortunately, the systematic study and perturbation of bacterial glycans remain challenging. These challenges stem from bacteria utilizing >700 monosaccharides to create higher-order glycans, coupled with glycan compositional and structural variability across bacterial strains and serotypes.^8–10^

Chemical tools have made important inroads toward understanding and probing bacterial glycans.^11^ For example, metabolic glycan labeling (MGL) with monosaccharide-based reporters has facilitated the detection, discovery,^12–14^ tracking,^15^ and inhibition^16^ of bacterial glycans and their biosynthesis. In a typical MGL experiment, bacterial cells are supplemented with unnatural azide-bearing monosaccharides. Optimally, bacteria take up azidosugars, process them via endogenous carbohydrate biosynthetic pathways, and install them in place of natural monosaccharides to yield, presumably, azide-labeled glycans. Alkyne-based reporters then react via click chemistry to facilitate detection of labeled species.^17,18^ Metabolic reporters based on ubiquitous monosaccharides and a small sampling of exclusively bacterial deoxy amino sugars have been developed and offer an approach to address existing unknowns, reveal potential targets of antibiotics and vaccines, and refine our knowledge of the bacterial glycan repertoire. An important caveat, however, is that the presumed fate of monosaccharide probes into target glycans is not the only possible explanation for the labeling. Only monosaccharide reporters that are processed into bacterial glycans through carbohydrate biosynthesis enzymes have the potential to yield physiologically relevant information about bacterial glycans.

Except for metabolic reporters based on late-stage lipid-linked intermediates that are processed by glycosylation enzymes outside the cells,^19^ most metabolic reporters must be transported across the bacterial cell envelope prior to metabolic processing. One established strategy to navigate probe transport across the bacterial cell envelope is to tap into endogenous monosaccharide transport proteins for active and efficient import into cells; free sugar probes based on the bacterial monosaccharides pseudaminic acid, legionaminic acid, and muramic acid efficiently utilize transporters.^20–22^ Another strategy is to genetically introduce monosaccharide transporters to facilitate the uptake of free sugar probes when endogenous transporters are absent. A third approach is to esterify free hydroxyls on chemical reporters with acetyl moieties to facilitate passive diffusion of acetylated probes across the lipophilic cell membrane.^12,23^ Once inside cells, esterases are assumed to cleave transient acetyl-protecting groups from chemical reporters to yield unprotected, hydroxylated sugar analogs, which are then recognized and processed by carbohydrate biosynthesis enzymes (Figure 1A).^24^

**Figure 1:**
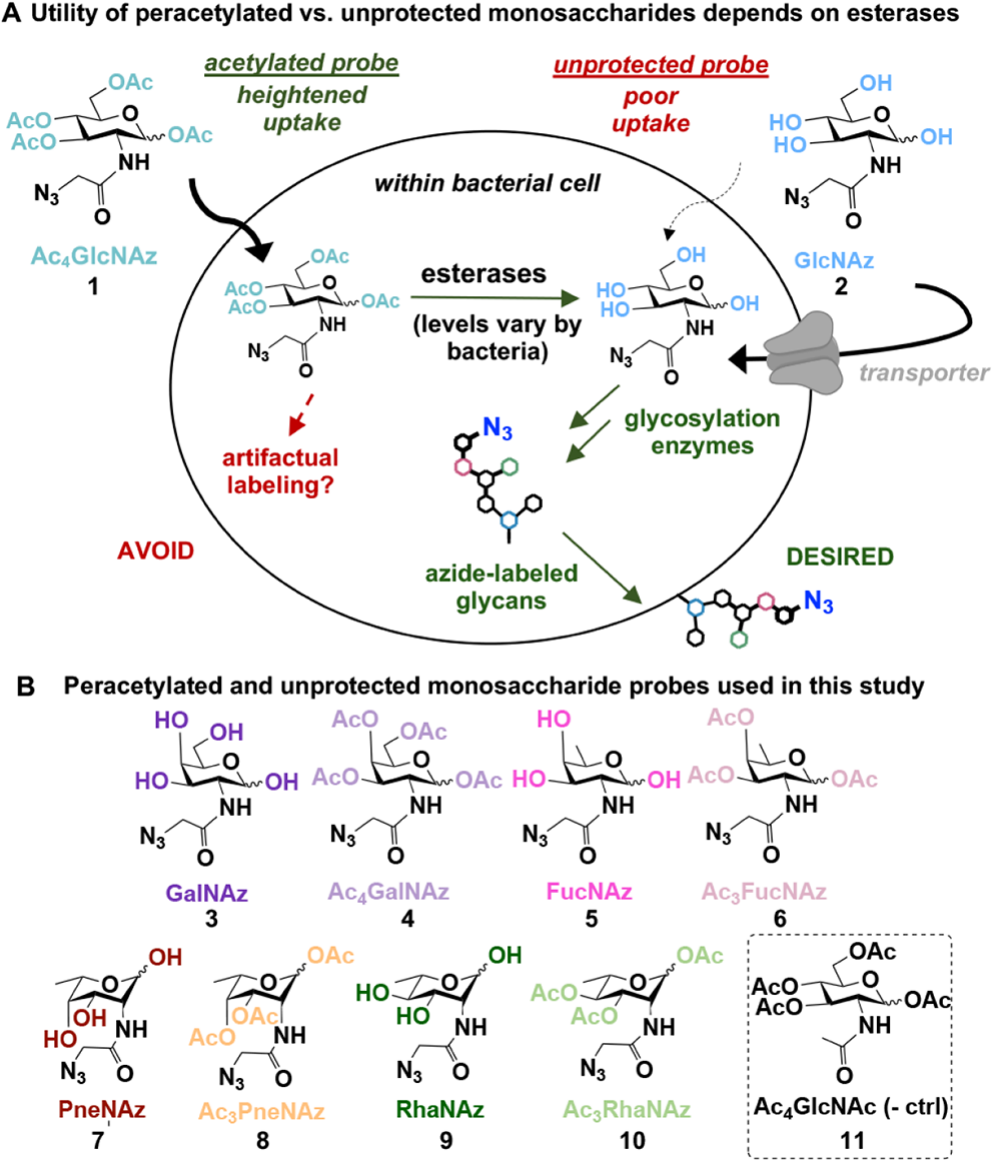
Acetylated and unprotected azidosugars were directly compared in bacterial metabolic glycan labeling experiments. (A) Unless a dedicated monosaccharide transporter is present or introduced, unprotected sugars have poor uptake across the bacterial cell envelope. Peracetylated monosaccharides facilitate entry into cells and are converted to their unprotected counterparts if sufficient levels of esterases are present. Only monosaccharides bearing hydroxyl groups are processed by glycosylation enzymes. Without sufficient esterase activity, acetylated probes may cause unwanted S-glyco-modification, leading to artifactual labeling. (B) Probes used in this study.

Despite the widespread use of metabolic reporters to study bacterial glycans, key knowledge gaps remain regarding optimal parameters for probe design. Though acetylated monosaccharides are synthetically tractable targets and have been successful as metabolic reporters in a variety of bacteria,^25^ sufficient esterase activity appears to be a necessary prerequisite for accessing cellular glycosylation pathways with acetylated monosaccharides.^26^ Indeed, peracetylated chemical reporters that are not efficiently deacetylated may not be utilized^26^ or may introduce complications in interpreting data from MGL experiments, as they can induce artifactual S-glyco-modification of cysteine residues.^27,28^ Chemical S-glyco-modification could be a significant challenge in some bacteria, like *Escherichia coli*,^29^ that lack significant esterase activity (Figure 1A). Thus, assessing which bacterial strains possess the requisite esterase activity to efficiently cleave acetyl protecting groups and yield free monosaccharide probes in situ is critical for selecting the appropriate chemical probes and interpreting the results of bacterial MGL experiments.

Here, we directly compared the utilization of unprotected and protected azidosugars across a variety of bacteria. We analyzed the usage of established free and peracetylated *N*-azidoacetyl analogues of common monosaccharides *N*-acetylglucosamine (GlcNAz **1**, **2**) and *N*-acetylgalactosamine (GalNAz **3**, **4**).^17^ We also synthesized novel unprotected *N*-azidoacetyl analogues of exclusively bacterial deoxy amino sugars d-fucosamine (FucNAz **5**), l-pneumosamine (PneNAz **7**), and l-rhamnosamine (RhaNAz **9**) for comparison with our recently developed peracetylated *N*-azidoacetyl analogues of these bacterial d- and l-sugars (Ac_3_FucNAz **6**, Ac_3_PneNAz **8**, Ac_3_RhaNAz **10**).^30,31^ All four bacterial species tested exhibited significant metabolic labeling following treatment with peracetylated probes, and only *Bacteroides fragilis* underwent comparable metabolic labeling upon treatment with unprotected azidosugars as with peracetylated probes. For the bacteria screened, the extent of labeling was highly dependent on the monosaccharide scaffold used. To assess the level of esterase activity produced by each bacterial strain, we used an established fluorescein diacetate (FDA)-based assay.^32^ Significant aryl esterase activity was detected in bacterial cells and bacterial lysates, with select bacterial species exhibiting esterase activity that was orders of magnitude greater than that of others. Consequently, to more specifically assess the ability of bacterial esterases to deprotect acetylated monosaccharide probes, we developed a high-performance liquid chromatography fluorescence detection (HPLC-FD)-based deacetylation assay using the peracetylated fluorescent monosaccharide probe *N*-acetylfucosamine-*O*-nitrobenzoxadiazole (NBD-FucNAc **12**). This assay revealed that only two of the four bacterial lysates screened exhibited sufficient esterase activity to fully deacetylate NBD-FucNAc. Thus, peracetylation of metabolic labeling probes may not be optimal as a general design criterion and should be used on a case-by-case basis.

## Results and Discussion

To evaluate peracetylation as a design criterion for metabolic labeling probes, we synthesized a panel of free and peracetylated *N*-azidoacetyl analogues of monosaccharides (**1**–**10**). These studies required us to develop routes to access unprotected versions of previously studied acetylated MGL probes based on the rare amino deoxy sugars d-fucosamine, l-pneumosamine, and l-rhamnosamine (Figure 1B). Briefly, the established peracetylated probes Ac_3_FucNAz **6**, Ac_3_PneNAz **8,** and Ac_3_RhaNAz **10** were synthesized as anomeric thiophenyl (SPh) or *p*-methoxy phenyl (PMP) glycosides according to our published procedures.^30,31^ Cleavage of anomeric SPh or PMP was achieved using *N*-bromosuccinimide (NBS) or ceric ammonium nitrate (CAN), respectively, and subsequently deacetylated using Zemplén conditions to yield FucNAz **5**, PneNAz **7,** and RhaNAz **9** (Figure 1B). Details of the synthesis procedures and characterization of novel probes are described in Figure S1.

### Preferential Metabolic Labeling with Peracetylated Monosaccharide Probes Was Observed in Select Bacteria

To directly compare the relative utilization of unprotected and protected azidosugar probes, we turned to *Plesiomonas shigelloides*, *Vibrio vulnificus*, *Helicobacter pylori*, and *B. fragilis*, bacteria that have been successfully used in metabolic labeling experiments.^15,30,33^ These bacteria were cultured in media supplemented with 1 mM of either the indicated free or peracetylated azidosugars of exclusively bacterial monosaccharides (FucNAz **5**, Ac_3_FucNAz **6**, PneNAz **7**, Ac_3_PneNAz **8**, RhaNAz **9**, Ac_3_RhaNAz **10**, Figure 1B) or common monosaccharides (GlcNAz **2**, Ac_4_GlcNAz **1**, GalNAz **3**, Ac_4_GalNAz **4**, Figure 1B), according to our published methods.^31^ The azide-free sugar peracetylated *N*-acetylglucosamine (Ac_4_GlcNAc **11**) was included as a negative control, and established azidosugar probes (Ac_4_GlcNAz **1**, Ac_4_GalNAz **4**) served as positive controls due to previous reports of their metabolic incorporation in the bacteria screened.

Based on the optimal growth parameters for each bacterial strain, cells were harvested following 1–3 days of metabolic labeling and probed for the presence of cell-surface azides using bioorthogonal chemistry. Azides on live cell surfaces were probed using strain-promoted azide–alkyne cycloaddition (SPAAC) with fluorescent Alexa Fluor 488-dibenzocyclooctyne (AF488-DBCO) and detected via flow cytometry.^15,30^ Mean fluorescence intensities of cell populations from these experiments revealed that, across all bacteria, treatment with at least one peracetylated azidosugar led to a substantial enhancement of azide-dependent signal, resulting in a significant increase in fluorescence intensity relative to cells treated with the azide-free control sugar Ac_4_GlcNAc **11** (Figure 2; Figure S2). More specifically, *P. shigelloides* and *V. vulnificus* exhibited a significant enhancement in fluorescence following treatment with all five of the peracetylated monosaccharide probes (Ac_4_GlcNAz **1**, Ac_4_GalNAz **4**, Ac_3_FucNAz **6**, Ac_3_PneNAz **8**, Ac_3_RhaNAz **10**) but not after treatment with the corresponding unprotected monosaccharide probes (GlcNAz **2**, GalNAz **3**, FucNAz **5**, PneNAz **7**, RhaNAz **9**), suggesting that probe acetylation is a crucial parameter for probe uptake and incorporation in these two bacteria. *H. pylori* exhibited robust levels of cell-surface azides following treatment with acetylated monosaccharides Ac_4_GlcNAz **1**, Ac_4_GalNAz **4**, and Ac_3_FucNAz **6** and, to a lesser extent, with Ac_3_PneNAz **8** (Figure 2C). By contrast, *H. pylori* incorporated only one free sugar, GlcNAz **2**, to an appreciable extent and, notably, at substantially lower levels than observed following treatment with its acetylated counterpart Ac_4_GlcNAz **1** (Figure 2C). Therefore, *H. pylori* also appears to have a preference for acetylated probes. Strikingly, *B. fragilis* was the only bacterium that utilized free sugars as efficiently as peracetylated probes. In particular, treatment of *B. fragilis* with the unprotected probes GalNAz **3** and FucNAz **5** led to fluorescence levels that were on par with those observed upon treatment with the corresponding peracetylated sugars, Ac_4_GalNAz **4** and Ac_3_FucNAz **6**, respectively (Figure 2D; Figure S2D). An analysis of cell-surface MGL via flow cytometry histograms reinforces a general preference for acetylated probes relative to their unprotected counterparts across *P. shigelloides*, *V. vulnificus*, and *H. pylori* in a head-to-head comparison, with *B. fragilis* as a notable exception (Figure S2).

**Figure 2:**
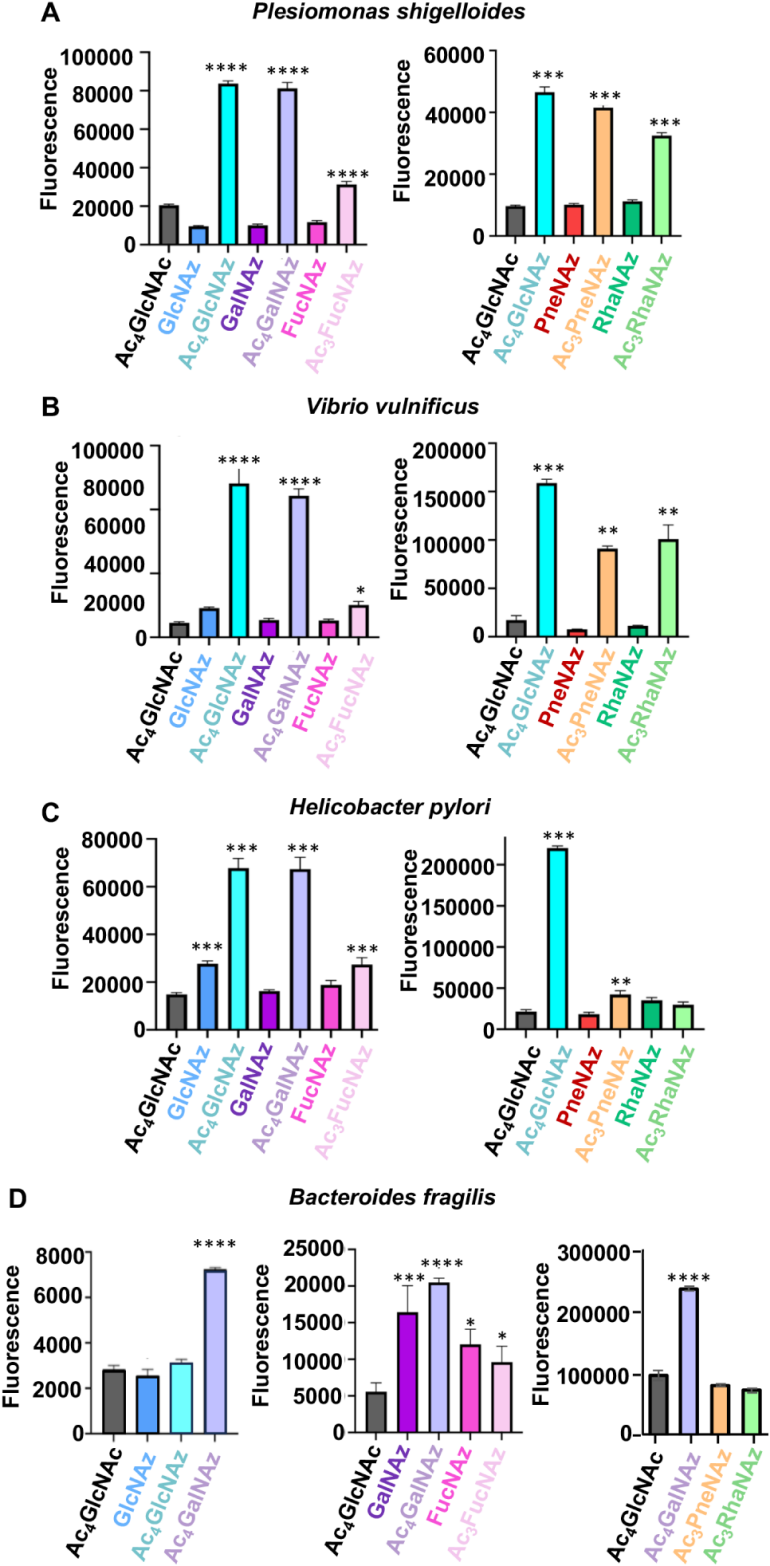
Metabolic labeling of bacterial cells treated with peracetylated versus unprotected d- and l-azidosugars was assessed via a flow cytometry assay following the reaction of azide-labeled cells with AF488-DBCO. Fluorescence of treated (A) *Plesiomonas shigelloides*, (B) *Vibrio vulnificus*, (C) *Helicobacter pylori*, and (D) *Bacteroides fragilis* was detected by flow cytometry and is shown plotted as mean fluorescence intensity for triplicate samples. Each plot is based on a separate experiment. Data are representative of replicate experiments. Statistical significance relative to Ac_4_GlcNAc treatment was determined by a one-way ANOVA test. * *p* < 0.05; ** *p* < 0.01; *** *p* < 0.001; **** *p* < 0.0001.

The extent of metabolic labeling was highly dependent on the peracetylated monosaccharide scaffold used. For example, though all four of these bacteria appeared to process more than one acetylated azidosugar probe onto their cell surfaces, these probes led to different relative magnitudes of cell surface azides, with some leading to a higher azide-dependent signal than others. Presuming treatment with monosaccharide probes yields physiologically relevant labeling of bacterial glycans, this observation may reflect the relative abundance of each monosaccharide in surface glycans of these bacteria. The extent to which different carbohydrate-activating enzymes and glycosyltransferases required for glycan biosynthesis tolerate *N*-azidoacetyl-bearing substrates in place of native *N*-acetyl sugars may also have an effect. Alternatively, differences in labeling could be due to more complicated metabolic pathways or nonglycosylation incorporation of azides.

### Bacterial Cells Exhibit Robust Aryl Esterase Activity

Metabolic labeling experiments revealed the impact of acetyl protecting groups on probe uptake and incorporation in select bacterial cells. These results suggest the absence of endogenous transporters that efficiently import unprotected monosaccharides into *P. shigelloides*, *V. vulnificus*, and *H. pylori*. By contrast, *B. fragilis* appears to efficiently transport unprotected monosaccharides GalNAz **3** and FucNAz **5** into its cells. We were curious whether the bacteria used in this study contain the requisite esterase activity to remove acetyl protecting groups within cells, unveiling unprotected probes. Probe deacetylation is crucial, as only unprotected sugars will be recognized as substrates by glycosylation enzymes, leading to enzymatic incorporation into newly assembled glycans. Incomplete probe deacetylation caused low-level artifactual chemical S-glyco-modification in mammalian cells following treatment with peracetylated probes, including Ac_4_GlcNAz **1** and Ac_4_GalNAz **4**.^27,28^ Thus, sufficient esterase activity in bacterial cells is necessary to ensure metabolic labeling reports on physiological rather than artifactual glycosylation in bacteria.

Therefore, we next measured whether the esterase activity within the bacterial strains studied correlated with the corresponding efficiency of peracetylated azidosugar utilization. Biochemical evidence of general esterase activity within *P. shigelloides*, *V. vulnificus*, *H. pylori*, and *B. fragilis* cells was measured using a well-established fluorescein diacetate (FDA) assay.^32^ Flow cytometry analysis revealed that bacterial treatment with FDA led to a concentration-dependent increase in the fluorescence intensity of treated cells relative to untreated cells (Figure 3A), consistent with activation of the fluorogenic probe by endogenous aryl esterases within cells to yield fluorescein. *V. vulnificus* and *B. fragilis* exhibited robust FDA activation that compared favorably to activation exhibited by mammalian THP-1 monocytes (Figure S3A) known to have esterases,^34^ suggesting relatively high esterase activity with this probe. In contrast, *P. shigelloides* and *H. pylori* exhibited lower relative whole cell fluorescence following incubation with FDA. Since the cell envelope could pose an impediment to FDA uptake and concomitant esterase activation, we also measured FDA activation by esterases in bacterial lysates. Assessment of esterase activity in bacterial lysates following incubation with 0.6 mM FDA revealed substantial fluorescence relative to that of untreated controls (Figure 3B), corresponding to significant esterase-dependent hydrolysis. Mimicking fluorescence seen in whole cells, *B. fragilis* and *V. vulnificus* lysates appeared to have very high levels of FDA activation that were comparable to those observed with THP-1 lysates (Figure S3B). *P. shigelloides* and *H. pylori* lysates, in contrast, exhibited more modest but still significant FDA activation.

**Figure 3:**
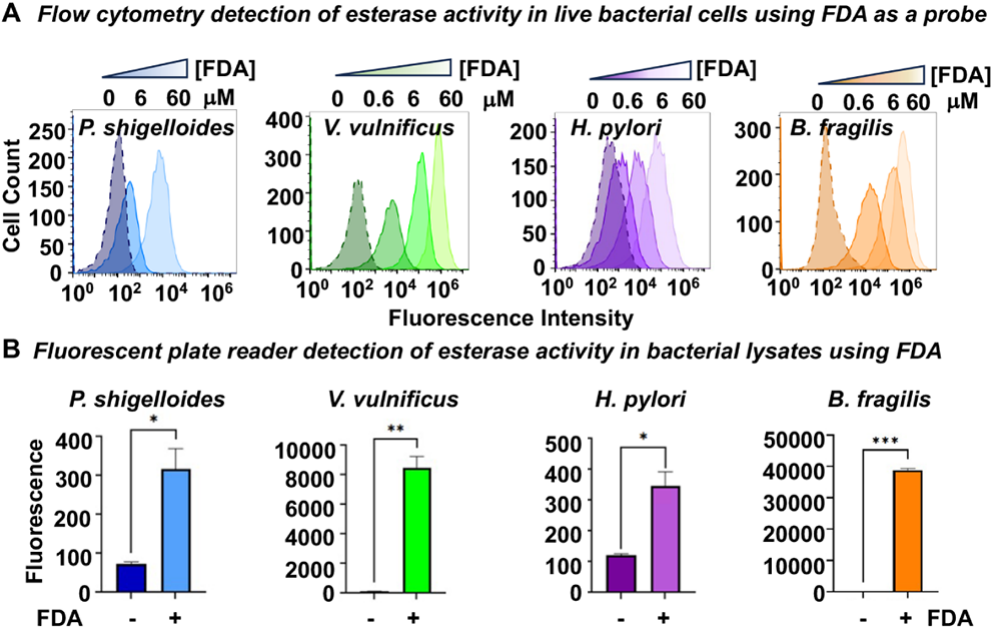
Assessment of esterase activity in (A) bacterial cells and (B) bacterial lysates using fluorescence activation of fluorescein diacetate (FDA) as a readout. (A) Bacterial cells were treated with 0–60 μM of FDA as indicated, and fluorescence was measured by flow cytometry of treated cells following 15 min of incubation. (B) Bacterial lysates were treated without FDA (−) or with 0.6 μM FDA (+) μM for 15 min, and fluorescence was measured by a fluorescence plate reader following 15 min of incubation. Data are representative of replicate experiments. Statistical significance was determined by a one-way ANOVA test. * *p* < 0.05; ** *p* < 0.01; *** *p* < 0.001.

### Select Bacteria Have Sufficient Esterase Activity to Fully Deacetylate a Peracetylated Monosaccharide Probe

Given the more labile nature of aryl esters found in FDA compared to acyl esters present in acetylated monosaccharides,^35^ coupled with the high likelihood of cellular esterases discriminating across substrate classes, we sought more direct evidence of the requisite esterase activity in bacterial cells to deacetylate monosaccharide probes. Toward this end, we reasoned that a peracetylated monosaccharide probe based on one of the monosaccharide scaffolds used in our metabolic labeling experiments (Figure 2) would afford a means to assess and compare monosaccharide deacetylation in bacteria and allow correlations of relative acyl esterase activity with the extent of metabolic labeling. In particular, we were drawn to the bacterial monosaccharide d-FucNAc as a scaffold since the corresponding azidosugar Ac_3_FucNAz **6** was incorporated at low to modest levels in the bacteria investigated and at lower levels than other acetylated sugars, leading us to wonder about its deacetylation within bacteria. We designed the peracetylated constitutively fluorescent monosaccharide probe FucNAc-*O*-nitrobenzoxadiazole (NBD-FucNAc **12**; Figure 4A) due to the attractive optical properties of the NBD moiety (λ_ex_ = 463 nm, λ_em_ = 536 nm), which would facilitate its use in an HPLC fluorescence detection-based deacetylation assay. Details of the synthesis procedures and characterization of NBD-FucNAc **12** are described in Figure S4. Fluorescence HPLC analysis of the synthetic standard NBD-FucNAc **12** revealed peaks at 23.3 and 24.6 min that correspond to the fully acetylated NBD-FucNAc **12** probe as a mixture of α- and β-*glycosides* (Figure 4A; Figure S5).

**Figure 4:**
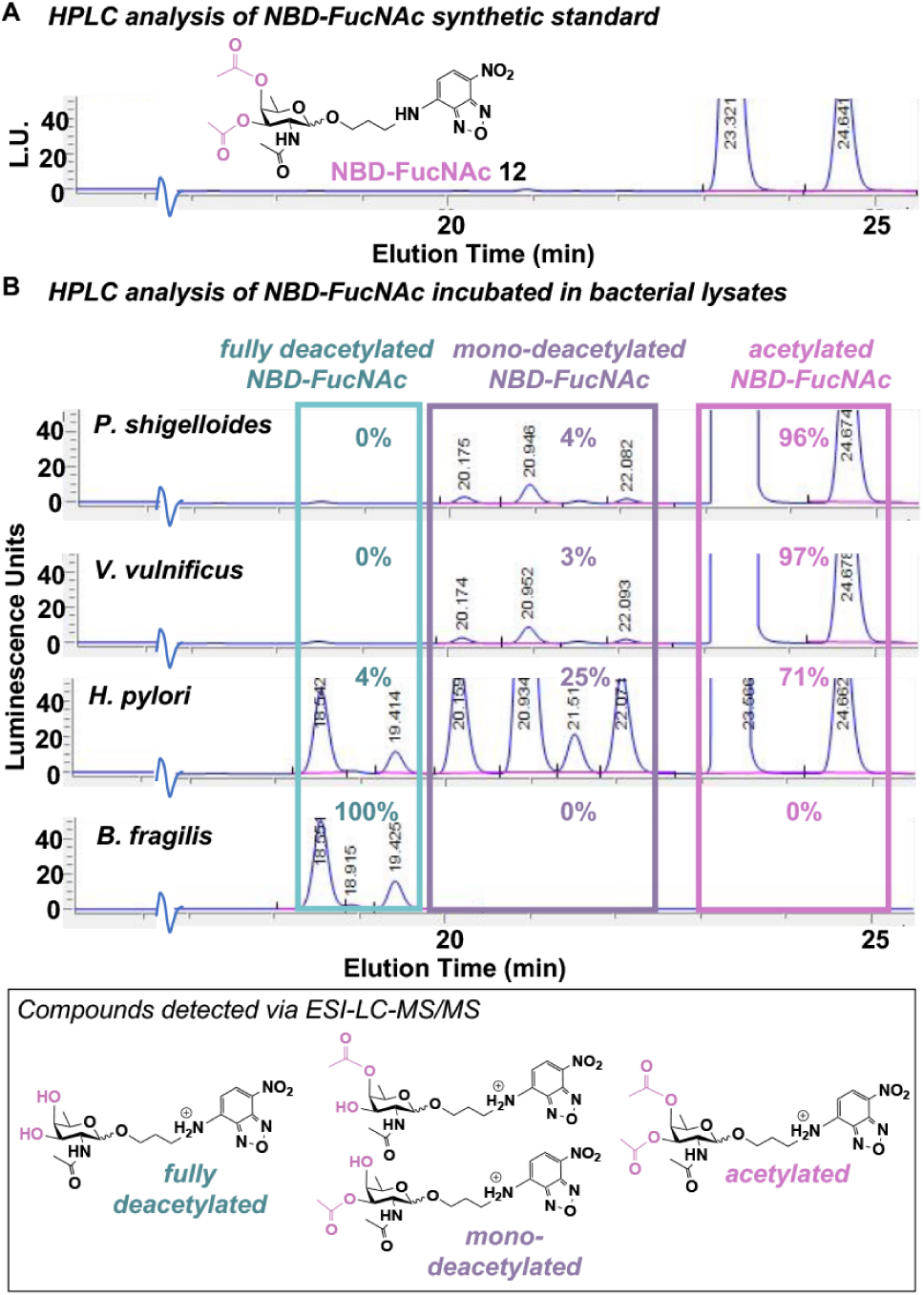
Assay to measure deacetylation of a monosaccharide-based probe. (A) HPLC analysis of NBD-FucNAc **12** synthetic standard revealed acetylated α- and β-glycosides. (B) HPLC analysis of NBD-FucNAc **12** incubated in the indicated bacterial lysates showed peaks corresponding to the acetylated, monodeacetylated, and fully deacetylated NBD-FucNAc, as confirmed by ESI-LC-MS/MS detection (Figure S5). Integration values of HPLC peaks were used to calculate the percent of acetylated, monodeacetylated, and fully deacetylated NBD-FucNAc detected in bacterial lysates following incubation with NBD-FucNAc **12** and are shown as percentage values above the peaks in each sample.

To assess whether bacterial esterases recognized this acetylated monosaccharide as a substrate, we incubated bacterial lysates with NBD-FucNAc **12** and then monitored samples for the acetylated and/or deacetylated probe. ESI-LC-MS/MS analyses of *H. pylori* samples incubated with acetylated NBD-FucNAc **12** confirmed the presence of the acetylated, monodeacetylated, and fully deacetylated probe in *H. pylori* samples (Figure 4; Figure S5), indicating the presence of esterase activity in this bacterium and facilitating peak assignment in fluorescence HPLC chromatograms. Fluorescence HPLC analysis of *H. pylori* samples revealed peaks corresponding to acetylated NBD-FucNAc **12**, as well as the appearance of new peaks with elution times in the 20–22 min range that correspond to monodeacetylated NBD-FucNAc (a mixture of α- and β-*glycosides* with 3-OH or 4-OH) and at 18.5 and 19.4 min that correspond to fully deacetylated NBD-FucNAc (Figure 4B). An integration of these peaks in *H. pylori* samples showed that 71% of the area under the curves was accounted for by remaining acetylated probe **12**, 25% was due to the monoacetylated NBD-FucNAc, and 4% of the probe had been fully deacetylated in *H. pylori* samples. By contrast, >95% of the acetylated NBD-FucNAc **12** remained in *P. shigelloides* and *V. vulnificus* samples, with <5% converted to the monodeacetylated form. Strikingly, *B. fragilis* samples contained neither the acetylated NBD-FucNAc **12** nor the monodeacetylated NBD-FucNAc. Instead, *B. fragilis* samples contained only the fully deacetylated NBD-FucNAc, signifying high levels of carbohydrate-active esterases that led to the quantitative conversion of peracetylated NBD-FucNAc **12** to its fully deacetylated counterpart (Figure 4B). Taken together, these data indicate that *B. fragilis* appears to have efficient esterase activity with the acetylated substrate **12**, *H. pylori* has modest carbohydrate esterase activity with this substrate, and *P. shigelloides* and *V. vulnificus* exhibited inefficient deacetylation of probe **12**. Only *B. fragilis* and *H. pylori* appear to have sufficient esterase activity to completely deacetylate NBD-FucNAc **12** at all.

There was no clear correlation between each bacterium’s ability to deacetylate NBD-FucNAc **12** and the extent of metabolic labeling observed when that bacterium was treated with Ac_3_FucNAz **6** or, more generally, with other peracetylated azidosugar probes. Though *B. fragilis* and *H. pylori* most effectively deacetylated NBD-FucNAc **12** (Figure 4B), these two bacteria did not exhibit remarkably higher metabolic labeling with Ac_3_FucNAz **6** or with the other peracetylated probes (Figure 2). Analogously, *P. shigelloides* and *V. vulnificus* were robustly metabolically labeled with at least one acetylated azidosugar, yet they showed minimal esterase activity with acetylated NBD-FucNAc **12** in these studies. However, there does appear to be a correlation between levels of carbohydrate esterase activity observed (Figure 4B) and each bacterium’s relative ability to metabolically incorporate unprotected versus peracetylated probes. Strikingly, *B. fragilis’* robust carbohydrate esterase activity (Figure 4B) is correlated to its near-comparable utilization of unprotected and peracetylated probes (Figure 2D). Similarly, *H. pylori’s* modest level of carbohydrate esterase activity is correlated to this bacterium’s ability to metabolically incorporate the unprotected sugar GlcNAz **2**, albeit at lower levels than Ac_4_GlcNAz **1** (Figure 2C). By contrast, *P. shigelloides’* and *V. vulnificus’* near-absent carbohydrate esterase activities (Figure 4B) are correlated to these bacteria not utilizing unprotected probes (Figure 2A, B). Taken together, these data suggest that carbohydrate esterase activity may correlate to the efficiency of metabolic incorporation of free versus peracetylated sugars. These parameters may provide useful information about the likelihood of acetylated probes yielding physiologically relevant bacterial glycan labeling.

## Discussion

Given the importance of glycans in supporting bacterial fitness, mediating bacteria–host interactions, and, in some instances, driving bacterial pathogenesis, expanding the repertoire of tools to study and understand bacterial glycans is critical.^25^ Metabolic glycan labeling has been an enabling approach to probe glycan epitope expression on bacteria, yield insights into bacterial glycan biosynthesis genes, and indicate potential targets for novel glycan-based diagnostic and interference strategies. Despite the widespread use of metabolic reporters to study bacterial glycans, key knowledge gaps remain regarding optimal parameters for probe design. To the best of our knowledge, this is the first comprehensive head-to-head comparison of acetylated versus unprotected monosaccharide probes across a range of bacteria. This direct comparison queries the value of transient acetylation of monosaccharide probes to ease passive uptake into bacteria versus using unprotected monosaccharide probes that rely on transporters for efficient import into bacteria. Here, we sought to address the value of acetylated versus unprotected monosaccharides as metabolic probes in three bacterial pathogens, *P. shigelloides*, *V. vulnificus*, and *H. pylori*, as well as in the ubiquitous commensal bacteria *B. fragilis*. Toward this end, we produced a series of acetylated and unprotected azidosugar analogues of common and rare bacterial monosaccharides to allow direct comparison of metabolic labeling. We then paired metabolic labeling studies with an exploration of bacterial esterase activity using an established aryl ester probe and a novel acetylated fluorescent sugar-based probe.

This study revealed that peracetylated monosaccharides led to significantly higher levels of cell surface azides than unprotected sugar probes in *P. shigelloides*, *V. vulnificus*, and *H. pylori* (Figure 2). This finding suggests that peracetylation may be an attractive feature for monosaccharide probes and indicates relatively inefficient metabolic labeling with free sugar probes in these bacteria. The extent of metabolic labeling observed was dependent on the bacterial species screened and the choice of the peracetylated monosaccharide scaffold. For example, Ac_3_PneNAz **8** was robustly incorporated into *P. shigelloides* and *V. vulnificus* but not by *H. pylori* (Figure 2). By contrast, Ac_4_GlcNAz **1** was efficiently incorporated onto *P. shigelloides*, *V. vulnificus*, and *H. pylori* cells. If the observed metabolic labeling is due to physiological glycosylation events, differences in the extent of probe incorporation may reflect relative levels of glycan epitope expression in these bacteria, the relative abundance of that particular monosaccharide scaffold in each bacterium’s glycans, or the relative substrate promiscuity or restrictiveness of active sites in different carbohydrate-processing enzymes. Regardless, the clear trend in the three pathogenic bacterial species tested was more cell labeling with peracetylated probes relative to their unprotected counterparts. A notable exception to this trend was observed with the commensal bacterium *B. fragilis*, which used free and peracetylated analogues of GalNAz and FucNAz to comparable extents (Figure 2D).

Recent reports on the potential downsides of using peracetylated probes that are not efficiently deacetylated by cellular esterases^27,28^ prompted us to explore esterase levels in *P. shigelloides*, *V. vulnificus*, *H. pylori*, and *B. fragilis*. Moderate to high levels of esterase activity were detected across all four of these bacterial species in an FDA-based assay (Figure 3), initially leading us to conclude that there was likely sufficient esterase activity for efficient deacetylation of peracetylated monosaccharide probes. However, follow-up experiments with NBD-FucNAc **12** in a novel fluorescence HPLC assay revealed relatively low levels of deacetylation for this monosaccharide-based probe in all bacteria screened except *B. fragilis* (Figure 4). *P. shigelloides* and *V. vulnificus* showed minimal carbohydrate esterase activity with the substrate acetylated NBD-FucNAc **12** (Figure 4), despite robust labeling observed when these bacteria were treated with peracetylated azidosugars (Figure 2). Only *B. fragilis* and *H. pylori* were able to fully deacetylate NBD-FucNAc **12**, and only *B. fragilis* did so quantitatively. Confounding interpretation of these results is the likely possibility that esterases have substrate specificity, with a preference for some carbohydrate scaffolds and protecting groups over others. In a comparison of *N*-acetyl-muramic acid probes for metabolic labeling of bacterial peptidoglycan, Grimes and coworkers found that acetate protecting groups on the hydroxyl groups are not tolerated by bacterial esterases.^26^ By contrast, they found that esterases efficiently remove a methyl ester protecting group from the carboxylic acid of the *N*-acetylmuramic acid probes. These findings speak to the importance of exploring esterase activity within each bacterial species analyzed if probe acetylation is used as a design parameter. Prudent tool developers and users should characterize the metabolic fate of protected probes in every new organism being studied to ensure an appropriate level of specificity and labeling of the target glycan.

The experiments conducted in this paper do not probe the extent to which target glycan labeling versus artifactual glycosylation, if any, is occurring. While it is established in mammalian systems that peracetylated sugars can lead to artifactual chemical S-glyco-modification, the extent to which this may be happening in bacteria is unknown. The cell lysate assay developed by Chen and coworkers offers an excellent qualitative approach to examine the extent of background reactivity observed with acetylated relative to unprotected monosaccharides in bacteria.^36^ Scoring the extent of labeling in bacteria where the corresponding glycosylation pathway has been genetically removed offers another approach to probe the potential background reactivity of acetylated probes. Mass spectrometry approaches developed by Woo and coworkers are well positioned to quantify relative amounts of physiological versus artifactual S-glyco-modification events in bacteria.^37^ Our findings suggest that metabolic labeling with peracetylated probes in some bacteria may not necessarily report only on physiologically relevant glycosylation.^27,28^ However, even with the possibility of undesired chemical background labeling, O-acetylated chemical reporters can still be a generally reliable tool^37^ and have shown utility for studying glycans and their biosynthesis genes in *B. fragilis* ^15^ and *H. pylori*.^38^ Additional investigation is needed to confirm that peracetylated sugar probes are reporting on true glycosylation events in *P. shigelloides* and *V. vulnificus*.

The original impetus for this study was to compare the efficacy of acetylated monosaccharide probes to unprotected analogues for metabolic glycan labeling experiments. The limited utility of free azidosugars as metabolic substrates in these experiments in *P. shigelloides*, *V. vulnificus*, and *H. pylori* is likely due to inefficient transport of free sugars and points to the value of acetylated monosaccharide probes if they are appropriately vetted in the target bacteria in which they are used. Successes in mammalian cells with monosaccharide probe modifications that ease uptake into cells, are readily cleaved in situ, and are not prone to artifactual glycosylation events are a source of inspiration for alternative probe design parameters to study bacterial glycans.^39^ Reliable chemical tools will be critical for studying bacterial glycan structures, diversity, and biosynthesis, potentially setting the stage to develop glycan-based diagnostics and therapeutics.

## Conclusion

Bacterial glycans are antibiotic targets and vaccine candidates with enormous untapped potential. This work describes the relative efficiency of acetylated monosaccharide probes relative to unprotected probes for the metabolic labeling of bacterial cells. However, relatively low carbohydrate esterase activity was observed in bacterial lysates incubated with the peracetylated sugar probe NBD-FucNAc, suggesting the importance of exploring esterase activity within each bacterial species analyzed if probe acetylation is used as a design parameter. Broadly, this work provides key insights into the bacterial glycochemistry toolkit and provides guidelines for developers and users of metabolic glycan labeling tools.

## Methods

### Materials and Chemical Synthesis

Organic chemicals were purchased from MilliporeSigma. *H. pylori* strain G27 ^40^ was a gift from Manuel Amieva (Stanford University). *P. shigelloides* (ATCC 51903), *V. vulnificus* (ATCC 43382), *B. fragilis* (ATCC 23745), and AGS cells (ATCC CRL-1739) were purchased from ATCC and grown according to the supplier’s instructions. Ac_4_GlcNAc **11**, GlcNAz **2**, Ac_4_GlcNAz **1**, GalNAz **3**, Ac_4_GalNAz **4**, Ac_3_-d-FucNAz **6**, Ac_3_-l-PneNAz **8**, and Ac_3_-l-RhaNAz **10** were synthesized as previously described.^17,31,41^ *N*-azidoacetyl analogues of bacterial sugars *N*-acetyl-d-fucosamine (FucNAz **5**), *N*-acetyl-l-pneumosamine (PneNAz **7**), and *N*-acetyl-l-rhamnosamine (RhaNAz **9**), as well as peracetylated NBD-FucNAc **12**, were synthesized using standard organic chemistry procedures and characterized by standard techniques, including ^1^H and ^13^C NMR spectroscopy and mass spectrometry. Probes were purified by using flash silica gel chromatography. Fluorescein diacetate (FDA) was purchased from MilliporeSigma (St. Louis, MO). Alexa Fluor 488-dibenzocyclooctyne (AF488-DBCO) was purchased from Fisher Scientific (Waltham, MA).

### Metabolic Glycan Labeling

Bacteria were inoculated into rich liquid media to an optical density measured at 600 nm of 0.3–0.4 prior to treatment with 1 mM of Ac_4_GlcNAc **11** as an azide-free negative control to capture background fluorescence using bioorthogonal SPAAC-based assays, or with 1 mM of the azidosugar probes **1**–**10**. Ac_4_GlcNAz **1** and Ac_4_GalNAz **4** were included as positive controls due to established metabolic incorporation in the bacterial species used in that experiment.^15,27^

### Flow Cytometry Detection of Azide-Labeled Glycans on Cells

Flow cytometry was performed to measure the incorporation of azido sugars into cell surface glycans on live cells. After 1–3 days of metabolic labeling, bacteria in liquid culture were centrifuged at 2000*g* for 17 min at room temperature and washed three times in 3 mL of 1× phosphate-buffered saline (PBS) with 1% bovine serum albumin (BSA, Sigma-Aldrich, St. Louis, MO) before reaction with AF488-DBCO (5 μM) for 1 h at 37 °C in the dark. Samples were then rinsed three times in PBS with 1% BSA prior to flow cytometry analysis. Whole cell samples were analyzed by a BD Accuri C6 Plus flow cytometer (BD Biosciences, Franklin Lakes, NJ), with 10,000 live cells counted in each of three technical replicates, and the raw data were analyzed with FlowJo Software (BD Biosciences, Franklin Lakes, NJ). Each metabolic labeling experiment was repeated independently, and the figures show representative histograms.

### Bacterial Cell Lysis

Following 1–3 days of metabolic labeling, *P. shigelloides*, *V. vulnificus*, *H. pylori*, and *B. fragilis* were centrifuged at 2000*g* for 17 min at 20 °C and then washed three times in 3 mL of 1× PBS. After the third wash, the cells were resuspended in 50–75 μL of lysis buffer (20 mM Tris base, pH 7.4, 1% Igepal, 150 mM NaCl, 1 mM EDTA) with 1× protease inhibitor cocktail (MilliporeSigma) and lysed with vigorous pipetting. For all bacterial strains, cell lysate solutions were subjected to three freeze thaw cycles (alternating −80 and 70 °C for 10 min each or an ethanol and dry ice bath and 37 °C for 5 min each) prior to water bath sonication (Ultrasonic Bath 1.9 L, Thermo Fisher Scientific, Waltham, MA) for 30 min. Cell lysates were centrifuged at 17000*g* for 15 min, and the supernatant containing soluble proteins was separated. Supernatants were collected and stored at −20 °C for further analysis. Protein concentrations of clarified lysates were determined by the DC Protein Assay (Bio-Rad, Hercules, CA) and standardized to a protein concentration of 3.0 mg/mL for use in experiments to measure esterase activity.

### Mammalian Cell Growth

Human leukemia-derived THP-1 monocytes were grown to 10^6^ cells/mL in 10 mL of warm RPMI 1640 supplemented with 20% FBS at 37 °C and 5% CO_2_. THP-1 cells were used directly in esterase activity assays or were lysed in lysis buffer with 1× protease inhibitor cocktail (MilliporeSigma) for use as a positive control in esterase activity assays.

### Detection of Esterase Activity Using Fluorescein Diacetate Deacetylation

Fluorescence generated by hydrolysis of fluorescein diacetate (FDA, MilliporeSigma) by cellular esterases was used as a proxy to measure esterase activity in whole cells and cell lysates.^32^ A stock solution of FDA (6 mM in DMSO) was serially diluted to 60 μM, 6 μM, and 0.6 μM in 1× PBS. Both flow cytometry and plate reader assay protocols were validated by experiments with THP-1 cells, which are known to have high esterase activity.^34^ Bacteria from agar plates were inoculated into 3.5 mL of liquid culture to an optical density at 600 nm of 1.0. THP-1 cells were harvested at 10^6^ cells/mL. For live cell harvest, cells in liquid culture were centrifuged and rinsed with PBS containing 1% BSA, then incubated in the dark with FDA (0.6 μM, 6 μM, or 60 μM in 1× PBS with 1% BSA) for 15 min at 37 °C. Treated samples were then analyzed by flow cytometry. Whole cell samples were analyzed using a BD Accuri C6 Plus flow cytometer, with 10,000 live cells counted in each of three technical replicates. The raw data were analyzed with FlowJo Software (BD Biosciences, Franklin Lakes, NJ). Each FDA experiment was repeated independently, and the figures show representative histograms. For analysis of esterase activity, standardized cell lysates (3 mg/mL protein) were incubated with dH_2_O (0 μM FDA) or 0.6 μM FDA in dH_2_O for 15 min in a 96-well plate. Fluorescence resulting from FDA hydrolysis was detected by a GloMax Discover Microplate Reader (Promega, Madison, WI). Fluorescence of lysis buffer without bacterial lysates treated with 0 or 0.6 μM FDA was measured as a negative control.

### Detection of Esterase Activity Using NBD-FucNAc Deacetylation

Standardized bacterial cell lysates (3 mg/mL protein) were incubated overnight in lysis buffer containing 0.4 mM NBD-FucNAc **12** or no additional supplement at 37 °C. Samples were filtered through a Pierce concentrator PES filter (10K MWCO, Thermo Fisher Scientific) during a 50 min spin at 7500*g* to separate proteins from small molecules, and the flow-through was collected for further analysis. NBD-FucNAc probes were enriched from samples by chromatography on a Sep-Pak 3-cc C18 cartridge (MilliporeSigma) using a vacuum manifold. The column was wetted twice with 2 mL acetonitrile and equilibrated with four washes of 2 mL 0.1% trifluoroacetic acid (TFA) in water. The flow-through was loaded onto the column, and the column was washed four times with 2 mL 0.1% TFA in water. Finally, compounds were eluted with three applications of 600 μL elution buffer containing 50% acetonitrile and 0.1% TFA in water. Visual analysis revealed the presence of a yellow substance in the second and third elutions, which indicated fractions likely to contain NBD-FucNAc and its derivatives. Solvent was removed from these elutions *in vacuo*. Following solvent removal, the remaining material was resuspended in 5% acetonitrile:95% water for analysis by high-performance liquid chromatography (HPLC, Agilent 1100 Series HPLC System, Agilent Technologies, Santa Clara, CA). Samples were chromatographed using a reverse-phase BDS HYPERSIL C18 250 mm × 3 mm column (5 μm particle size, Thermo Fisher Scientific). An elution gradient of 0.1% H_3_PO_4_ in LCMS-grade water (solvent A) and 0.1% H_3_PO_4_ in LCMS-grade acetonitrile (solvent B, see Table S1) and a flow rate of 0.5 mL/min were used for all analyses. Material on the column was detected by an Agilent 1100 series diode-array detector (DAD) and fluorescence detector (FD) (DAD G1315B; FD G1321A, λ_ex_ 460 nm, λ_em_ 530 nm; Agilent Technologies, Santa Clara, CA). Alongside samples, a 5% acetonitrile blank and a 0.1 mM NBD-FucNAc synthetic standard were analyzed for comparative purposes and peak assignment.

## Supplementary Material



## Notes

Full experimental details, compound characterization data, and synthetic schemes to access compounds **5**, **7**, **9**, and **12**, flow cytometry histograms from metabolic labeling experiments, THP-1 whole cell and lysate data from the FDA assay, and ESI-LC–MS/MS detection of monodeacetylated and dideacetylated NBD-FucNAc **12** (PDF)

## Abbreviations

MGL: metabolic glycan labeling
GlcNAz: *N*-azidoacetylglucosamine
GalNAz: *N*-azidoacetylgalactosamine
D-FucNAz: *N*-azidoacetyl-d-fucosamine
L-PneNAz: *N*-azidoacetyl-l-pneumosamine
L-RhaNAz: *N*-azidoacetyl-l-rhamnosamine
Ac_3_FucNAz: peracetylated d-FucNAz
Ac_3_PneNAz: peracetylated l-PneNAz
FDA: fluorescein diacetate
HPLC-FD: high-performance liquid chromatography fluorescence detection
ESI-LC–MS/MS: electrospray ionization-liquid chromatography–mass spectrometry/mass spectrometry
NBD-FucNAc: peracetylated fluorescent monosaccharide probe *N*-acetylfucosamine-O-nitrobenzoxadiazole
PMP: *p*-methoxy phenyl
CAN: ceric ammonium nitrate
AF488-DBCO: Alexa Fluor 488-dibenzocyclooctyne
SPAAC: strain-promoted azide–alkyne cycloaddition
DMSO: dimethyl sulfoxide
DAD: diode-array detector
*H. pylori*: *Helicobacter pylori*
*B. fragilis*: *Bacteroides fragilis*
*P. shigelloides*: *Plesiomonas shigelloides*
*V. vulnificus*: *Vibrio vulnificus*
ATCC: American Type Culture Collection
PBS: phosphate buffered saline
NMR: nuclear magnetic resonance
HRP: horse radish peroxidase
EDTA: ethylenediaminetetraacetic acid
BSA: bovine serum albumin
TFA: trifluoroacetic acid
