# Supplementary material for: Examination of Acetylated Monosaccharides as Metabolic Probes in Bacteria
Source: ACS Infect Dis. 2025 Oct 13;11(11):3355–63. doi: 10.1021/acsinfecdis.5c00765 (PMC12624832; doi:10.1021/acsinfecdis.5c00765)
Supplement: Supplementary file 1 [file id5c00765_si_001.pdf]

Examination of acetylated monosaccharides as metabolic probes in bacteria

Sophia E. Nigrovic[1†], Ankita Paul[2†], Soumyakanta Maji[2], Antara Ghosh[2], Jack Tran[1], Phuong Luong[1], William J. Rackear[1], Elizabeth A. Stemmler[1], Karen D. Moulton[1], Suvarn S. Kulkarni[2]*, and Danielle H. Dube[1]*

[1]Department of Chemistry & Biochemistry, Bowdoin College,
6600 College Station, Brunswick, Maine 04011
[2]Department of Chemistry, Indian Institute of Technology Bombay,
Powai, Mumbai 400076, India

*Corresponding author: ddube@bowdoin.edu; suvarn@chem.iitb.ac.in
†These authors contributed equally to this work.

Supporting Information

Table of Contents

Chemistry……………………………………s-2
General……………………………………s-2
Compound **5**……………………………………s-3
Compound **7**……………………………………s-4
Compound **9**……………………………………s-5
Compound **12**……………………………………s-7

Biology……………………………………s-8
General……………………………………s-8
Bacterial strains and culture conditions……………………………………s-8
Metabolic glycan labeling……………………………………s-8
Flow cytometry detection of azide-labeled cells. ……………………………………s-9
Bacterial cell lysis……………………………………s-9
Mammalian cell growth……………………………………s-10
Detection of esterase activity using fluorescein diacetate deacetylation……………………………………s-10
Detection of esterase activity using NBD-FucNAc deacetylation……………………………………s-11
Chip-based nanoLC-MS/MS analysis of *H. pylori* treated with NBD-FucNAc……………………………………s-12

Supplemental Figures……………………………………s-14
Figure S1……………………………………s-14
Figure S2……………………………………s-15
Figure S3……………………………………s-16
Figure S4……………………………………s-17
Figure S5……………………………………s-18

Supplemental References……………………………………s-19

NMRs……………………………………s-20

Chemistry

General. All reactions were conducted under a dry nitrogen atmosphere. Solvents (CH_2Cl_2 >99%, THF 99.5%, acetonitrile 99.8%, DMF 99.5%) were purchased in capped bottles and dried under sodium or CaH_2. All other solvents and reagents were used without further purification. All glassware used were oven-dried before use. TLC was performed on precoated aluminum plates of silica gel 60 F254 (0.25 mm, E. Merck). Developed TLC plates were visualized under a shortwave UV lamp and by heating plates that were dipped in ammonium molybdate/cerium (IV) sulfate solution. Paraffin oil bath was used for heating wherever required. Silica gel column chromatography was performed using silica gel (100−200 mesh) and employed a solvent polarity correlated with TLC mobility. NMR experiments were conducted on 500 and 400 MHz instruments using $CDCl_3$ (D, 99.8%) and D_2O (D, 99.9%) as solvents. Chemical shifts are relative to the deuterated solvent peaks and are in parts per million (ppm). $^1H-^1H$ COSY and HSQC were used to confirm proton assignments. Mass spectra were acquired in ESI-TOF (time-of-flight) mode. Specific rotation experiments were measured at 589 nm (Na) and 25 °C.

Compound 14:

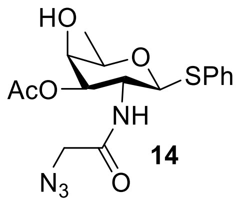

14

$Pd(OH)_2$ on carbon (0.1 g) was added to a clear solution of **13**[2] (0.2 g, 0.61 mmol) in EtOH (8 mL) and the solution was stirred at room temperature under hydrogen (1 atm) for 1 h. After complete consumption of starting material the reaction mixture was filtered through celite pad and washed with EtOH. The filtrate was concentrated and the residue was used for the next step without any purification. The residue obtained after solvents removal was dissolved in CH_3CN (4 mL), to this $NaHCO_3$ (0.11 mg, 1.85 mmol) and azido acetic acid (0.06 mL, 0.67 mmol) were added. After 5 min, EDC·HCl (0.23 g, 1.22 mmol) and HOBT (24 mg, 0.18 mmol) were added and the reaction was continued with stirring at rt for 1 h. After complete consumption of starting materials solvent

was removed and the crude residue was purified on silica gel (50% ethyl acetate: petroleum ether) to obtain desired product 14 as a colorless foam (0.183 g, 78% over two steps).

[α]$_D^{25}$ +12.5 (c = 0.15, $CHCl_3$).

IR (cm^{-1}, $CHCl_3$) ν 2949, 2693, 2320, 2110, 1733, 1204, 1025, 920.

^{1}H NMR (400 MHz, $CDCl_3$) δ 7.51-7.49 (m, 2H, ArH), 7.34-7.28 (m, 3H, ArH), 6.71 (d, J = 9.3 Hz, 1H, NH), 5.16 (dd, J = 10.5, 3.0 Hz, 1H, H-3), 4.92 (d, J = 10.5 Hz, 1H, H-1), 4.30 (q, J = 10.5 Hz, 1H, H-2), 3.95, 3.92 (ABq, J = 10.1 Hz, 2H, CH_2Az), 3.87 (d, J = 2.7 Hz, 1H, H-4), 3.79 (q, J = 6.4 Hz, 1H, H-5), 2.85 (bs, 1H, OH), 2.11 (s, 3H, OAc), 1.35 (d, J = 6.4 Hz, 3H, H-6).

^{13}C{1H} NMR (100 MHz, $CDCl_3$) δ 171.1, 167.5, 133.1, 132.2, 129.1, 128.0, 86.7, 74.8, 74.2, 69.8, 52.7, 49.5, 21.0, 16.7.

HRMS (ESI-TOF) (m/z): $[M+Na]^+$ calcd. for $C_{16}H_{20}N_4O_5SNa$ 403.1047; found, 403.1047.

Compound 5:

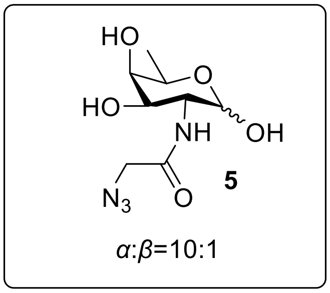

NBS (0.1 g, 0.59 mmol) was added to the solution of **14** (0.15 g, 0.39 mmol) in THF: H2O (10.0 mL, 9:1) and stirred for 10 min. After complete consumption of starting material, reaction mixture was diluted with EtOAc and washed with aq. $Na_2S_2O_3$. Separated organic layer was dried over Na_2SO_4 and concentrated and pass through small silica column and compound was concentrated. NaOMe (30 mg) was added to clear solution of the above obtained compound in MeOH (3 mL) and stirred at rt for 10 min. After complete consumption of starting material, reaction mixture was neutralized with Amberlite (H+, 0.1 g) and the reaction mixture was filtered and concentrated. The product was purified by column chromatography on silica gel (10% methanol:ethyl acetate) to afford the desired product **5** as a white foamy solid (75 mg, α:β=10:1 78% over two steps).

[α]$_D^{25}$ +3.20 (c = 0.1, MeOH).

IR (cm^{-1}, $CHCl_3$) ν 3487, 2156, 1706, 1463, 1046, 798.

^{1}H NMR (400 MHz, CD_3OD)) (for α isomer) δ 5.10 (d, J = 3.5 Hz, 1H, H-1), 4.24-4.16 (m, 2H, H-2, H-5), 3.95-3.94 (m, 2H), 3.83 (dd, J = 10.8, 3.1 Hz, 1H, H-3), 3.68 (s, 1H), 1.23 (d, J = 6.6 Hz, 3H, CH_3).

^{13}C{1H} NMR (100 MHz, CD_3OD) (for α isomer) δ 169.2, 91.3, 71.7, 68.4, 65.7, 51.5, 50.4, 15.5.

HRMS (ESI-TOF) (m/z): $[M+Na]^+$ calcd. for $C_8H_{14}N_4NaO_5$ 269.0856; found, 269.0857.

Compound 7:

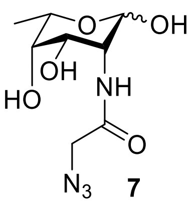

7

α:β=2:1

Compound **15**[5] (0.092 g, 0.21 mmol) was dissolved in CH_3CN:H_2O (6 mL: 1 mL) and CAN (0.462 g, 0.84 mmol) was added to it at RT. After 4 h, reaction mixture was diluted with ethyl acetate and transferred into a separating funnel. Organic layer was washed with $NaHCO_3$ (3 ×10 mL), saturated $Na_2S_2O_3$ solution and water, the collected organic layer was dried over Na_2SO_4, concentrated and passed through a short silica column (70% ethyl acetate:pet ether) to afford hemiacetal as yellowish sticky liquid. Purified hemiacetal was dissolved in MeOH (1 mL) and NaOMe (0.010 g) was added to it and stirred at RT for 2 hours. After complete consumption of starting material reaction mixture was neutralized with amberlite (H+) and the reaction mixture was filtered, concentrated under the reduced pressure. Reaction mixture was directly concentrated after checking the completion of the reaction by TLC and purified by column chromatography eluting in 5% methanol:ethyl acetate to give compound **7** (34 mg, α:β=2:1, 64% over two steps) as a yellow viscous liquid.

$[\alpha]_D^{25}$ +5.20 (c = 0.2, $CHCl_3$).

IR (cm^{-1}, MeOH) ν 3500, 2109, 1654, 1463, 1036, 758.

^{1}H NMR (400 MHz, CD_3OD) δ 5.07 (d, J = 3.6 Hz, 1H, H-1α), 4.99 (s, 0.5H, H-1β), 4.21-4.16 (m, 2H), 4.12-4.08 (m, 1H), 4.02-4.00 (m, 1H), 3.98-3.88 (m, 2H), 3.82-3.74 (m, 1.5H), 3.66-3.58 (m, 1.5 H), 1.23 (m, 4.5 H, CH_3).
^{13}C{1H} NMR (100 MHz, CD_3OD) δ 169.1, 168.6, 93.6, 91.3, 71.7, 71.6, 68.4, 66.0, 65.7, 63.9, 52.6, 52.1, 51.5, 50.4, 15.6, 15.5.
HRMS (ESI-TOF) (m/z): $[M+H]^+$ calcd. for $C_8H_{15}N_4O_5$ 247.1037; found 247.1038.

Compound 9:

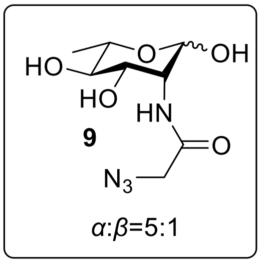

Compound **16**[5] (0.092 g, 0.21 mmol) was dissolved in CH_3CN: H_2O (6 mL:1 mL) and CAN (0.462 g, 0.84 mmol) was added to it at RT. After 4 h, reaction mixture was diluted with ethyl acetate and transferred into a separating funnel. Organic layer was washed with $NaHCO_3$ (3 ×10 mL), saturated $Na_2S_2O_3$ solution and water, the collected organic layer was dried over Na_2SO_4, concentrated and passed through a short silica column to afford hemiacetal compound. Purified hemiacetal was dissolved in MeOH (1 mL) and NaOMe (0.010 g) was added to it and stirred at RT for 2 hours. After complete consumption of starting material reaction mixture was neutralized with amberlite (H+) and the reaction mixture was filtered, concentrated under the reduced pressure. Reaction mixture was directly concentrated after checking the completion of the reaction by TLC and purified by column chromatography eluting in 5% methanol:ethylacetate to give compound **9** (32 mg, α:β = 5:1, 61% over two steps) as a yellow viscous liquid.
$[\alpha]_D^{25}$ +0.96 (c 0.06, MeOH).
IR (cm^{-1}, MeOH) ν 3476, 2906, 2113, 1656, 1451.
^{1}H NMR (400 MHz, CD_3OD) (for α isomer) δ 4.97 (d, J = 3.4 Hz, 1H, H-1), 3.86-3.85 (m, 2H), 3.82-3.78 (m, 2H), 3.61-3.56 (m, 1H), 2.97 (t, J = 8.9 Hz, 1H), 1.16 (d, J = 6.2 Hz, 3H, CH_3).
^{13}C{1H} NMR (100 MHz, CD_3OD) (for α isomer) δ 169.0, 91.0, 76.7, 71.1, 66.9, 54.7, 51.4, 16.8.
HRMS (ESI-TOF) (m/z): $[M+H]^+$ calcd. for $C_8H_{15}N_4O_5$ 247.1037, found 247.1038.

Compound 19:

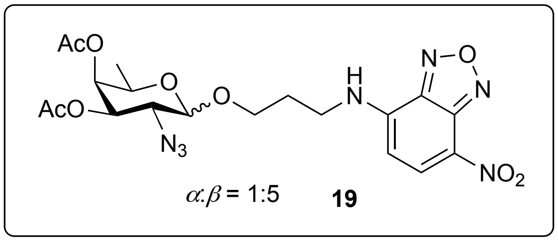

NBS (0.099 g, 0.558 mmol) was added to the stirred solution of compound **18**[2] (0.068 g, 0.186 mmol) in 2 mL THF/H_2O (5:1) at 0 °C. After 15 min, the reaction mixture was diluted with EtOAc and washed with aqueous $Na_2S_2O_3$ solution. The separated organic layer was dried over anhydrous Na_2SO_4, filtered, and concentrated. The crude compound was washed with hexane to remove the non-polar impurity and taken to next step. To the hemiacetal compound in anhydrous CH_2Cl_2 (2 mL), CCl_3CN (0.09 mL, 0.930 mmol) and K_2CO_3 (0.128 g, 0.930 mmol) was added. The reaction was stirred at RT for 6 h. After complete consumption of starting material, unreacted K_2CO_3 was filtered out using celite. The filtrate containing the formed glycosyl imidate donor was concentrated and kept in vacumn prior to glycosylation.

Imidate donor (0.077 g, 0.186 mmol), acceptor **17**[11] (0.066 g, 0.279 mmol) and and molecular sieves (3Å, 200 mg) were dissolved in anhydrous CH_3CN. Then, TfOH (5 µl, 0.055 mmol) was added dropwise to the solution at 0 ºC. After 2 h, with completion of reaction, mixture was quenched by Et_3N and molecular sieves were filtered out through Celite. Then crude was concentrated and purified by flash column chromatography to afford fluorescent compound **19** (0.041 g, 45% yield over three steps as α:β = 0.2:1) as sticky liquid having fluorescent green colour.

$[\alpha]_D^{25}$ +14.4 (c = 0.15, $CHCl_3$).

IR (cm^{-1}, $CHCl_3$) ν 3554, 2859, 2105, 1764, 1551, 1350, 1089, 504.

^{1}H NMR (400 MHz, $CDCl_3$) δ 8.51 (d, 1H, J = 8.0 Hz, CHArβ), 8.50 (d, 0.2H, J = 8.0 Hz, CHArα), 7.07 (1H, bs, NH), 6.20 (d, 1H, J = 8.8 Hz, CHArβ), 6.19 (d, 0.2H, J = 8.0 Hz, CHArα), 5.40 (dd, 0.2H, J = 8 Hz, 4 Hz, H-3α), 5.33-5.22 (m, 0.2H, H-4α), 5.23 (dd, 1H, J = 4 Hz, 2 Hz, H-4β), 4.97 (d, 0.2H, J = 2 Hz, H-1α), 4.85 (dd, 1H, J = 8Hz, 4Hz, H-3β), 4.39 (d, 1H, J = 8 Hz, H-1β), 4.22- 4.13 (m, 1H, H-5β), 3.90-3.65 (m, 5H), 2.22 (s, 3H, OAc), 2.12-2.16 (m, 2H), 2.10 (s, 3H, OAc), 1.25 (d, 3H, J = 6.4 Hz, H-6β), 1.15 (d, 0.6H, J = 6.4 Hz, H-6α).

^{13}C{1H} NMR (100 MHz, $CDCl_3$) δ 170.5, 169.9, 144.4, 144.0, 143.9, 136.6, 102.2, 98.3, 98.1, 71.6, 70.4, 69.8, 69.5, 69.4, 68.8, 67.4, 65.3, 60.7, 57.7, 42.2, 29.6, 28.1, 27.8, 20.67, 20.63, 16.1, 15.9.

HRMS (ESI-TOF) (m/z): $[M+Na]^+$ calcd. for $C_{19}H_{23}N_7NaO_9$ 516.1449; found, 516.1446.

Compound 12:

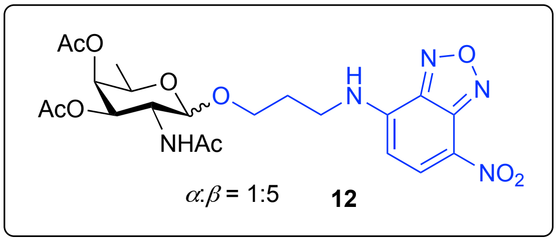

Compound **19** (0.067 g, 0.140 mmol) was taken in THF (2 ml) and PMe_3 in 1M toluene (0.337 mmol, 0.3 ml) was added to it. After, stirring for 30 mins, a fluorescent polar spot is observed. Reaction mixture was concentrated, azeotroped with toluene and kept in vacumn. For next step, crude reaction mixture was taken in THF (1.5 ml), Ac_2O (0.05 ml, 0.560 mmol) and Et_3N (0.05 ml) were added sequentially and stirred at RT for 1 h. After completion of reaction, reaction mixture was concentrated in vacuo and purified through column chromatography (10% Methanol-EA eluted the product) to give compound **12** (0.038 g, 54% yield over two steps as α:β =1:5).

$[\alpha]_D^{25}$ +13.5 (c = 0.15, $CHCl_3$).

IR (cm^{-1}, $CHCl_3$) ν 3459, 2859, 1760, 1547, 1342, 1237, 489.

^{1}H NMR (500 MHz, MeOD) δ 8.56 (d, 1H, J = 8.8 Hz, CHAr), 6.41 (d, 1H, J = 8.8 Hz, CHAr), 5.24 (dd, 0.2 H, J = 10 Hz, 5 Hz, H-4α), 5.21-5.17 (m, 1.3H, H-4β, H-1α), 5.05 (dd, 1H, J = 15 Hz, 5Hz, H-3β), 4.54 (d, 1H, J = 10 Hz, H-1β), 4.46 (dd, 0.2H, J = 10 Hz, 3 Hz, H-3α), 4.16-4.11 (m, 1H), 4.03-3.98 (m, 1H), 3.92-3.87 (m, 1H), 3.69-3.62 (m, 4Hz), 2.20 (s, 3H, OAcβ), 2.17 (s, 0.6H, OAcα), 2.05-2.03 (m, 2H), 1.98 (s, 3H, OAcβ), 1.97 (s, 0.6H, OAcα), 1.95 (s, 3H, OAcβ), 1.16 (d, 3H, J = 6.4 Hz, H-6β), 1.10 (d, 0.6H, J = 6.4 Hz, H-6α).

^{13}C{1H} NMR (125 MHz, MeOD) δ 172.3, 171.0, 170.4, 101.3, 97.6, 71.1, 70.4, 69.8, 68.8, 68.7, 66.4, 64.6, 50.0, 21.5, 19.2, 19.1, 15.1.

HRMS (ESI-TOF) (m/z): $[M+H]^+$ calcd. for $C_{21}H_{28}N_5NaO_{10}$ 510.1840; found, 510.1839.

Biology

General. All biological reagents were obtained from commercial suppliers (*e.g.,* MilliporeSigma, Fisher, ThermoFischer Scientific, Bio-Rad, Vector Laboratories) and used without further purification. All bacterial cells were purchased from ATCC except for *Helicobacter pylori* strain G27,[3] which was a gift from Manuel Amieva (Stanford University). Peracetylated *N*-acetylglucosamine (Ac_4GlcNAc), peracetylated and free *N*-azidoacetylglucosamine (Ac_4GlcNAz and GlcNAz), and peracetylated and free *N*-azidoacetylgalactosamine (Ac_4GalNAz and GalNAz) were synthesized per established protocols.[4] Peracetylated *N*-azidoacetyl analogs of rare sugars D-fucosamine (D-Ac_3FucNAz), L-acetylpneumosamine (L-Ac_3PneNAz), and L-acetylrhamnosamine (L-Ac_3RhaNAz) were synthesized according to our published procedures.[1,5] Alexa Fluor 488-dibenzocyclooctyne (AF488-DIBO) was purchased from Fisher Scientific (Waltham, MA).

Bacterial strains and culture conditions. *Plesiomonas shigelloides* American Type Culture Collection (ATCC) strain 51903 was grown on Brain-Heart Infusion (BHI) plates (1.5% Bacto agar, 3.7% brain-heart infusion media, 0.5% yeast extract) overnight at 37°C under aerobic conditions. *Vibrio vulnificus* ATCC strain 43382 was grown on Luria Bertani (LB) plates (1.5% Bacto agar, 2.4% Luria-Bertani broth powder) overnight at 37°C under aerobic conditions. *Helicobacter pylori* strain G27 was grown on horse blood agar (HBA) plates for 3-4 days at 37°C under microaerophilic conditions (14% CO_2). *Bacteroides fragilis* ATCC 23745 was grown on BHI plates with hematin (1.5% Bacto agar, 3.7% brain-heart infusion media, 0.5% yeast extract, 0.0015% hematin) for 2 days under anaerobic conditions with an AnaeroGen 2.5L packet (Thermo Fisher Scientific, Waltham, MA) at 37°C.

Metabolic glycan labeling. Bacteria were inoculated into 3.5 mL liquid culture (BHI broth (3.7% brain-heart infusion media, 0.5% yeast extract) for *P. shigelloides*; LB broth (2.4% Luria-Bertani broth powder) for *V. vulnificus*; Brucella broth (2.81% Brucella Broth, 10% fetal bovine serum, 0.0006% vancomycin) for *H. pylori*; BHI broth with hematin (3.7% brain-heart infusion media, 0.5% yeast extract, 0.0015% hematin) for *B. fragilis*) to an optical density measured at 600 nm of

0.3-0.4 prior to treatment with 1 mM of Ac_4GlcNAc **11** as an azide-free negative control that would not be visualized using bioorthogonal SPAAC-based assays, or with 1 mM of one of the following azidosugars: Ac_4GlcNAz **1**, GlcNAz **2**, GalNAz **3**, Ac_4GalNAz **4**, FucNAz **5**, Ac_3FucNAz **6**, PneNAz **7**, Ac_3PneNAz **8**, RhaNAz **9**, or Ac_3RhaNAz **10**. Ac_4GlcNAz and Ac_4GalNAz were included as positive controls due to established metabolic incorporation in the bacterial species used in that experiment. In particular, *B. fragilis* is known to metabolically incorporate Ac_4GalNAz robustly into its capsular polysaccharide A,[6] and the other bacteria metabolically incorporate Ac_4GlcNAz to an appreciable extent.[5, 7] The bacteria were cultured for 1-3 days with gentle agitation under aerobic (*P. shigelloides*, *V. vulnificus*), microaerophilic (*H. pylori*), or anaerobic (*B. fragilis*) conditions, according to their optimal conditions. Following metabolic labeling, azidosugar incorporation in bacterial cells was measured by flow cytometry as described below.

Flow cytometry detection of azide-labeled cells. Flow cytometry was performed to measure incorporation of azido sugars into cell surface glycans on live cells. After metabolic labeling, bacteria in liquid culture were centrifuged at 2000 x *g* for 17 minutes at room temperature and washed 3 times in 3 mL 1X phosphate-buffered saline (PBS) before reaction with AF488-DIBO (5 µM) for 1 hour at 37°C in the dark. Samples were then rinsed 3 times in PBS prior to flow cytometry analysis. Whole cell samples were analyzed by BD Accuri C6 Plus flow cytometer (BD Biosciences, Franklin Lakes, NJ), with 10,000 live cells counted in each of three biological replicates, and the raw data were analyzed with FlowJo™ Software (BD Biosciences, Franklin Lakes, NJ). Each metabolic labeling experiment was repeated independently, and figures show representative histograms.

Bacterial cell lysis. Following 1-3 days of metabolic labeling, *P. shigelloides*, *V. vulnificus*, *H. pylori*, and *B. fragilis* were centrifuged at 2000 x *g* for 17 minutes at 20°C, then washed 3 times in 3 mL 1X PBS. After the third wash, the cells were resuspended in 50-75 µL lysis buffer (20 mM Tris base pH 7.4, 1% Igepal, 150 mM NaCl, 1 mM EDTA) with 1X protease inhibitor cocktail (Sigma Aldrich, St. Louis, MO) and lysed with vigorous pipetting. The cell lysate solution was subjected to three freeze thaw cycles (alternating -80°C and 37°C for 10 minutes each or an ethanol and dry ice bath and 37°C for 5 minutes each) prior to water bath sonication (Ultrasonic Bath 1.9 L, Thermo Fisher Scientific, Waltham, MA) for 30 minutes. Cell lysates were centrifuged at 17000

x *g* for 15 minutes and the supernatant containing soluble proteins was separated. Supernatants were collected and stored at -20°C for further analysis. Protein concentration of clarified lysates was determined by *DC* Protein Assay (Bio-Rad, Hercules, CA) and standardized to a protein concentration 3.0 mg/mL for use in experiments to measure esterase activity.

Mammalian cell growth. Human leukemia-derived THP-1 monocytes were grown to 10^6 cells/mL in 10 mL warm RPMI 1640 with 20% FBS at 37°C and 5% CO_2. The cells were then centrifuged at 100 x g for 10 min, and the pellet was resuspended in 10 mL of the media again before adding to T-25 flasks to grow in 37 °C and 5% CO_2. After three days, cell viability was checked and cells were counted. Cells were subsequently diluted at a split of 1:3 – 1:5 with fresh RPMI media every 3-4 days to prevent over-crowding in T-75 flasks. THP-1 cells and lysates were used as positive controls in esterase activity assays.

Detection of esterase activity using fluorescein diacetate deacetylation. Fluorescence generated by hydrolysis of fluorescein diacetate (FDA, MilliporeSigma, Burlington, MA) by cellular esterases was used as a proxy to measure esterase activity in whole cells and cell lysates.[9] A stock solution of FDA (6 mM in DMSO) was serially diluted to 60 µM, 6 µM, and 0.6 µM in 1X PBS. Both flow cytometry and plate reader assay protocols were validated by experiments with THP-1 cells, cells known to have high esterase activity.[10] Bacteria from agar plates (BHI for *P. shigelloides*; LB for *V. vulnificus*; HBA for *H. pylori*) were inoculated into 3.5 mL liquid culture (BHI broth for *P. shigelloides*; LB broth for *V. vulnificus*; Brucella broth for *H. pylori*) to an optical density of 1.0 at 600 nm. THP-1 cells were harvested at 10^6 cells/mL. For live cell harvest, cells in liquid culture were centrifuged (2000 x *g* for 17 minutes for bacteria, 100 x *g* for 15 minutes for mammalian cells) before washing once in 3 mL PBS. Washed cells were incubated in the dark with FDA (0.6 µM, 6 µM, or 60 µM in 1% BSA in 1X PBS) for 15 minutes at 37°C. Treated samples were then aliquoted into three replicates and analyzed by flow cytometry. Whole cell samples were analyzed by BD Accuri C6 Plus flow cytometer (BD Biosciences, Franklin Lakes, NJ), with 10,000 live cells counted in each of three biological replicates, and the raw data were analyzed with FlowJo™ Software (BD Biosciences, Franklin Lakes, NJ). Each FDA experiment was repeated independently, and figures show representative histograms.

For analysis of esterase activity in cell lysates, bacterial and mammalian cells were lysed as described above. Standardized lysates (3 mg/mL) were incubated with dH_2O (0 µM FDA) or 0.6 µM FDA in dH_2O for 15 minutes in a 96-well plate. Fluorescence resulting from FDA hydrolysis was detected by GloMax® Discover Microplate Reader (Promega, Madison, WI). Fluorescence of lysis buffer without bacterial lysates treated with 0 µM or 0.6 µM FDA was measured as a negative control, and THP-1 lysates treated with 0.6 µM FDA served as a positive control.[9]

Detection of esterase activity using NBD-FucNAc deacetylation. Bacterial cells were lysed and standardized to 3 mg/mL protein in lysis buffer, then incubated overnight in lysis buffer containing 0.4 mM NBD-FucNAc or no additional supplement at 37°C. Samples were filtered through Pierce concentrator PES filter (#88513, PES, 10K MWCO, 0.5mL, ThermoFisher Scientific, Waltham, MA;) during a 50-minute spin at 7500 x *g* to separate proteins from small molecules, and flow-through was subsequently collected for further analysis. NBD-FucNAc probes were enriched from samples by chromatography on a Sep-Pak 3-cc C18 cartridge (Supelco DSC-18 3 mL, 500 mg, #52603-U, MilliporeSigma) using a vacuum manifold. The column was wet twice with 2 mL acetonitrile and equilibrated with four washes of 2 mL 0.1% trifluoroacetic acid (TFA) in water. Flow-through was loaded onto the column and the column was washed four times with 2 mL 0.1% TFA in water. Finally, compounds were eluted with 3 applications of 600 µL elution buffer containing 50% acetonitrile and 0.1% TFA in water. Visual analysis revealed the presence of a yellow substance in the second and third elutions, which indicated fractions likely to contain NBD-FucNAc and its derivatives. Solvent was removed from these elutions *in vacuo* (Thermo Vacuum Concentrator SPD111V and Savant Universal SpeedVac System UVS400, ThermoFisher Scientific, Waltham, MA).

Following solvent removal, the remaining material was resuspended in 5% acetonitrile 95% water for analysis by high performance liquid chromatography (HPLC, Agilent 1100 Series HPLC System, Agilent Technologies, Santa Clara, CA). Samples were first sonicated for 30 minutes in a water bath sonicator and then chromatographed using a reverse-phase BDS HYPERSIL C18 250 x 3 mm column (SN 0405836T, part 28105-253030, 5 µm particle size, ThermoFisher Scientific, Waltham, MA). An elution gradient of 0.1% H_3PO_4 in LCMS-grade water (solvent A) and 0.1% H_3PO_4 in LCMS-grade acetonitrile (solvent B, see Table S1) and a flow rate of 0.5 mL/min were

used for all analyses. Material on the column was detected by Agilent 1100 series diode-array detector (DAD) and fluorescence detector (FD) (DAD G1315B; FD G1321A, λ_{ex} 460 nm, λ_{em} 530 nm; Agilent Technologies, Santa Clara, CA). Alongside samples, a 5% acetonitrile blank and a 0.1 mM NBD-FucNAc synthetic standard were analyzed for comparative purposes and peak assignment.

Table S1. Solvent gradient used for HPLC. Aqueous phase solvent A is 0.1% H_3PO_4 in H_2O and organic phase solvent B is 0.1% H_3PO_4 in acetonitrile.

Time (min)	Solvent A (%)	Solvent B (%)
**0.0**	99.5	0.5
**3.0**	99.5	0.5
**38.0**	20.0	80.0
**45.0**	10.0	90.0
**59.0**	99.5	0.5
**10 min postrun**	99.5	0.5

Chip-based nanoLC-MS/MS analysis of *H. pylori* treated with NBD-FucNAc. Wild-type *H. pylori* cells were grown in Brucella Broth growth media (10% FBS, 6 µg/mL vancomycin) supplemented with 0.5 mM NBD-FucNAc in microaerophilic conditions (14% CO_2, 37ºC) for three days. After incubation, conditioned media was collected and samples were filtered through Pierce concentrator PES filters and flow-through was subsequently collected prior to enrichment of NBD-FucNAc from samples by chromatography on a Sep-Pak 3-cc C18 cartridge as described in "Detection of esterase activity using NBD-FucNAc deacetylation" above. Liquid chromatography-mass spectrometry (LC-MS) was performed on NBD-FucNAc enriched from conditioned media using a 6530 Accurate-Mass Quadrupole Time-of-Flight (Q-TOF) LC-MS (Agilent Technologies). Liquid chromatography was performed using a 1260 HPLC Chip Cube interface, and nanoelectrospray ionization (nanoESI) analysis was conducted using an HPLC-Chip with a 40 nL enrichment column and 150 mm x 75 µm separation column packed with Zorbax 300SB-C18 stationary phase (5 µm particles and 300 Å pore size). A gradient of 0.1% formic acid in LC/MS-grade water (Solvent A) and 0.1% formic acid and 2% water in ACN (Solvent B, see Table 2) and a flow rate of 0.3 µL/min was used for all analyses.

Table S1. Gradient conditions used for LC-MS analysis. Solvent A was 0.1% formic acid in water and solvent B was 0.1% formic acid and 2% water in acetonitrile.

Time (min)	Solvent A (%)	Solvent B (%)
0	99.5	0.5
3.00	99.5	0.5
20.00	65.0	35.0
35.00	30.0	70.0
35.10	20.0	80.0
40.00	20.0	80.0
40.10	99.5	0.5

Mass spectra (MS and MS/MS) were collected in positive ion mode using ionization voltage in the range of 1850-1975 V. The ion source temperature was held at 350 ºC. Spectra were internally calibrated using dibutyl phthalate ($C_{16}H_{22}O_4$) and hexakis(1H, 1H, 4H-hexafluorobutyloxy)phosphazine (HP-1221; $C_{24}H_{18}O_6N_3P_3F_{36}$), continuously evaporated and detected as $[M+H]^+$. MS/MS experiments were conducted with precursor ions subjected to collision-induced dissociation (CID) using nitrogen as the target gas. Analyses of MS and MS/MS spectra were conducted with the MassHunter Workstation Qualitative Analysis Software (Agilent; Version 10.0).

Supplemental Figures

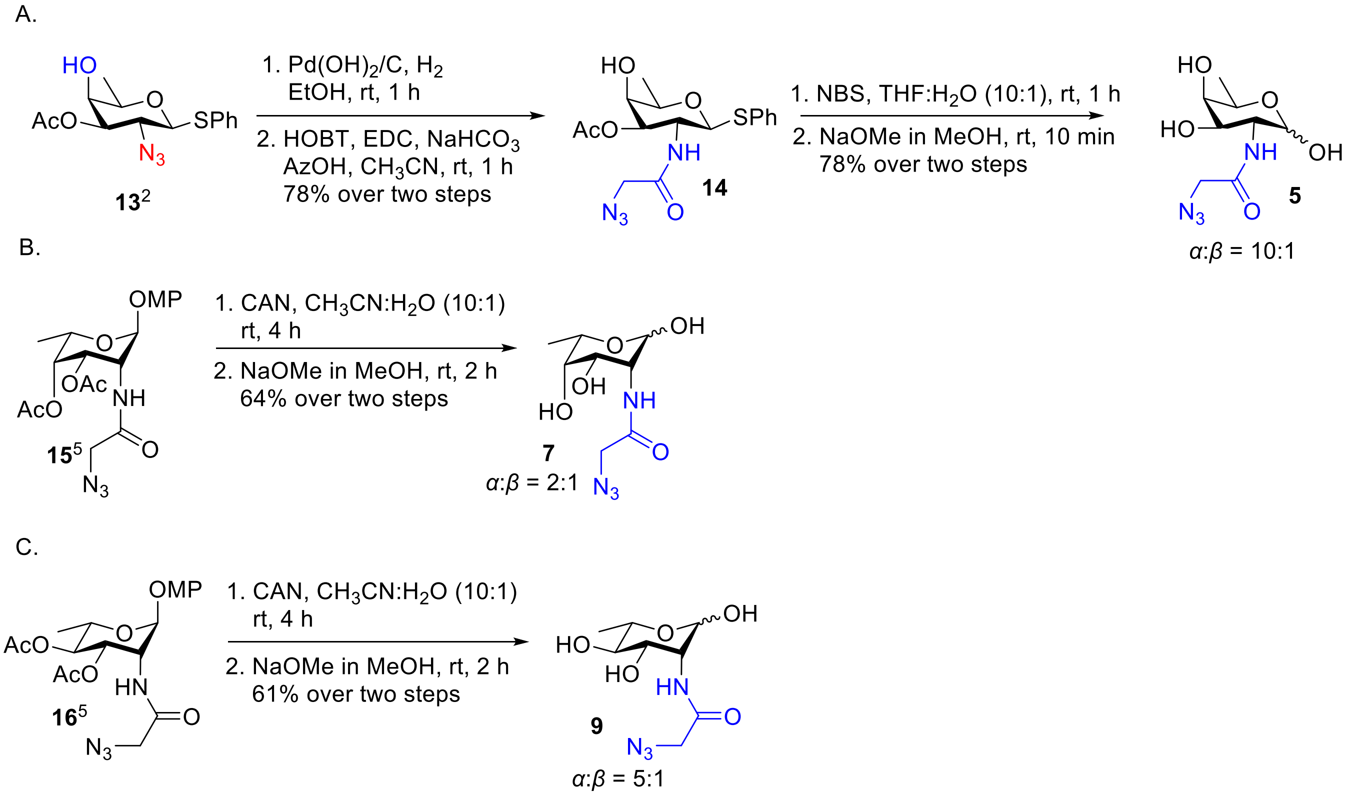

Figure S1. Synthesis of (A) FucNAz **5,** (B) PneNAz **7** and (C) RhaNAz **9**.

Ac_3FucNAz **6**[8], Ac_3PneNAz **8**[5] and Ac_3RhaNAz **10**[5] were synthesized using reported protocols.

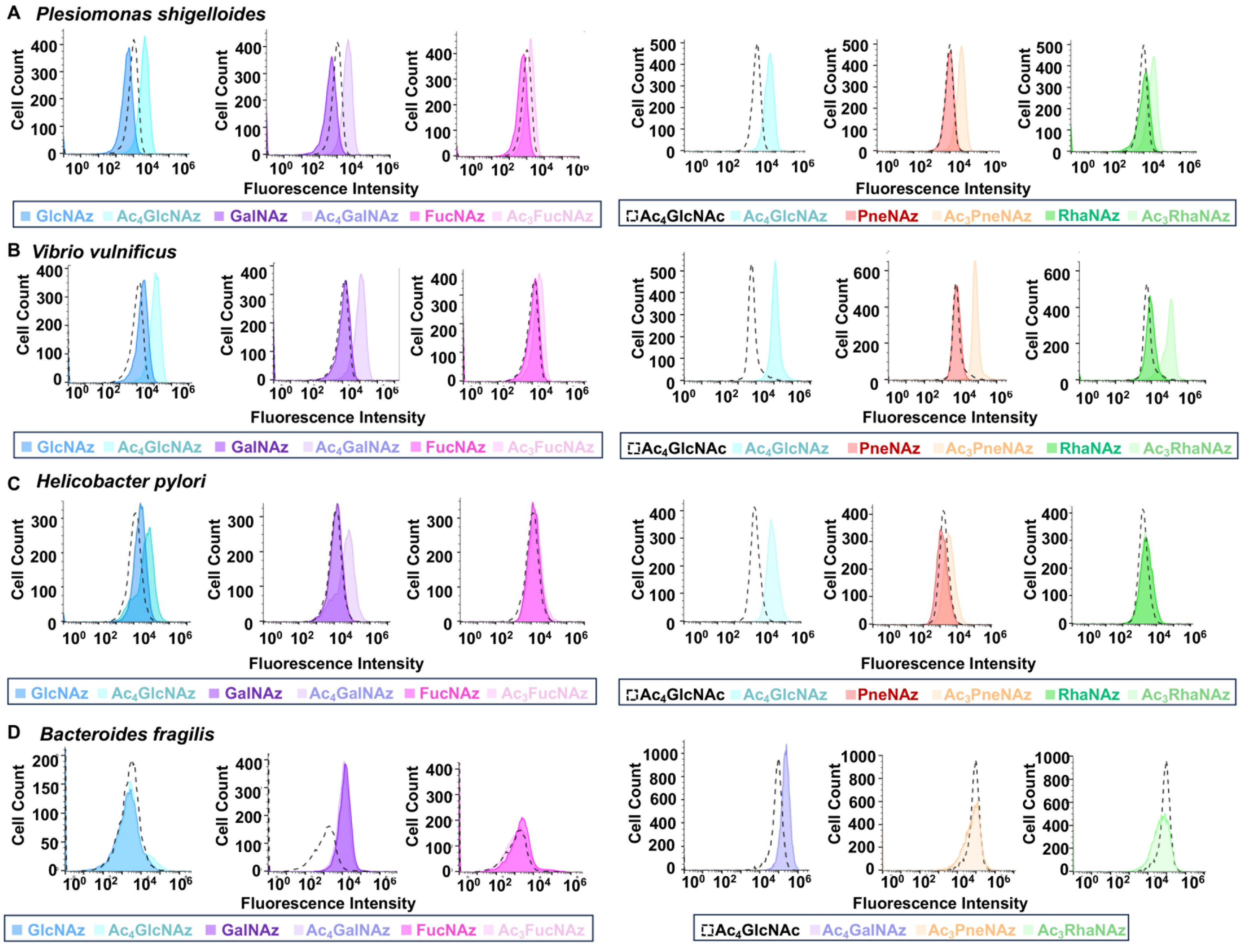

Figure S2. Metabolic labeling of bacterial cells treated with peracetylated versus unprotected D- and L-azidosugars was assessed via a flow cytometry assay following reaction of azide-labelled cells with AF488-DBCO. Fluorescence of treated A) *Plesiomonas shigelloides*, B) *Vibrio vulnificus*, C) *Helicobacter pylori*, and D) *Bacteroides fragilis* was detected by flow cytometry and is shown as histograms of fluorescence intensity versus cell count. Data are representative of replicate experiments and correspond to those shown in Figure 2 of the main manuscript.

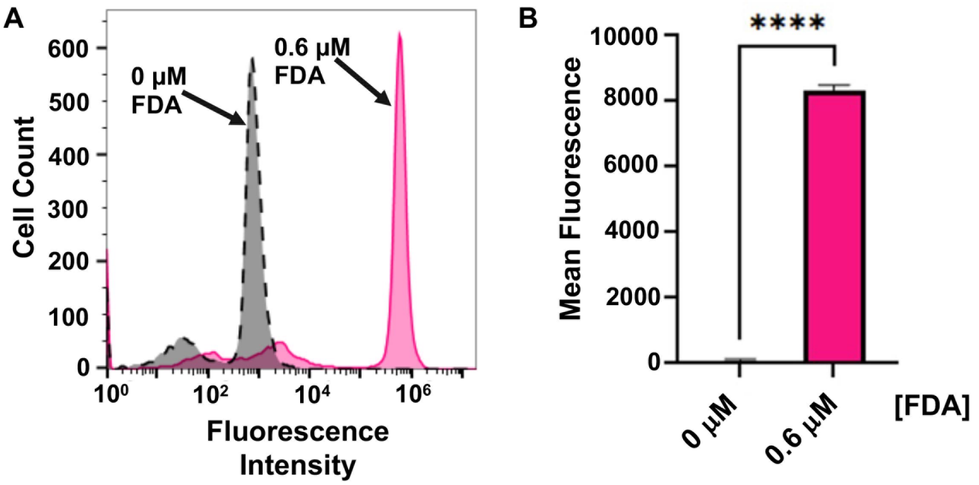

Figure S3. THP-1 cells and lysates treated with fluorescein diacetate led to high levels of fluorescence indicative of esterase-mediated cleavage of acetates to yield fluorescein. A) THP-1 cells were incubated with 0.6 µM fluorescein diacetate (FDA) or no FDA for 15 minutes, then analyzed by flow cytometry B) THP-1 lysates (3 mg/mL protein) were harvested and incubated with 0.6 µM fluorescein diacetate (FDA) or no FDA for 15 minutes, then analyzed on a fluorescence plate reader. Esterase-catalyzed hydrolysis of FDA occurred in both (A) whole cells and (B) cell lysates. Statistical significance was determined by one-way ANOVA test. **** $p < 0.0001$.

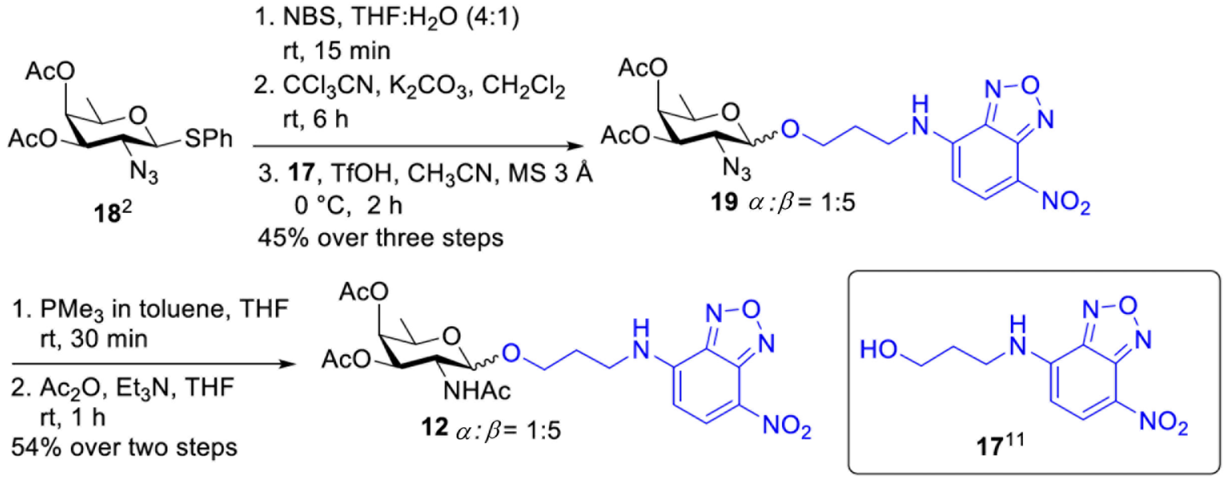

Figure S4. Synthesis of NBD-FucNAc **12**.

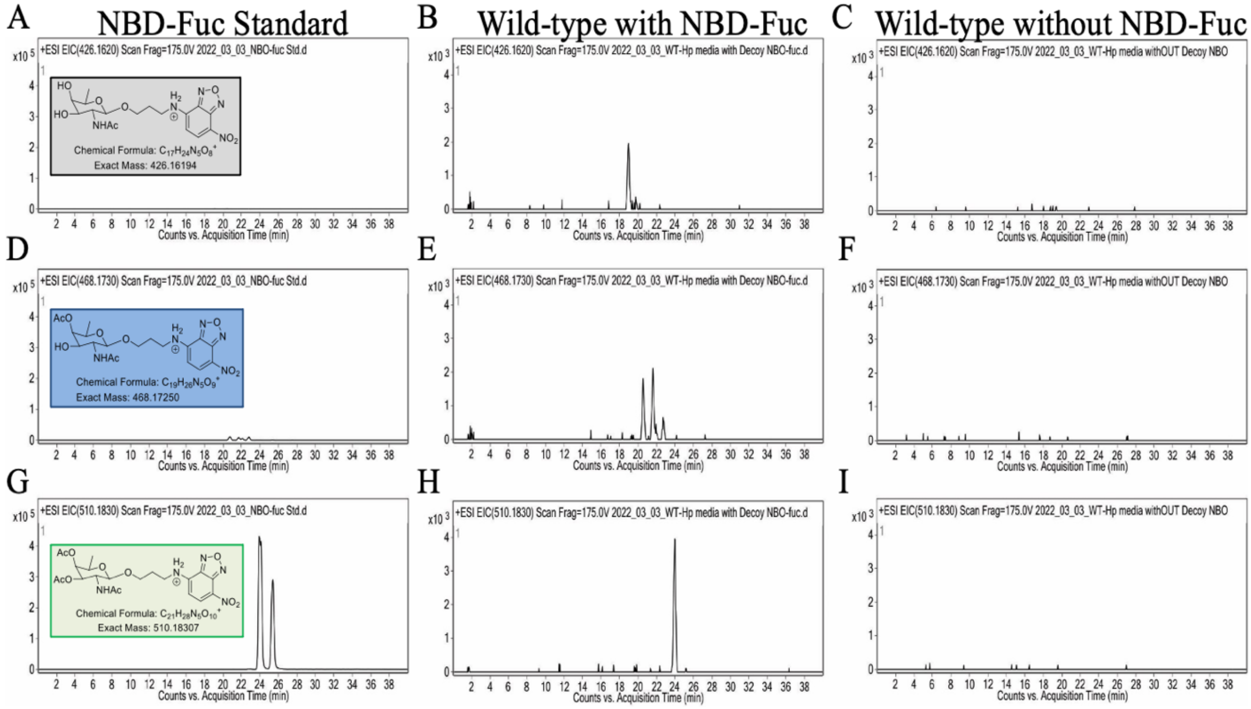

Figure S5. Extracted Ion Chromatograms (EIC) reveal that NBD-FucNAc 12 had been mono-deacetylated and di-deacetylated by *H. pylori*. LC-MS/MS analysis was performed on and NBD-FucNAc standard and conditioned media from *H. pylori* treated with or without NBD-FucNAc. EICs were developed for (A-C) the di-deacetylated form of NBD-FucNAc, (D-F) the mono-deacetylated form of NBD-FucNAc, and (G-I) the fully intact form of NBD-FucNAc for each condition. Compound identity was confirmed using exact mass measurements and the analysis of MS/MS spectra (data not shown).

Supplemental References

(1) Sissoko, A.; Vásquez-Ocmín, P.; Maciuk, A.; Barbieri, D.; Neveu, G.; Rondepierre, L.; Grougnet, R.; Leproux, P.; Blaud, M.; Hammad, K.; et al. A chemically stable fluorescent mimic of dihydroartemisinin, artemether, and arteether with conserved bioactivity and specificity shows high pharmacological relevance to the antimalarial drugs. *ACS Infectious Diseases* **2020**, *6* (7), 1532-1547.
(2) Williams, D. A.; Pradhan, K.; Paul, A.; Olin, I. R.; Tuck, O. T.; Moulton, K. D.; Kulkarni, S. S.; Dube, D. H. Metabolic inhibitors of bacterial glycan biosynthesis. *Chem Sci* **2020**, *11* (7), 1761-1774.
(3) Baltrus, D. A.; Amieva, M. R.; Covacci, A.; Lowe, T. M.; Merrell, D. S.; Ottemann, K. M.; Stein, M.; Salama, N. R.; Guillemin, K. The complete genome sequence of *Helicobacter pylori* strain G27. *J. Bacteriol.* **2009**, *191* (1), 447-448.
(4) Laughlin, S. T.; Bertozzi, C. R. Metabolic labeling of glycans with azido sugars and subsequent glycan-profiling and visualization via Staudinger ligation. *Nature Protocols* **2007**, *2* (11), 2930-2944.
(5) Luong, P.; Ghosh, A.; Moulton, K. D.; Kulkarni, S. S.; Dube, D. H. Synthesis and application of rare deoxy amino L-sugar analogues to probe glycans in pathogenic bacteria. *ACS Infectious Diseases* **2022**, *8* (4), 889-900.
(6) Shalizi, A.; Wiegers, T. N.; Maamar, H. Click-to-Capture: A method for enriching viable *Staphylococcus aureus* using bio-orthogonal labeling of surface proteins. *PLoS One* **2020**, *15* (6), e0234542.
(7) Geva-Zatorsky, N.; Alvarez, D.; Hudak, J. E.; Reading, N. C.; Erturk-Hasdemir, D.; Dasgupta, S.; von Andrian, U. H.; Kasper, D. L. In vivo imaging and tracking of host-microbiota interactions via metabolic labeling of gut anaerobic bacteria. *Nat Med* **2015**, *21* (9), 1091-1100.
(8) Clark, E. L.; Emmadi, M.; Krupp, K. L.; Podilapu, A. R.; Helble, J. D.; Kulkarni, S. S.; Dube, D. H. Development of rare bacterial monosaccharide analogs for metabolic glycan labeling in pathogenic bacteria. *ACS Chemical Biology* **2016**, *11* (12), 3365-3373.
(9) Breeuwer, P.; Drocourt, J. L.; Bunschoten, N.; Zwietering, M. H.; Rombouts, F. M.; Abee, T. Characterization of uptake and hydrolysis of fluorescein diacetate and carboxyfluorescein diacetate by intracellular esterases in *Saccharomyces cerevisiae*, which result in accumulation of fluorescent product. *Appl Environ Microbiol* **1995**, *61* (4), 1614-1619.
(10) Tsuchiya, S.; Yamabe, M.; Yamaguchi, Y.; Kobayashi, Y.; Konno, T.; Tada, K. Establishment and characterization of a human acute monocytic leukemia cell line (THP-1). *International Journal of Cancer* **1980**, *26* (2), 171-176.

(11) Sissoko, A.; Vásquez-Ocmín, P.; Maciuk, A.; Barbieri, D.; Neveu, G.; Rondepierre, L.; Grougnet, R.; Leproux, P.; Blaud, M.; Hammad, K.; Michel, S.; Lavazec, C.; Clain, J.; Houzé, S.; Duval, R. A Chemically Stable Fluorescent Mimic of Dihydroartemisinin, Artemether, and Arteether with Conserved Bioactivity and Specificity Shows High Pharmacological Relevance to the Antimalarial Drugs. *ACS Infect. Dis*. **2020**, *6*, 1532–1547.

SSK-32-SKM-DFUC-N3-OAC-1H

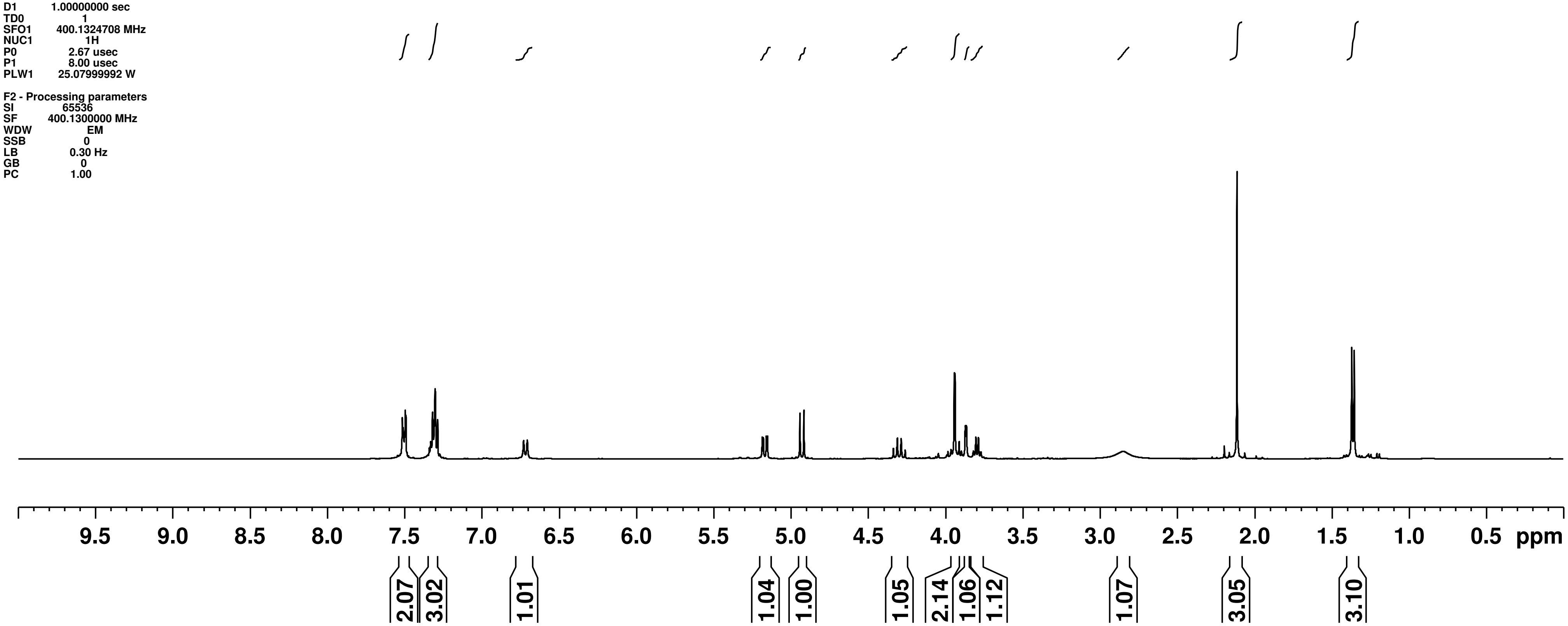

Current Data Parameters
NAME SSK-32-SKM-DFUC-N3-OAC-1H
EXPNO 10
PROCNO 1

F2 - Acquisition Parameters
Date_ 20250821
Time 23.13 h
INSTRUM Avance Neo 400
PROBHD Z163739_0226 (
PULPROG zg30
TD 51724
SOLVENT CDCl3
NS 18
DS 0
SWH 8620.690 Hz
FIDRES 0.333334 Hz
AQ 2.9999919 sec
RG 45.2
DW 58.000 usec
DE 13.14 usec
TE 296.4 K
D1 1.00000000 sec
TD0 1
SFO1 400.1324708 MHz
NUC1 1H
P0 2.67 usec
P1 8.00 usec
PLW1 25.07999992 W

F2 - Processing parameters
SI 65536
SF 400.1300000 MHz
WDW EM
SSB 0
LB 0.30 Hz
GB 0
PC 1.00

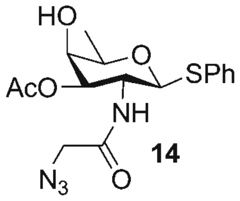

14

SSK-32-SKM-DFUC-N3-OAC-13C

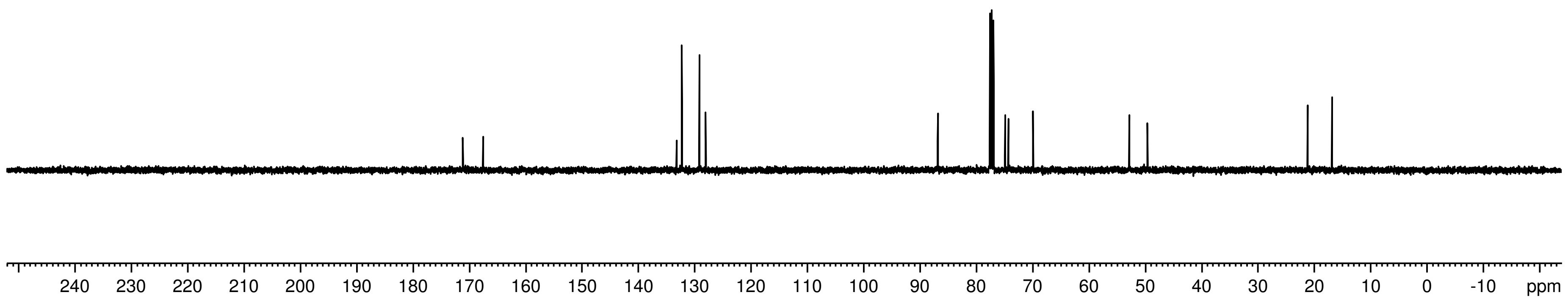

Current Data Parameters
NAME SSK-32-SKM-DFUC-N3-OAC-13C
EXPNO 12
PROCNO 1

F2 - Acquisition Parameters
Date_ 20250821
Time 23.16 h
INSTRUM Avance Neo 400
PROBHD Z163739_0226 (
PULPROG zgpg30
TD 65536
SOLVENT CDCl3
NS 64
DS 2
SWH 27777.778 Hz
FIDRES 0.847710 Hz
AQ 1.1796480 sec
RG 101
DW 18.000 usec
DE 6.50 usec
TE 297.0 K
D1 1.00000000 sec
D11 0.03000000 sec
TD0 1
SFO1 100.6242384 MHz
NUC1 13C
P0 2.67 usec
P1 8.00 usec
PLW1 97.00000000 W
SFO2 400.1316005 MHz
NUC2 1H
CPDPRG[2 waltz65
PCPD2 90.00 usec
PLW2 25.07999992 W
PLW12 0.19815999 W
PLW13 0.09967500 W

F2 - Processing parameters
SI 32768
SF 100.6127595 MHz
WDW EM
SSB 0
LB 1.00 Hz
GB 0
PC 1.40

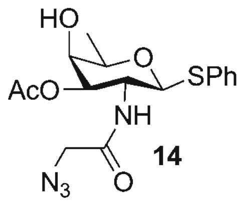

14

SSK-32-SKM-DFUC-N3-OAC-DEPT

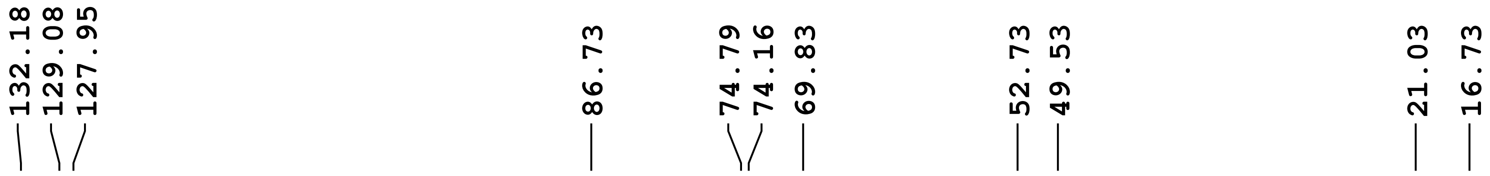

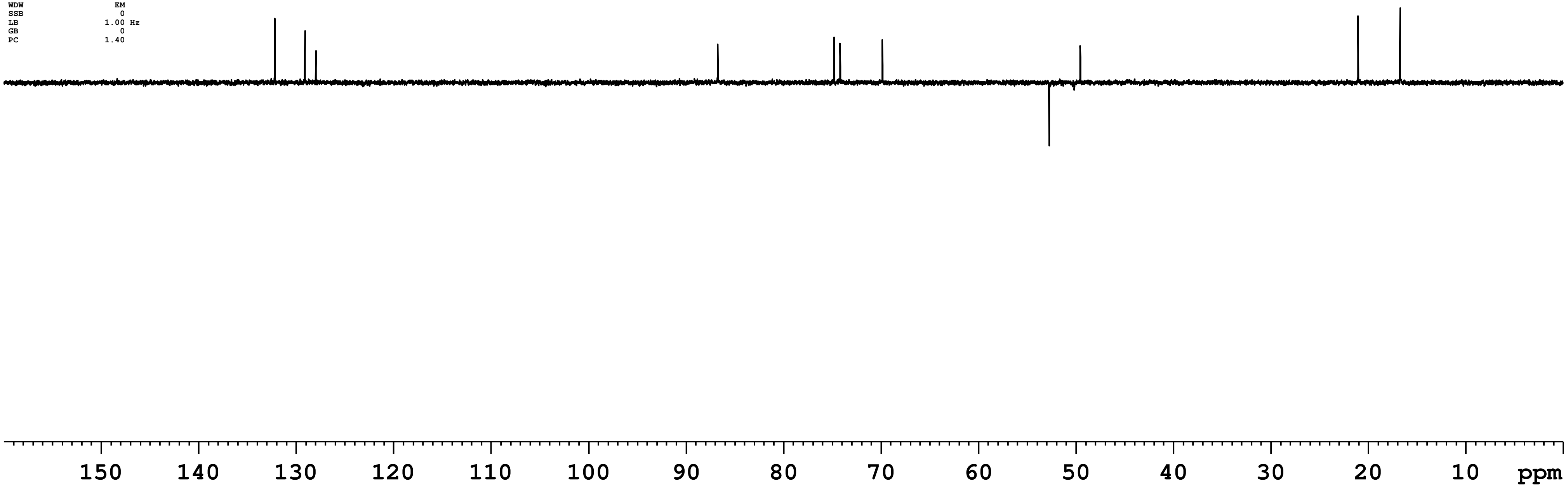


```
Current Data Parameters
NAME     SSK-32-SKM-DFUC-N3-OAC-DEPT
EXPNO                    14
PROCNO                    1

F2 - Acquisition Parameters
Date_              20250821
Time                  23.18 h
INSTRUM  Avance Neo 400
PROBHD   Z163739_0226 (
PULPROG          deptsp135
TD                    65536
SOLVENT               CDCl3
NS                       38
DS                        4
SWH               27777.778 Hz
FIDRES             0.847710 Hz
AQ                1.1796480 sec
RG                      101
DW                   18.000 usec
DE                     6.50 usec
TE                    296.9 K
CNST2           145.0000000
D1               1.00000000 sec
D2               0.00344828 sec
D12              0.00002000 sec
TD0                       1
SFO1            100.6242384 MHz
NUC1                    13C
P1                     8.00 usec
P13                 2000.00 usec
PLW0                      0 W
PLW1            97.00000000 W
SPNAM[5]        Crp60comp.4
SPOAL5                0.500
SPOFFS5                   0 Hz
SPW5             9.48509979 W
SFO2            400.1316005 MHz
NUC2                     1H
CPDPRG[2            waltz65
P3                     8.00 usec
P4                    16.00 usec
PCPD2                 90.00 usec
PLW2            25.07999992 W
PLW12            0.19815999 W

F2 - Processing parameters
SI                    32768
SF              100.6127607 MHz
WDW                      EM
SSB                       0
LB                     1.00 Hz
GB                        0
PC                     1.40
```

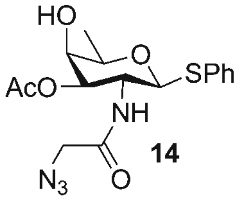

14

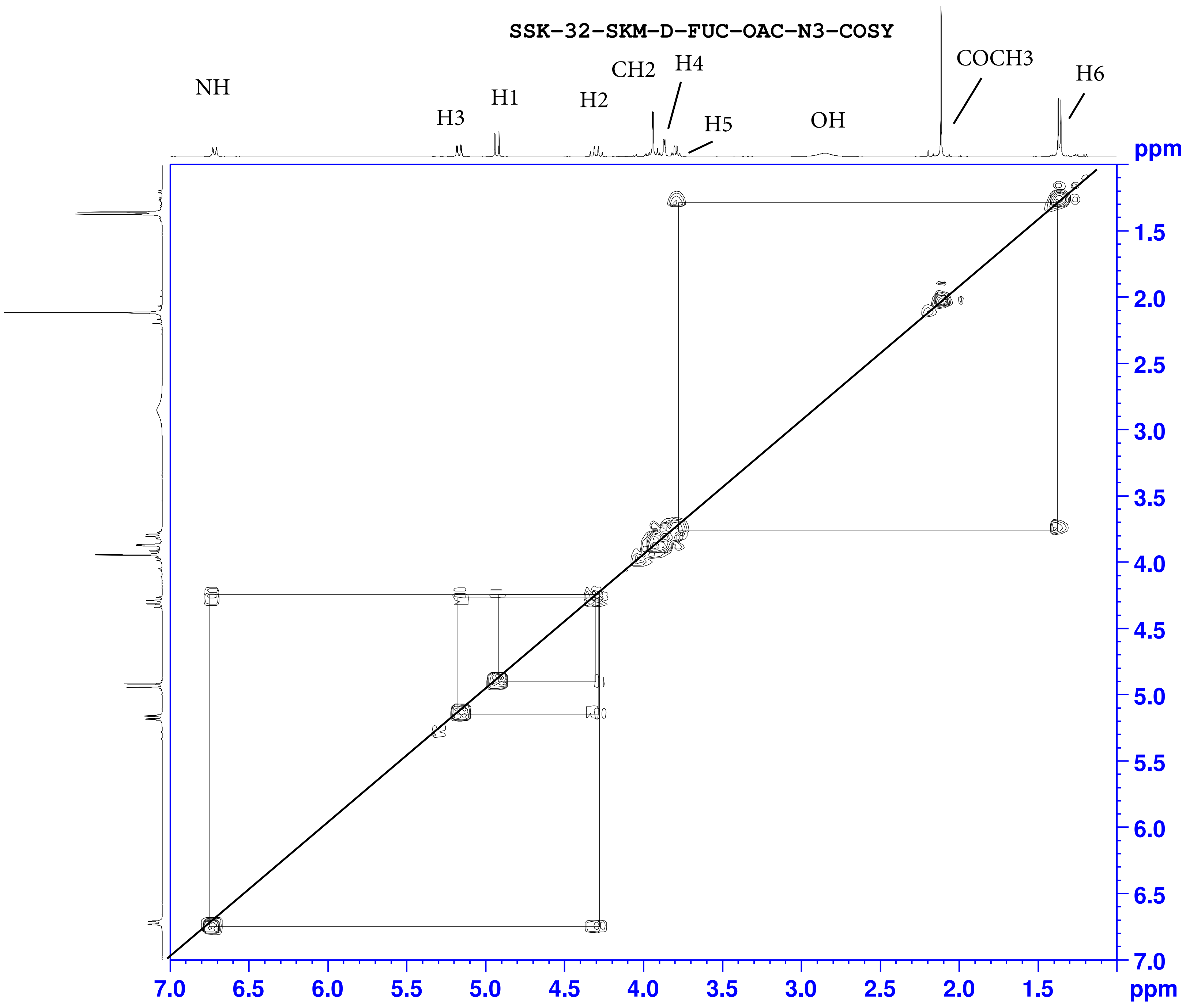

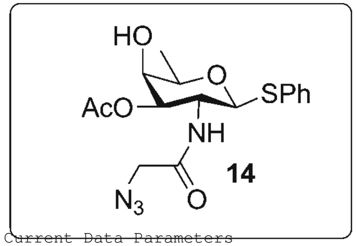

14

```
Current Data Parameters
NAME       SSK-32-SKM-D-FUC-OAC-N3-COSY
EXPNO                     1
PROCNO                    1

F2 - Acquisition Parameters
Date_              20250823
Time                  10.24 h
INSTRUM              Avance
PROBHD     Z163739_0237 (
PULPROG         cosygpppqf
TD                     2048
SOLVENT               CDCl3
NS                        4
DS                        0
SWH                8620.690 Hz
FIDRES             8.418642 Hz
AQ                0.1187840 sec
RG                      101
DW                   58.000 usec
DE                     6.50 usec
TE                    297.1 K
D0               0.00000300 sec
D1               1.00000000 sec
D11              0.03000000 sec
D12              0.00002000 sec
D13              0.00000400 sec
D16              0.00020000 sec
IN0              0.00011355 sec
TDav                      1
SFO1            400.3024018 MHz
NUC1                     1H
P0                     8.00 usec
P1                     8.00 usec
P17                 2500.00 usec
PLW1            21.00099945 W
PLW10            1.49339998 W
GPNAM[1]           SMSQ10.100
GPZ1                  10.00 %
P16                 1000.00 usec

======== F1 INDIRECT DIMENSION ========
td1        128
sw_F1      22.000000

F1 - Acquisition parameters
TD                      128
SFO1               400.3024 MHz
FIDRES           137.603958 Hz
SW                   22.000 ppm
FnMODE                   QF

F2 - Processing parameters
SI                     1024
SF              400.3000000 MHz
WDW                   QSINE
SSB                       0
LB                        0 Hz
GB                        0
PC                     1.40

F1 - Processing parameters
SI                     1024
MC2                      QF
SF              400.3000000 MHz
WDW                   QSINE
SSB                       0
LB                        0 Hz
GB                        0
```

SSK-32-SKM-D-FUC-OAC-N3-HMBC

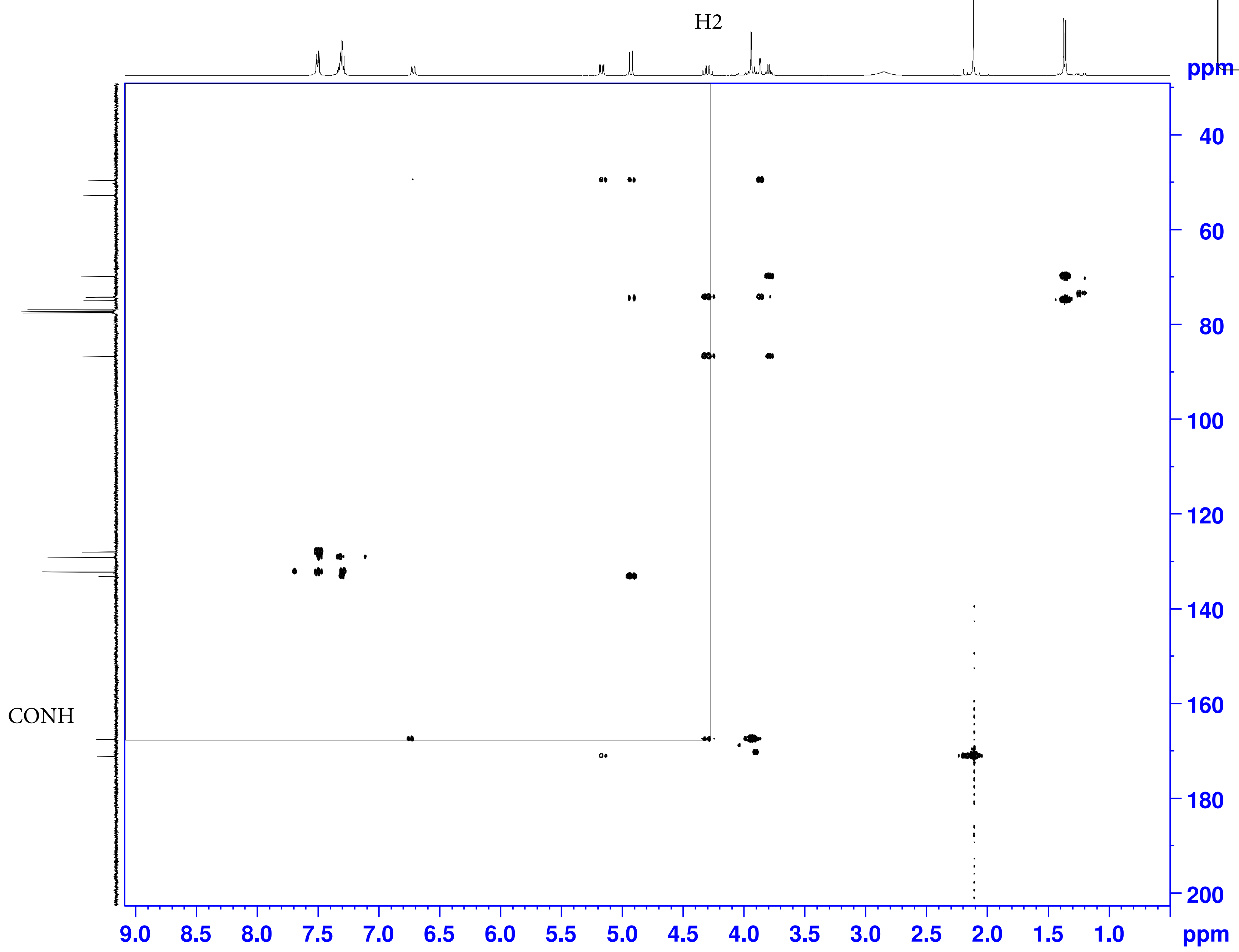

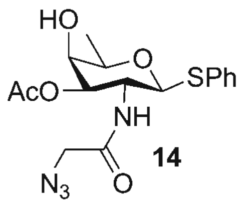

14

Current Data Parameters
NAME SSK-32-SKM-D-FUC-OAC-N3-HMBC
EXPNO 3
PROCNO 1

F2 - Acquisition Parameters
Date_ 20250823
Time 11.51 h
INSTRUM Avance
PROBHD Z163739_0237 (
PULPROG hmbcetgpl3nd
TD 2048
SOLVENT CDCl3
NS 2
DS 0
SWH 8196.721 Hz
FIDRES 8.004611 Hz
AQ 0.1249280 sec
RG 101
DW 61.000 usec
DE 6.50 usec
TE 297.5 K
CNST6 120.0000000
CNST7 170.0000000
CNST13 8.0000000
CNST30 0.5981113
D0 0.00000300 sec
D1 1.00000000 sec
D6 0.06250000 sec
D16 0.00020000 sec
IN0 0.00001799 sec
TDav 1
SFO1 400.3024719 MHz
NUC1 1H
P1 8.00 usec
P2 16.00 usec
PLW1 21.00099945 W
SFO2 100.6669898 MHz
NUC2 13C
P3 8.00 usec
P24 2000.00 usec
PLW2 97.90799713 W
SPNAM[7] Crp60comp.4
SPOAL7 0.500
SPOFFS7 0 Hz
SPW7 9.57390022 W
GPNAM[1] SMSQ10.100
GPZ1 80.00 %
GPNAM[3] SMSQ10.100
GPZ3 14.00 %
GPNAM[4] SMSQ10.100
GPZ4 -8.00 %
GPNAM[5] SMSQ10.100
GPZ5 -4.00 %
GPNAM[6] SMSQ10.100
GPZ6 -2.00 %
P16 1000.00 usec

======== F1 INDIRECT DIMENSION ========
td1 2048
sw_F1 275.996124

F1 - Acquisition parameters
TD 372
SFO1 100.667 MHz
FIDRES 149.374725 Hz
SW 275.996 ppm
FnMODE Echo-Antiecho

F2 - Processing parameters
SI 2048
SF 400.3000000 MHz
WDW SINE
SSB 4
LB 0 Hz
GB 0
PC 1.40

F1 - Processing parameters
SI 1024
MC2 echo-antiecho
SF 100.6555151 MHz
WDW QSINE
SSB 2
LB 0 Hz
GB 0

SSK-32-SKM-DFUC-N3-OAC-HSQC

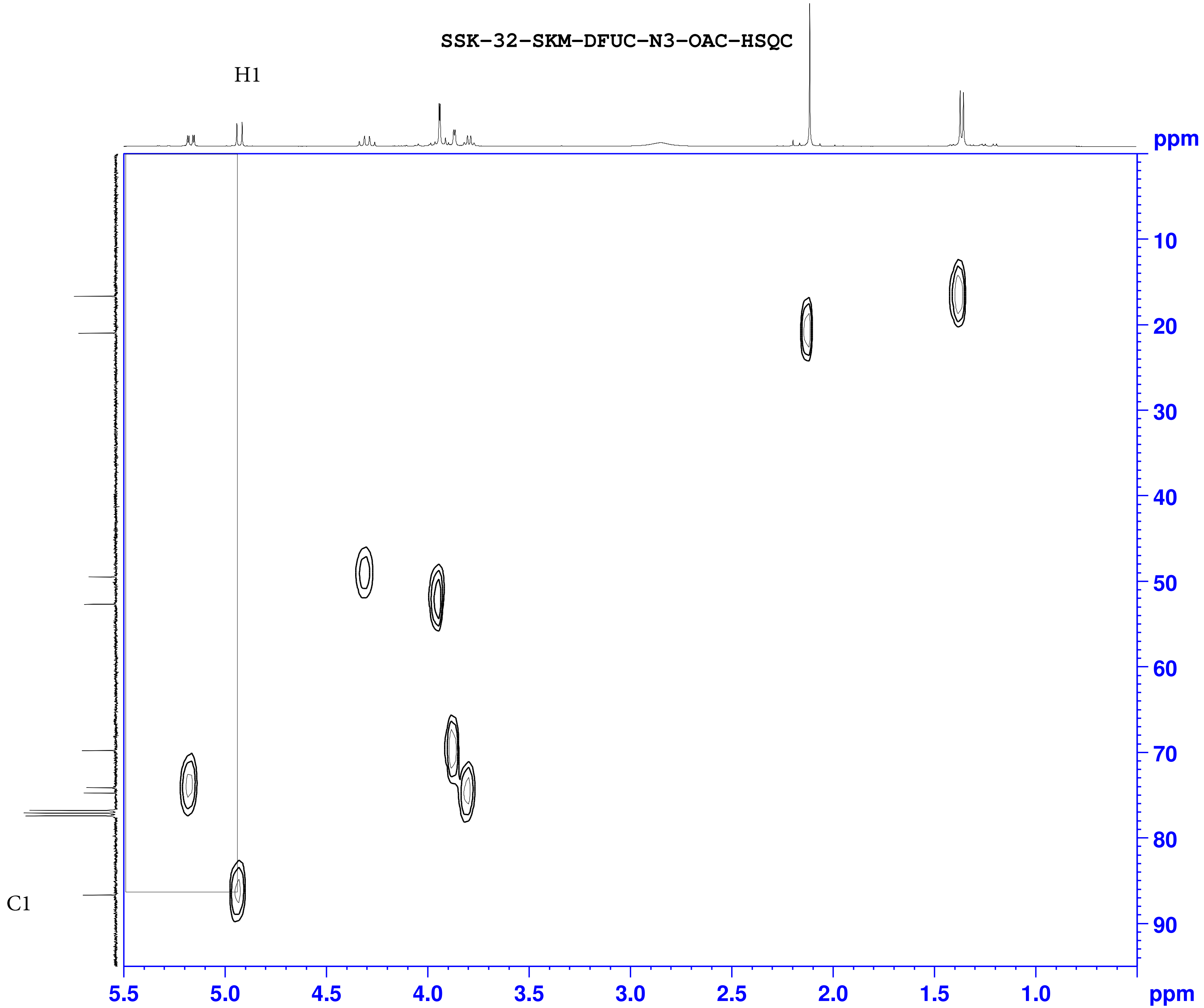

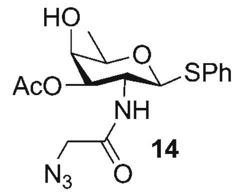

14

```
Current Data Parameters
NAME      SSK-32-SKM-DFUC-N3-OAC-HSQC
EXPNO                    18
PROCNO                    1

F2 - Acquisition Parameters
Date_              20250821
Time                  23.30 h
INSTRUM  Avance Neo 400
PROBHD   Z163739_0226 (
PULPROG  hsqcedetgpsisp2.3
TD                     2048
SOLVENT               CDCl3
NS                        2
DS                        4
SWH                8620.690 Hz
FIDRES             8.418642 Hz
AQ                0.1187840 sec
RG                      101
DW                   58.000 usec
DE                     6.50 usec
TE                    296.4 K
CNST2           145.0000000
CNST17           -0.5000000
D0               0.00000300 sec
D1               1.50000000 sec
D4               0.00172414 sec
D11              0.03000000 sec
D16              0.00020000 sec
D21              0.00344800 sec
D24              0.00086200 sec
IN0              0.00001801 sec
TDav                      1
ZGOPTNS
SFO1            400.1324708 MHz
NUC1                     1H
P1                     8.00 usec
P2                    16.00 usec
PLW1            25.07999992 W
SFO2            100.6242384 MHz
NUC2                    13C
CPDPRG[2              garp4
P3                     8.00 usec
P14                  500.00 usec
P24                 2000.00 usec
P31                 2119.00 usec
PCPD2                 80.00 usec
PLW0                      0 W
PLW2            97.00000000 W
PLW12            0.97000003 W
SPNAM[3] Crp60,0.5,20.1
SPOAL3                0.500
SPOFFS3                   0 Hz
SPW3             9.48509979 W
SPNAM[7]    Crp60comp.4
SPOAL7                0.500
SPOFFS7                   0 Hz
SPW7             9.48509979 W
SPNAM[18  Crp60_xfilt.2
SPOAL18               0.500
SPOFFS18                  0 Hz
SPW18            1.82720006 W
GPNAM[1]     SMSQ10.100
GPZ1                  80.00 %
GPNAM[2]     SMSQ10.100
GPZ2                  20.10 %
GPNAM[3]     SMSQ10.100
GPZ3                  11.00 %
GPNAM[4]     SMSQ10.100
GPZ4                  -5.00 %
P16                 1000.00 usec
P19                  600.00 usec

======== F1 INDIRECT DIMENSION ========
td1      200
sw_F1    275.989227

F1 - Acquisition parameters
TD                       52
SFO1               100.6242 MHz
FIDRES          1068.123291 Hz
SW                  275.989 ppm
FnMODE    Echo-Antiecho

F2 - Processing parameters
SI                     1024
SF              400.1300000 MHz
WDW                   QSINE
SSB                       2
LB                        0 Hz
GB                        0
PC                     1.40

F1 - Processing parameters
SI                     1024
MC2           echo-antiecho
SF              100.6127685 MHz
WDW                   QSINE
SSB                       2
LB                        0 Hz
GB                        0
```

SSK-32-SKM-DFUC-FI-1H

Current Data Parameters
NAME SSK-32-SKM-DFUC-FI-1H
EXPNO 20
PROCNO 1

F2 - Acquisition Parameters
Date_ 20250822
Time 22.57 h
INSTRUM Avance Neo 400
PROBHD Z163739_0226 (
PULPROG zg30
TD 51724
SOLVENT MeOD
NS 18
DS 0
SWH 8620.690 Hz
FIDRES 0.333334 Hz
AQ 2.9999919 sec
RG 101
DW 58.000 usec
DE 13.14 usec
TE 296.7 K
D1 1.00000000 sec
TD0 1
SFO1 400.1324708 MHz
NUC1 1H
P0 2.67 usec
P1 8.00 usec
PLW1 25.07999992 W

F2 - Processing parameters
SI 65536
SF 400.1300000 MHz
WDW EM
SSB 0
LB 0.30 Hz
GB 0
PC 1.00

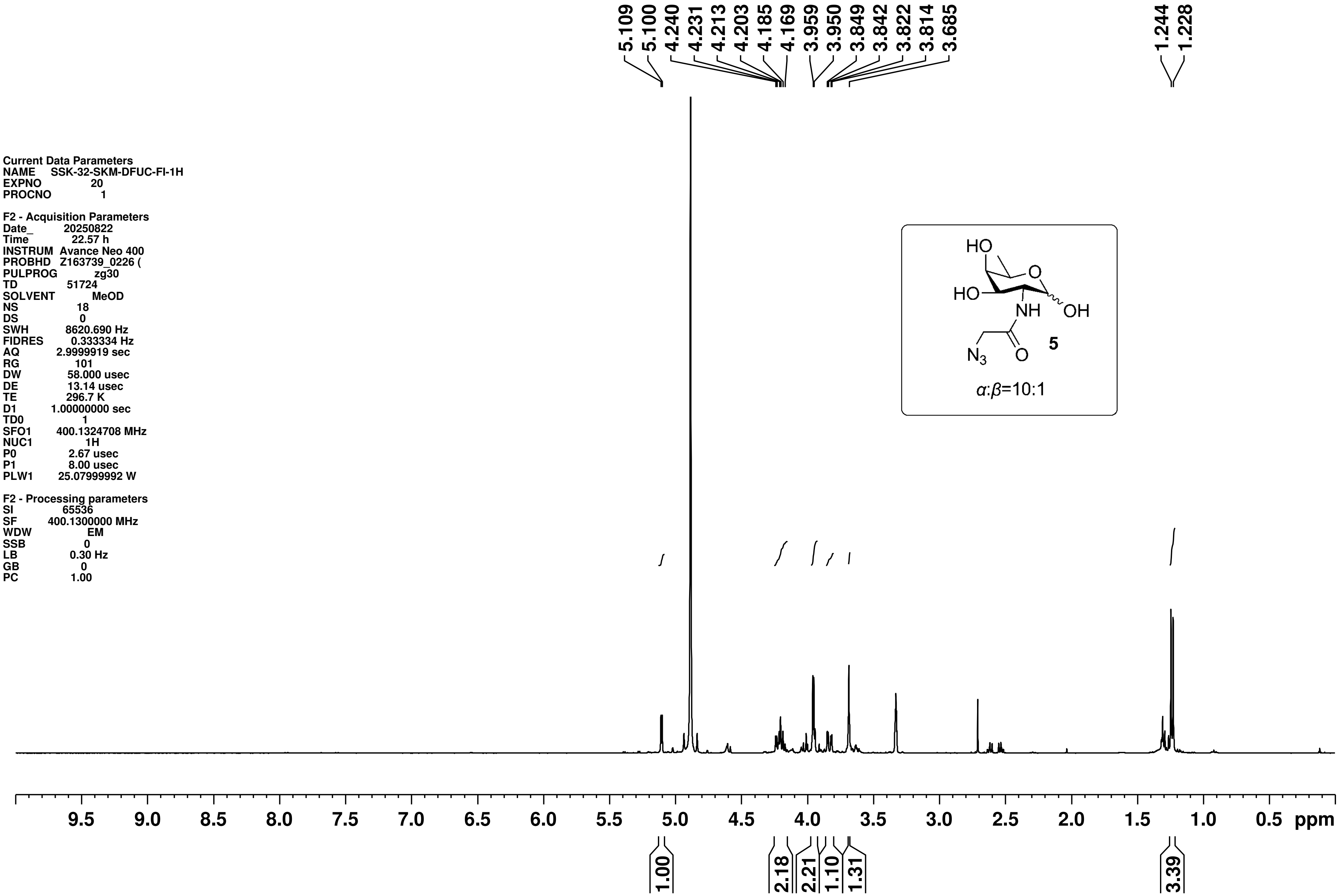

SSK-32-SKM-DFUC-FI-13C

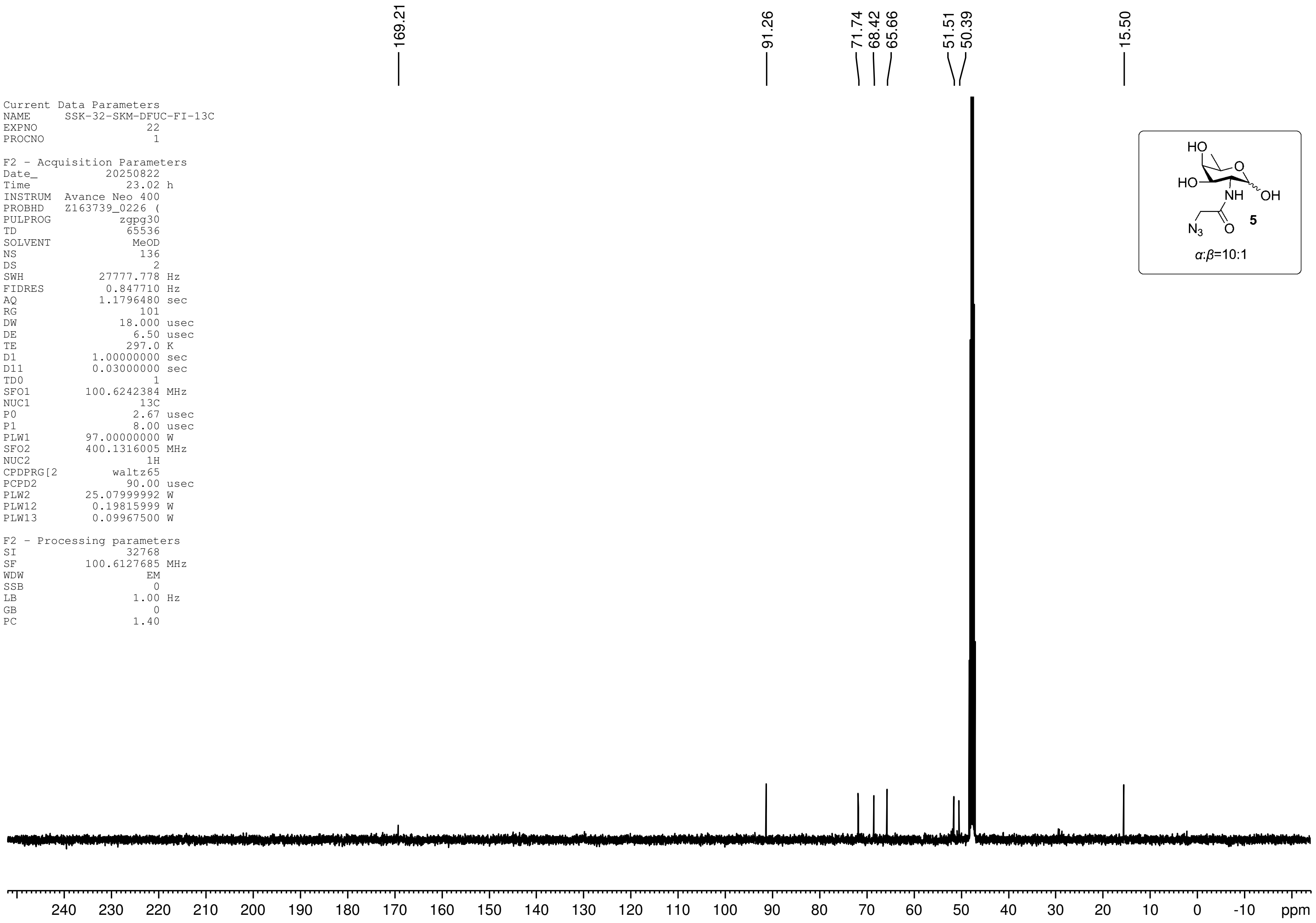


```
Current Data Parameters
NAME      SSK-32-SKM-DFUC-FI-13C
EXPNO                 22
PROCNO                 1

F2 - Acquisition Parameters
Date_           20250822
Time               23.02 h
INSTRUM  Avance Neo 400
PROBHD   Z163739_0226 (
PULPROG           zgpg30
TD                 65536
SOLVENT             MeOD
NS                   136
DS                     2
SWH          27777.778 Hz
FIDRES        0.847710 Hz
AQ           1.1796480 sec
RG                   101
DW                18.000 usec
DE                  6.50 usec
TE                 297.0 K
D1            1.00000000 sec
D11           0.03000000 sec
TD0                    1
SFO1        100.6242384 MHz
NUC1                 13C
P0                  2.67 usec
P1                  8.00 usec
PLW1         97.00000000 W
SFO2        400.1316005 MHz
NUC2                  1H
CPDPRG[2         waltz65
PCPD2              90.00 usec
PLW2         25.07999992 W
PLW12         0.19815999 W
PLW13         0.09967500 W

F2 - Processing parameters
SI                 32768
SF          100.6127685 MHz
WDW                   EM
SSB                    0
LB                  1.00 Hz
GB                     0
PC                  1.40
```

5

α:β=10:1

SSK-32-SKM-DFUC-FI-DEPT

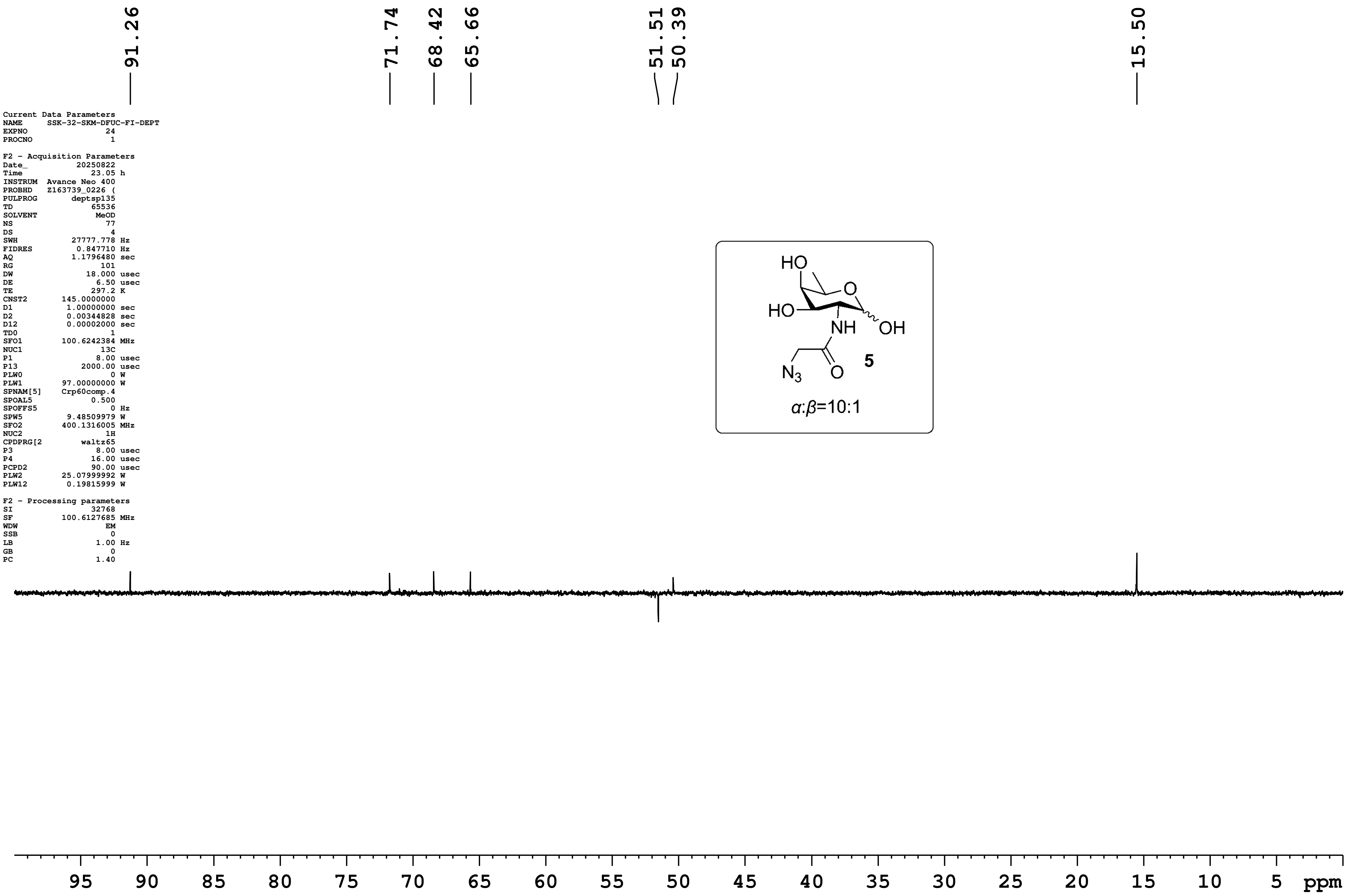


```
Current Data Parameters
NAME     SSK-32-SKM-DFUC-FI-DEPT
EXPNO                 24
PROCNO                 1

F2 - Acquisition Parameters
Date_           20250822
Time               23.05 h
INSTRUM  Avance Neo 400
PROBHD   Z163739_0226 (
PULPROG       deptsp135
TD                 65536
SOLVENT             MeOD
NS                    77
DS                     4
SWH            27777.778 Hz
FIDRES          0.847710 Hz
AQ             1.1796480 sec
RG                   101
DW                18.000 usec
DE                  6.50 usec
TE                 297.2 K
CNST2        145.0000000
D1            1.00000000 sec
D2            0.00344828 sec
D12           0.00002000 sec
TD0                    1
SFO1         100.6242384 MHz
NUC1                 13C
P1                  8.00 usec
P13              2000.00 usec
PLW0                   0 W
PLW1         97.00000000 W
SPNAM[5]     Crp60comp.4
SPOAL5             0.500
SPOFFS5                0 Hz
SPW5          9.48509979 W
SFO2         400.1316005 MHz
NUC2                  1H
CPDPRG[2         waltz65
P3                  8.00 usec
P4                 16.00 usec
PCPD2              90.00 usec
PLW2         25.07999992 W
PLW12         0.19815999 W

F2 - Processing parameters
SI                 32768
SF           100.6127685 MHz
WDW                   EM
SSB                    0
LB                  1.00 Hz
GB                     0
PC                  1.40
```

5

α:β=10:1

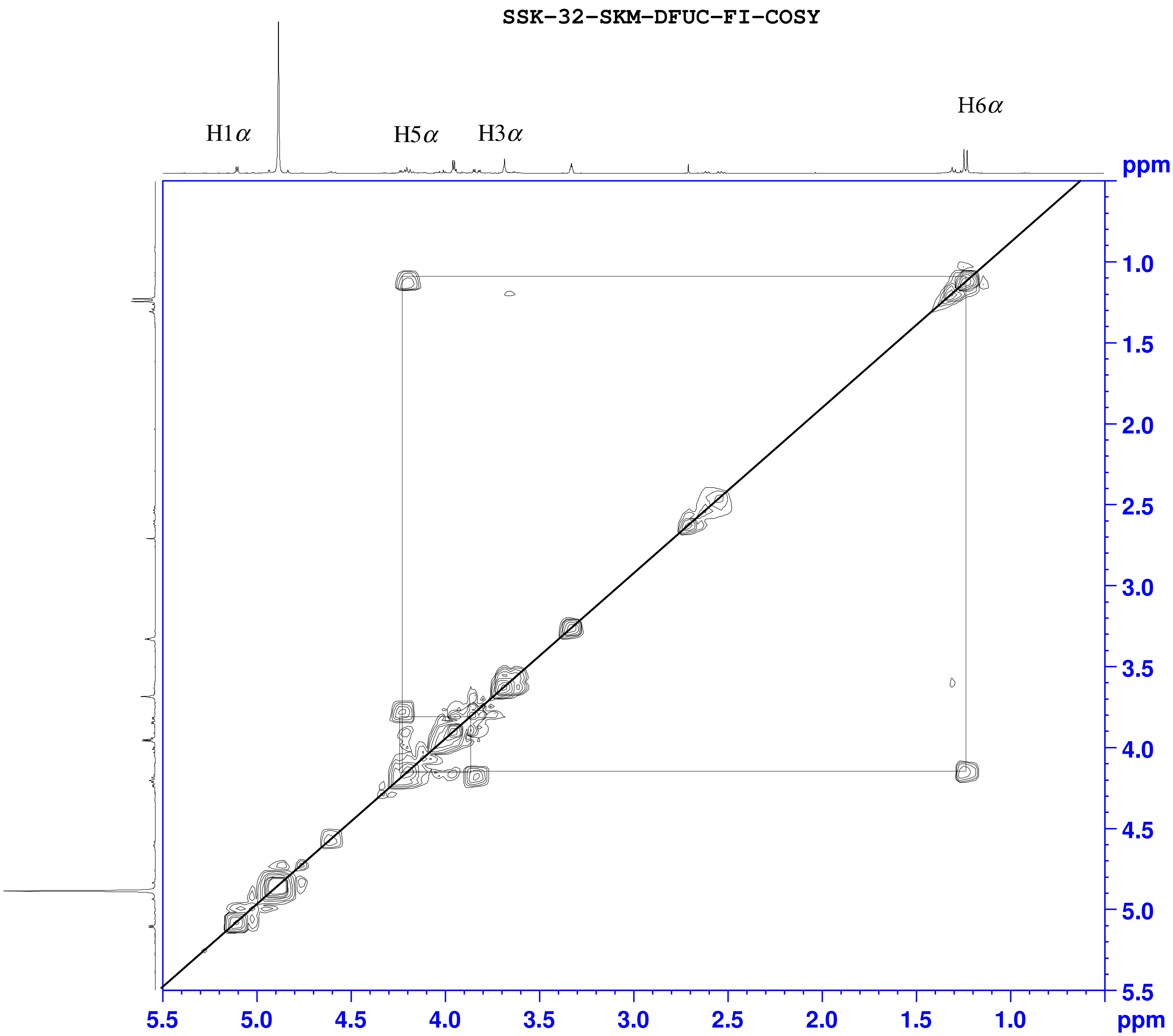

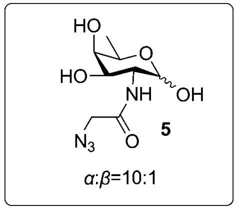


```
Current Data Parameters
NAME      SSK-32-SKM-DFUC-FI-COSY
EXPNO                  26
PROCNO                  1

F2 - Acquisition Parameters
Date_          20250822
Time              23.24 h
INSTRUM   Avance Neo 400
PROBHD    Z163739_0226 (
PULPROG        cosygpppqf
TD                  2048
SOLVENT             MeOD
NS                     4
DS                     4
SWH             8620.690 Hz
FIDRES          8.418642 Hz
AQ             0.1187840 sec
RG                   101
DW                58.000 usec
DE                  6.50 usec
TE                 297.0 K
D0            0.00000300 sec
D1            2.00000000 sec
D11           0.03000000 sec
D12           0.00002000 sec
D13           0.00000400 sec
D16           0.00020000 sec
IN0           0.00011360 sec
TDav                   1
SFO1         400.1324708 MHz
NUC1                  1H
P0                  8.00 usec
P1                  8.00 usec
P17              2500.00 usec
PLW1         25.07999992 W
PLW10         1.78349996 W
GPNAM[1]      SMSQ10.100
GPZ1               10.00 %
P16              1000.00 usec

======== F1 INDIRECT DIMENSION ========
td1       128
sw_F1     21.999996

F1 - Acquisition parameters
TD                   128
SFO1            400.1325 MHz
FIDRES        137.545517 Hz
SW                22.000 ppm
FnMODE                QF

F2 - Processing parameters
SI                  1024
SF           400.1300000 MHz
WDW                QSINE
SSB                    0
LB                     0 Hz
GB                     0
PC                  1.40

F1 - Processing parameters
SI                  1024
MC2                   QF
SF           400.1300000 MHz
WDW                QSINE
SSB                    0
LB                     0 Hz
GB                     0
```

SSK-32-SKM-DFUC-FI-HSQC

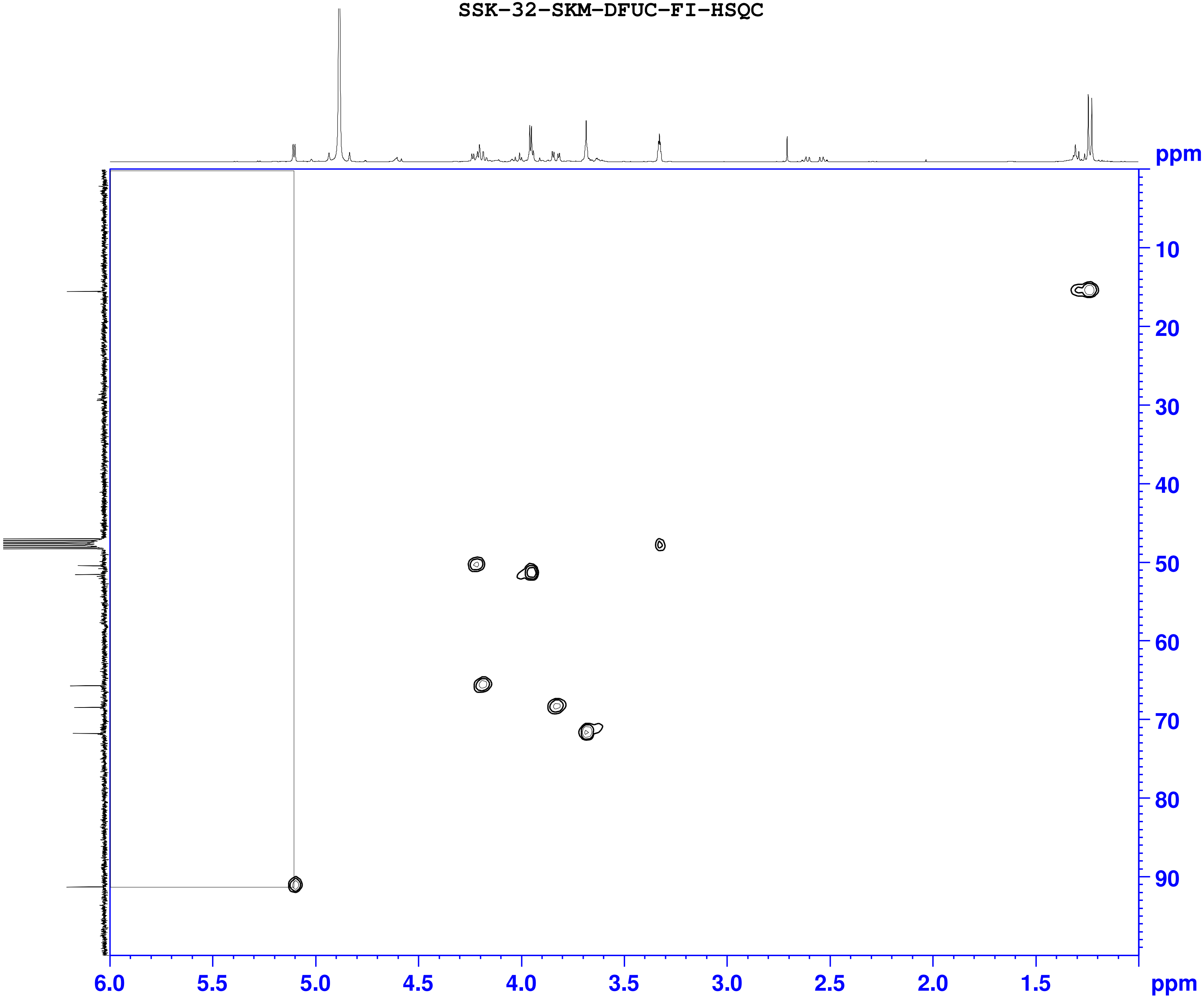

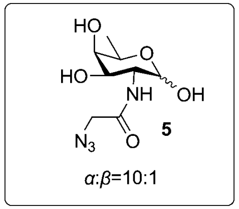


```
Current Data Parameters
NAME     SSK-32-SKM-DFUC-FI-HSQC
EXPNO                 28
PROCNO                 1

F2 - Acquisition Parameters
Date_           20250822
Time               23.35 h
INSTRUM  Avance Neo 400
PROBHD   Z163739_0226 (
PULPROG  hsqcedetgpsisp2.3
TD                  2048
SOLVENT             MeOD
NS                     2
DS                     4
SWH             8620.690 Hz
FIDRES          8.418642 Hz
AQ             0.1187840 sec
RG                   101
DW                58.000 usec
DE                  6.50 usec
TE                 296.5 K
CNST2        145.0000000
CNST17        -0.5000000
D0            0.00000300 sec
D1            1.50000000 sec
D4            0.00172414 sec
D11           0.03000000 sec
D16           0.00020000 sec
D21           0.00344800 sec
D24           0.00086200 sec
IN0           0.00001801 sec
TDav                   1
ZGOPTNS
SFO1         400.1324708 MHz
NUC1                  1H
P1                  8.00 usec
P2                 16.00 usec
PLW1         25.07999992 W
SFO2         100.6242384 MHz
NUC2                 13C
CPDPRG[2           garp4
P3                  8.00 usec
P14               500.00 usec
P24              2000.00 usec
P31              2119.00 usec
PCPD2              80.00 usec
PLW0                   0 W
PLW2         97.00000000 W
PLW12         0.97000003 W
SPNAM[3] Crp60,0.5,20.1
SPOAL3             0.500
SPOFFS3                0 Hz
SPW3          9.48509979 W
SPNAM[7]    Crp60comp.4
SPOAL7             0.500
SPOFFS7                0 Hz
SPW7          9.48509979 W
SPNAM[18  Crp60_xfilt.2
SPOAL18            0.500
SPOFFS18               0 Hz
SPW18         1.82720006 W
GPNAM[1]     SMSQ10.100
GPZ1               80.00 %
GPNAM[2]     SMSQ10.100
GPZ2               20.10 %
GPNAM[3]     SMSQ10.100
GPZ3               11.00 %
GPNAM[4]     SMSQ10.100
GPZ4               -5.00 %
P16              1000.00 usec
P19               600.00 usec

======== F1 INDIRECT DIMENSION ========
td1      200
sw_F1    275.989227

F1 - Acquisition parameters
TD                   200
SFO1            100.6242 MHz
FIDRES        277.712067 Hz
SW               275.989 ppm
FnMODE    Echo-Antiecho

F2 - Processing parameters
SI                  1024
SF           400.1300000 MHz
WDW                QSINE
SSB                    2
LB                     0 Hz
GB                     0
PC                  1.40

F1 - Processing parameters
SI                  1024
MC2       echo-antiecho
SF           100.6127685 MHz
WDW                QSINE
SSB                    2
LB                     0 Hz
GB                     0
```

SSK-32-SKM-LPNE-AZA-FI-1H

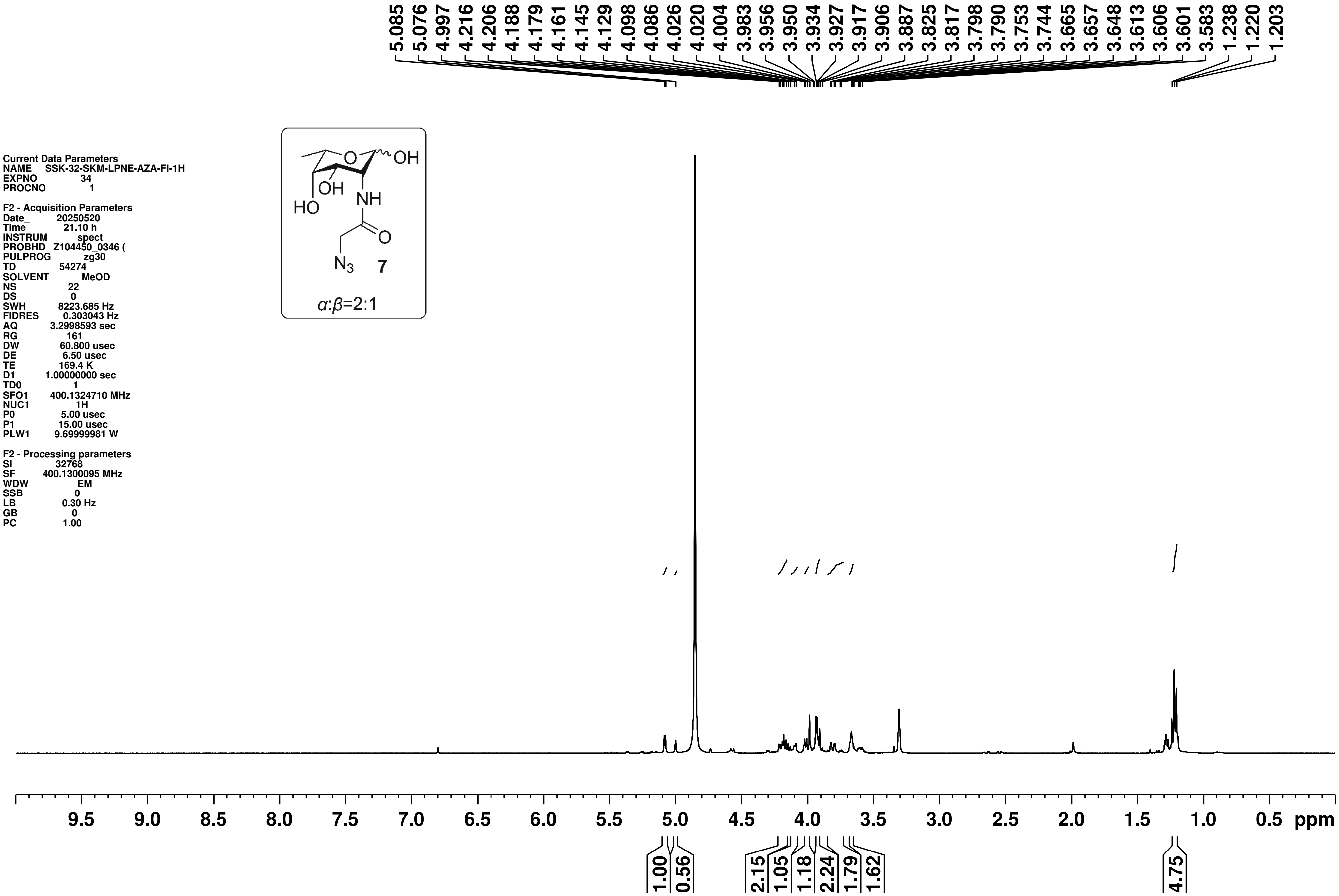

Current Data Parameters
NAME SSK-32-SKM-LPNE-AZA-FI-1H
EXPNO 34
PROCNO 1

F2 - Acquisition Parameters
Date_ 20250520
Time 21.10 h
INSTRUM spect
PROBHD Z104450_0346 (
PULPROG zg30
TD 54274
SOLVENT MeOD
NS 22
DS 0
SWH 8223.685 Hz
FIDRES 0.303043 Hz
AQ 3.2998593 sec
RG 161
DW 60.800 usec
DE 6.50 usec
TE 169.4 K
D1 1.00000000 sec
TD0 1
SFO1 400.1324710 MHz
NUC1 1H
P0 5.00 usec
P1 15.00 usec
PLW1 9.69999981 W

F2 - Processing parameters
SI 32768
SF 400.1300095 MHz
WDW EM
SSB 0
LB 0.30 Hz
GB 0
PC 1.00

7

α:β=2:1

SSK-32-SKM-LPNE-AZA-FI-13C

Current Data Parameters
NAME SSK-32-SKM-LPNE-AZA-FI-13C
EXPNO 33
PROCNO 1

F2 - Acquisition Parameters
Date_ 20250520
Time 21.34 h
INSTRUM spect
PROBHD Z104450_0346 (
PULPROG zgpg30
TD 65536
SOLVENT MeOD
NS 600
DS 0
SWH 26041.666 Hz
FIDRES 0.794729 Hz
AQ 1.2582912 sec
RG 1030
DW 19.200 usec
DE 6.50 usec
TE 169.4 K
D1 1.00000000 sec
D11 0.03000000 sec
TD0 1
SFO1 100.6238364 MHz
NUC1 13C
P0 3.33 usec
P1 10.00 usec
PLW1 47.00000000 W
SFO2 400.1316005 MHz
NUC2 1H
CPDPRG[2 waltz16
PCPD2 90.00 usec
PLW2 9.69999981 W
PLW12 0.26944000 W
PLW13 0.13552999 W

F2 - Processing parameters
SI 32768
SF 100.6127690 MHz
WDW EM
SSB 0
LB 1.00 Hz
GB 0
PC 1.40

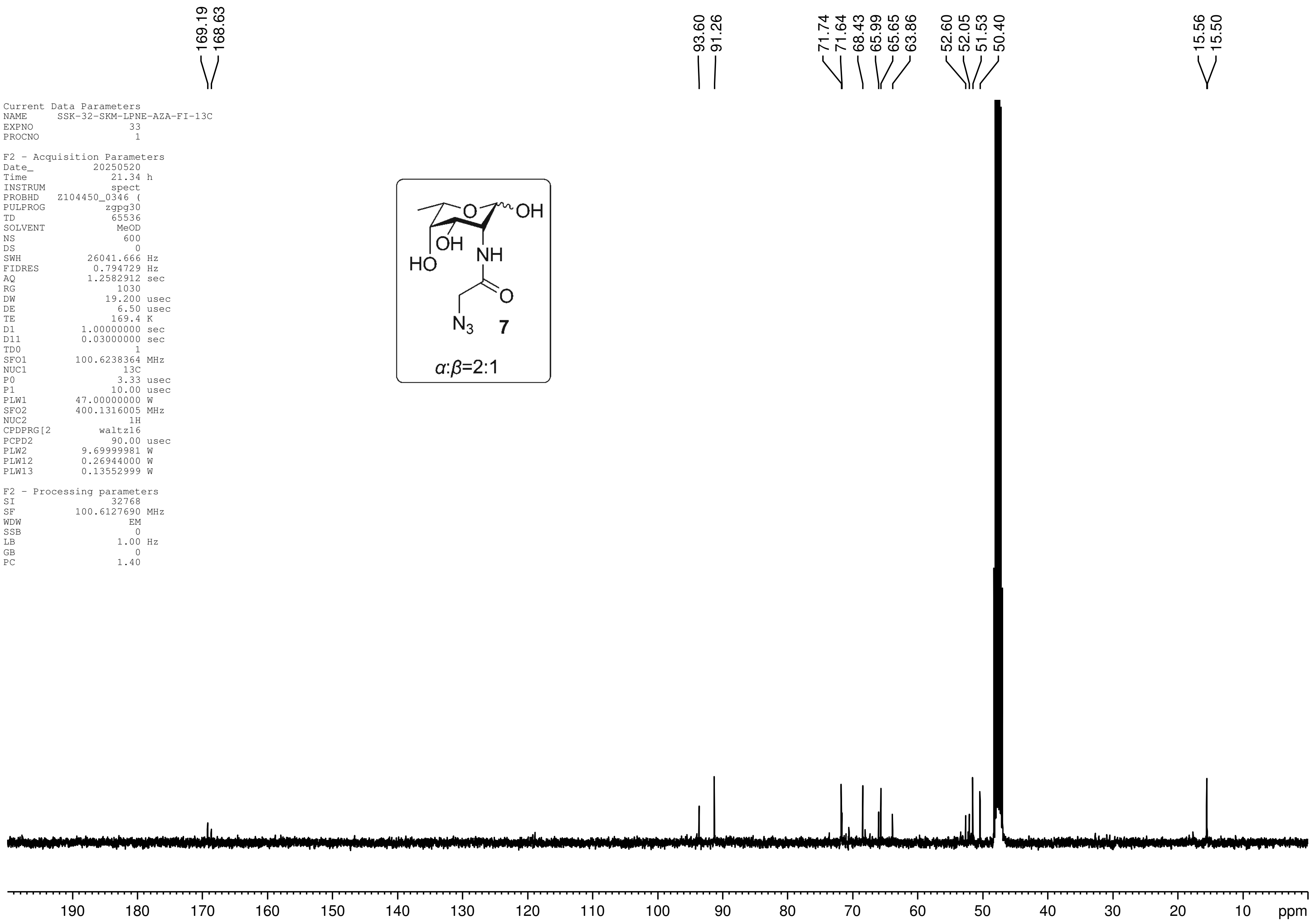

SSK-32-SKM-LPNE-AZA-FI-DEPT

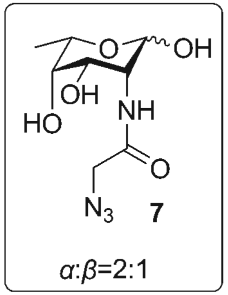

7

Current Data Parameters	
NAME	SSK-32-SKM-LPNE-AZA-FI-DEPT
EXPNO	34
PROCNO	1

F2 - Acquisition Parameters	
Date_	20250520
Time	21.52 h
INSTRUM	spect
PROBHD	Z104450_0346 (
PULPROG	dept135
TD	65536
SOLVENT	MeOD
NS	500
DS	0
SWH	27777.777 Hz
FIDRES	0.847710 Hz
AQ	1.1796480 sec
RG	203
DW	18.000 usec
DE	6.50 usec
TE	168.9 K
CNST2	145.0000000
D1	1.00000000 sec
D2	0.00344828 sec
D12	0.00002000 sec
TD0	1
SFO1	100.6242389 MHz
NUC1	13C
P1	10.00 usec
P2	20.00 usec
PLW1	47.00000000 W
SFO2	400.1316005 MHz
NUC2	1H
CPDPRG[2	waltz16
P3	15.00 usec
P4	30.00 usec
PCPD2	90.00 usec
PLW2	9.69999981 W
PLW12	0.26944000 W

F2 - Processing parameters	
SI	32768
SF	100.6127690 MHz
WDW	EM
SSB	0
LB	1.00 Hz
GB	0
PC	1.40

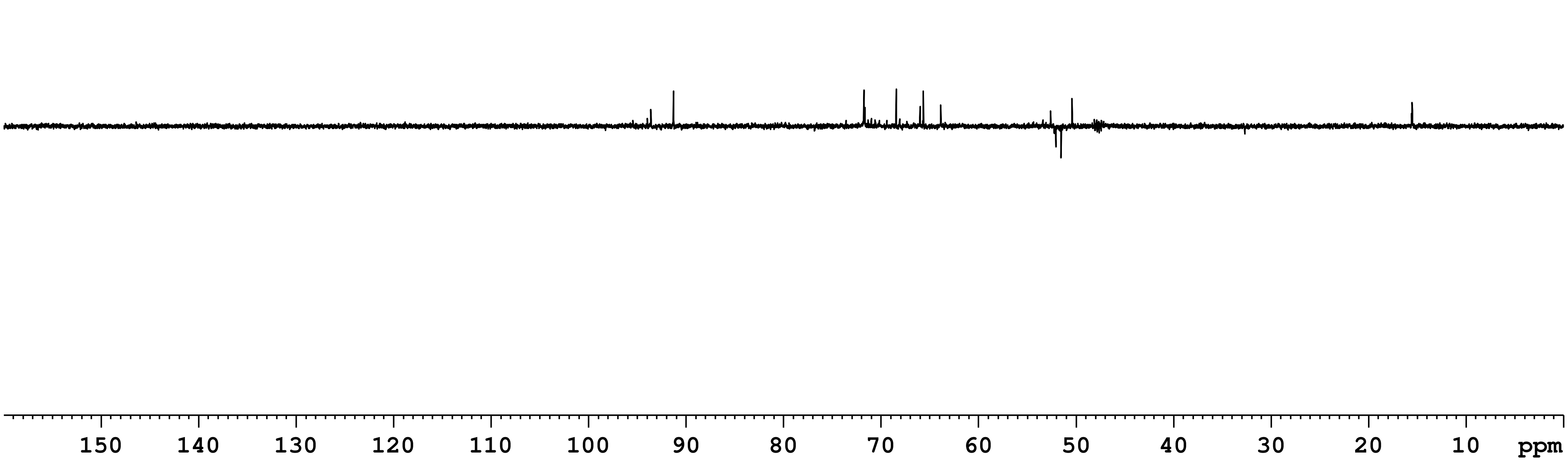

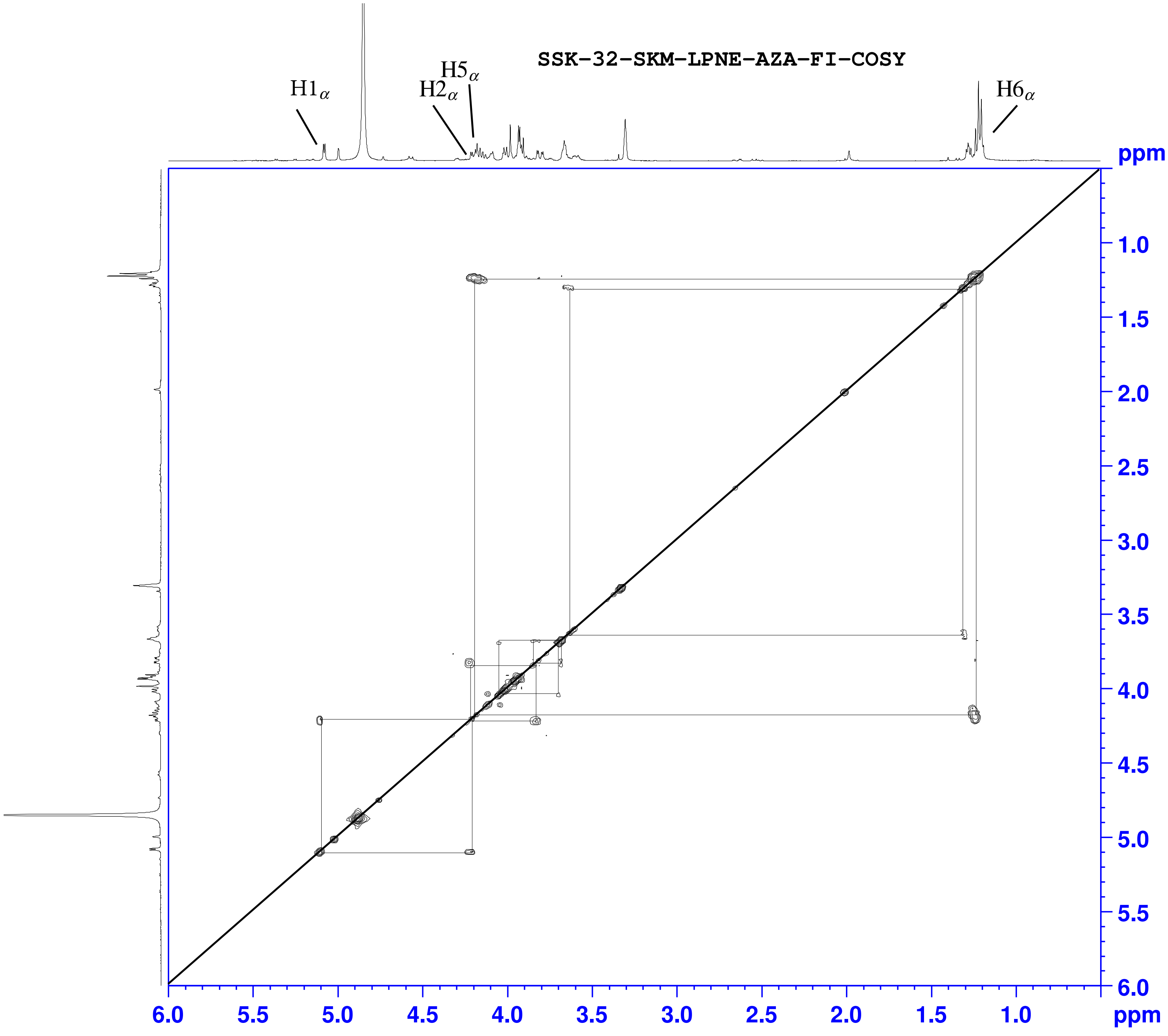

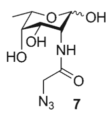

7

α:β=2:1

Current Data Parameters
NAME SSK-32-SKM-LPNE-AZA-FI-COSY
EXPNO 34
PROCNO 1

F2 - Acquisition Parameters
Date_ 20250520
Time 22.38 h
INSTRUM spect
PROBHD Z104450_0346 (
PULPROG cosygpqf
TD 2048
SOLVENT MeOD
NS 4
DS 0
SWH 5341.880 Hz
FIDRES 5.216680 Hz
AQ 0.1916928 sec
RG 64
DW 93.600 usec
DE 6.50 usec
TE 168.5 K
D0 0.00000300 sec
D1 1.00000000 sec
D13 0.00000400 sec
D16 0.00020000 sec
IN0 0.00018720 sec
TDav 1
SFO1 400.1324057 MHz
NUC1 1H
P0 15.00 usec
P1 15.00 usec
PLW1 9.69999981 W
GPNAM[1] SINE.100
GPZ1 10.00 %
P16 1000.00 usec

F1 - Acquisition parameters
TD 364
SFO1 400.1324 MHz
FIDRES 29.350990 Hz
SW 13.350 ppm
FnMODE QF

F2 - Processing parameters
SI 1024
SF 400.1300000 MHz
WDW SINE
SSB 0
LB 0 Hz
GB 0
PC 1.40

F1 - Processing parameters
SI 1024
MC2 QF
SF 400.1300000 MHz
WDW SINE
SSB 0
LB 0 Hz
GB 0

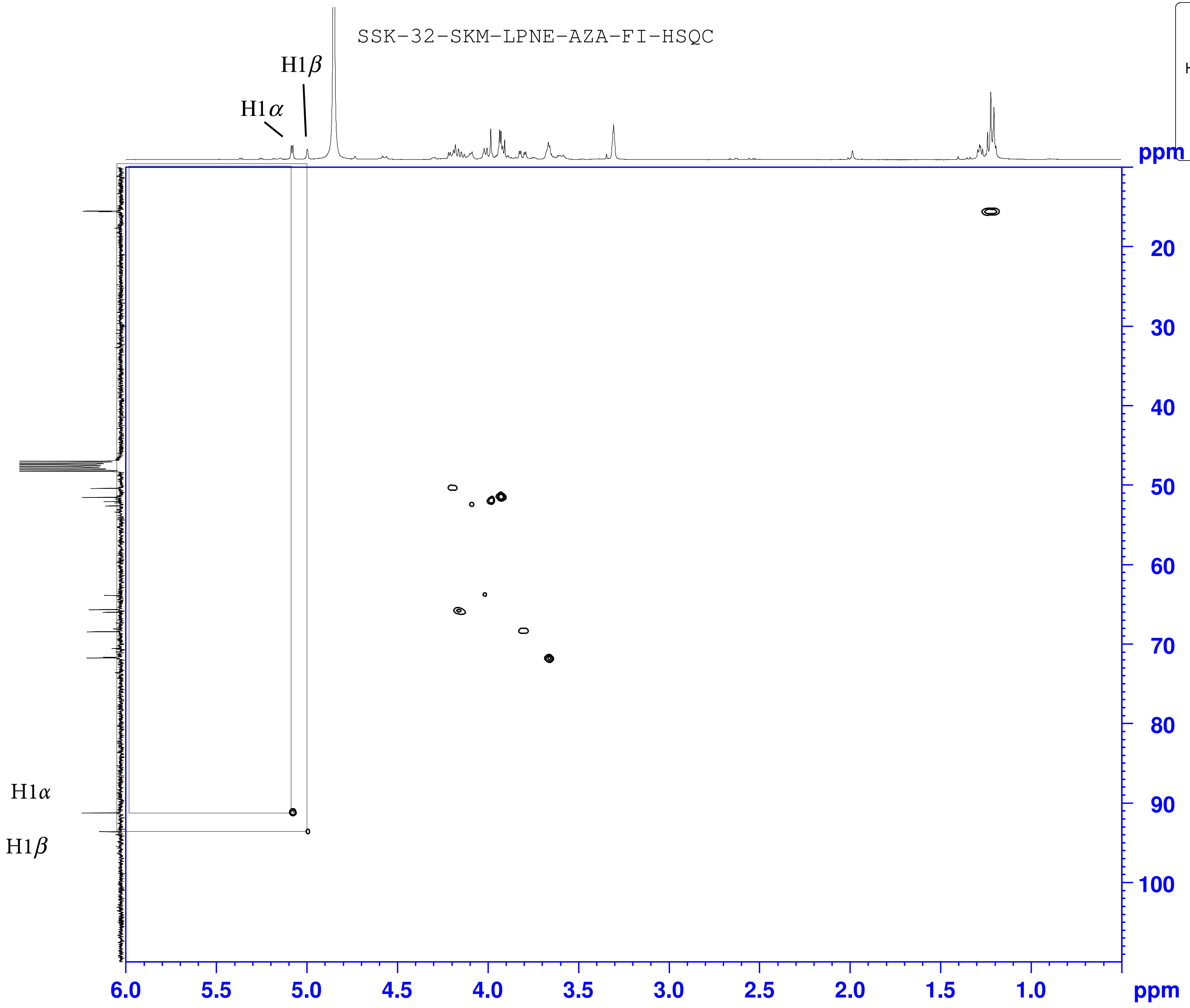

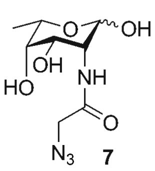

7

α:β=2:1

```
Current Data Parameters
NAME      SSK-32-SKM-LPNE-AZA-FI-HSQC
EXPNO                  35
PROCNO                  1

F2 - Acquisition Parameters
Date_            20250520
Time                22.08 h
INSTRUM             spect
PROBHD    Z104450_0346 (
PULPROG          hsqcetgp
TD                   2048
SOLVENT              MeOD
NS                      2
DS                      0
SWH              8802.817 Hz
FIDRES           8.596501 Hz
AQ              0.1163264 sec
RG                   2050
DW                 56.800 usec
DE                   6.50 usec
TE                  168.9 K
CNST2         145.0000000
D0             0.00000300 sec
D1             1.00000000 sec
D4             0.00172414 sec
D11            0.03000000 sec
D16            0.00020000 sec
IN0            0.00001800 sec
TDav                    1
ZGOPTNS
SFO1          400.1324708 MHz
NUC1                   1H
P1                  15.00 usec
P2                  30.00 usec
PLW1           9.69999981 W
SFO2          100.6248425 MHz
NUC2                  13C
CPDPRG[2             garp
P3                  10.00 usec
P4                  20.00 usec
PCPD2               80.00 usec
PLW2          47.00000000 W
PLW12          0.73438001 W
GPNAM[1]         SINE.100
GPZ1                80.00 %
GPNAM[2]         SINE.100
GPZ2                20.10 %
P16               1000.00 usec

F1 - Acquisition parameters
TD                    400
SFO1             100.6248 MHz
FIDRES         138.888885 Hz
SW                276.053 ppm
FnMODE       Echo-Antiecho

F2 - Processing parameters
SI                   2048
SF            400.1300081 MHz
WDW                 QSINE
SSB                     2
LB                      0 Hz
GB                      0
PC                   1.40

F1 - Processing parameters
SI                   1024
MC2          echo-antiecho
SF            100.6127690 MHz
WDW                 QSINE
SSB                     2
LB                      0 Hz
GB                      0
```

SSK-19-AG-RHAALL-1H

Current Data Parameters
NAME SSK-19-AG-RHAALL-1H
EXPNO 1
PROCNO 1

F2 - Acquisition Parameters
Date_ 20220421
Time 14.35 h
INSTRUM Avance
PROBHD Z824601_0063 (
PULPROG zg30
TD 51724
SOLVENT CDCl3
NS 14
DS 0
SWH 8620.689 Hz
FIDRES 0.333334 Hz
AQ 2.9999919 sec
RG 101
DW 58.000 usec
DE 11.94 usec
TE 297.0 K
D1 1.00000000 sec
TD0 1
SFO1 400.3024719 MHz
NUC1 1H
P0 5.00 usec
P1 15.00 usec
PLW1 9.20670033 W

F2 - Processing parameters
SI 65536
SF 400.3016108 MHz
WDW EM
SSB 0
LB 0.30 Hz
GB 0
PC 1.00

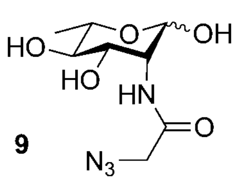

9

α:β=5:1

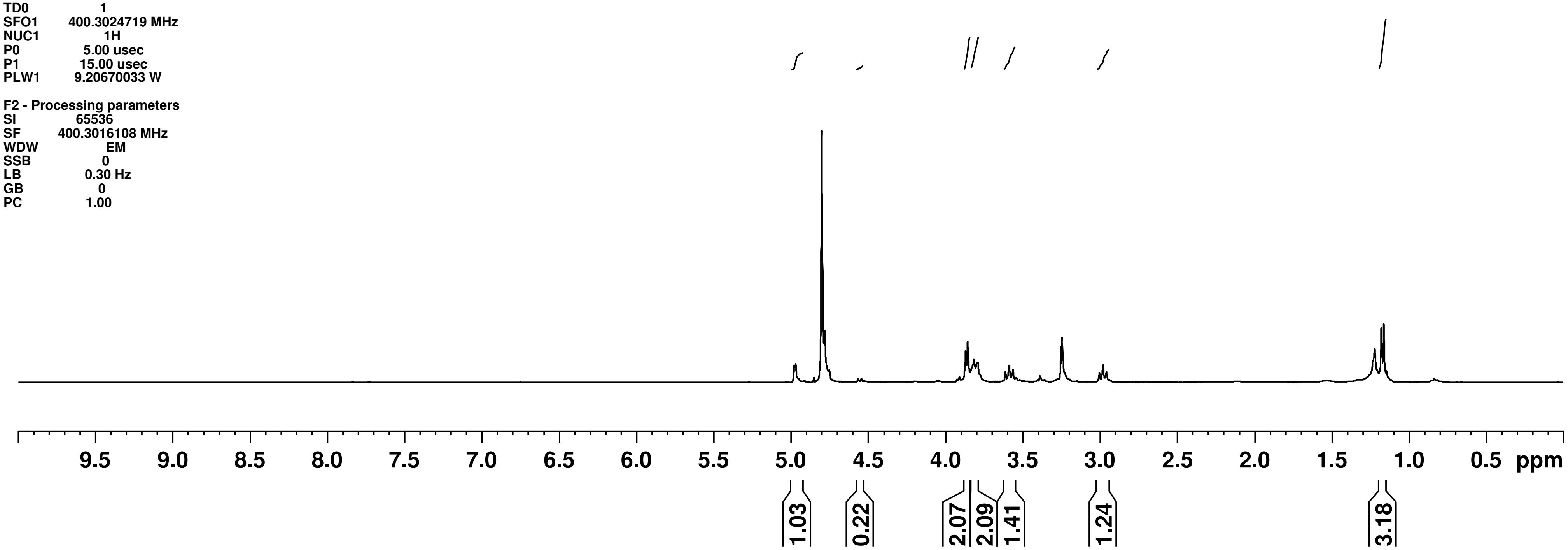

SSK-19-AG-RHALL-13C

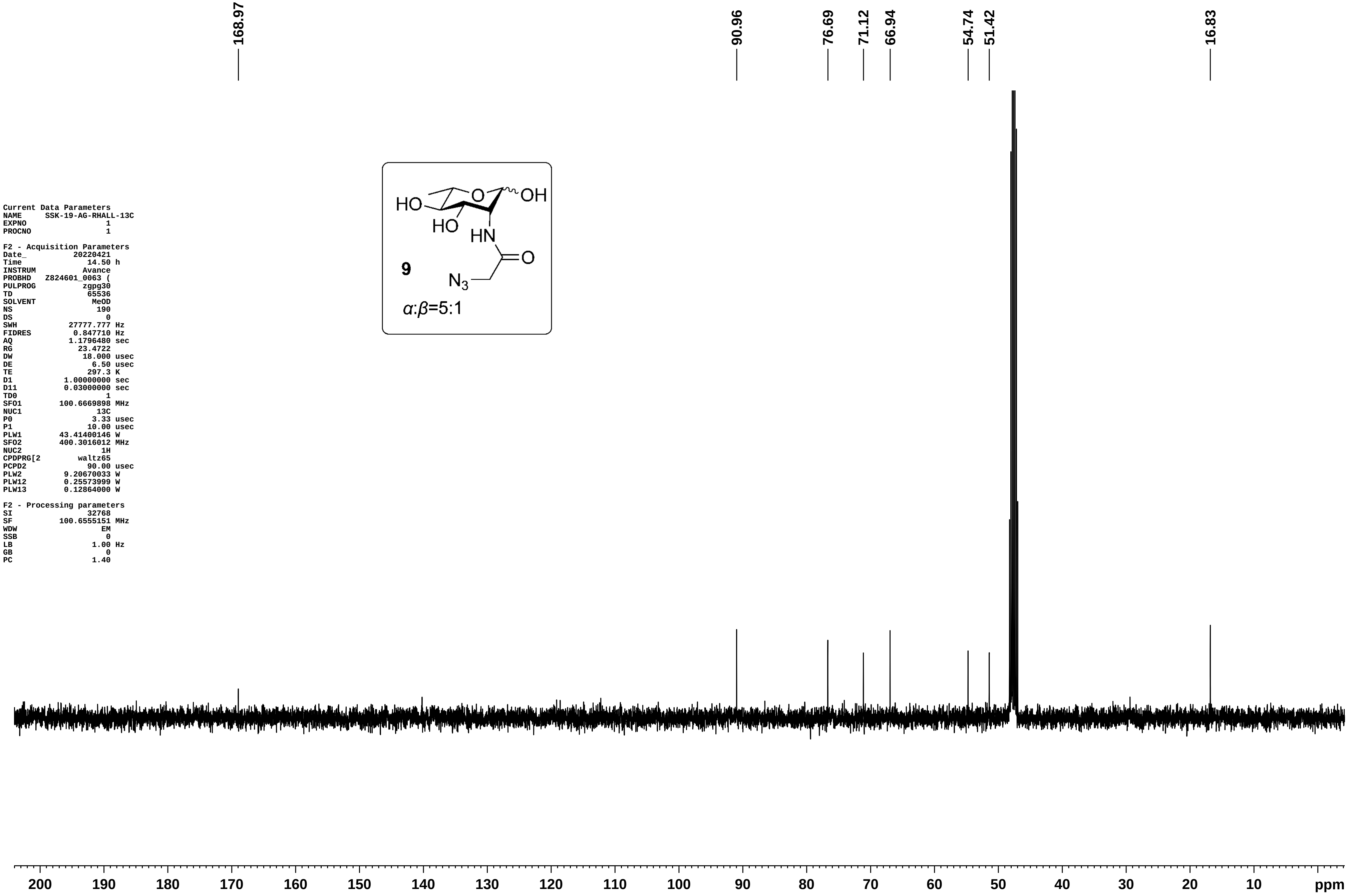

9

$\alpha:\beta$=5:1

SSK-19-AG-RHALL-DEPT

```
Current Data Parameters
NAME     SSK-19-AG-RHALL-DEPT
EXPNO                 1
PROCNO                1

F2 - Acquisition Parameters
Date_          20220421
Time              14.52 h
INSTRUM          Avance
PROBHD   Z824601_0063 (
PULPROG       deptsp135
TD                65536
SOLVENT            MeOD
NS                   58
DS                    0
SWH           27777.777 Hz
FIDRES         0.847710 Hz
AQ            1.1796480 sec
RG                  101
DW               18.000 usec
DE                 6.50 usec
TE                297.9 K
CNST2       145.0000000
D1           1.00000000 sec
D2           0.00344828 sec
D12          0.00002000 sec
TD0                   1
SFO1        100.6669898 MHz
NUC1                13C
P1                10.00 usec
P13             2000.00 usec
PLW0                  0 W
PLW1        43.41400146 W
SPNAM[5]    Crp60comp.4
SPOAL5            0.500
SPOFFS5               0 Hz
SPW5         6.63320017 W
SFO2        400.3016012 MHz
NUC2                 1H
CPDPRG[2        waltz65
P3                15.00 usec
P4                30.00 usec
PCPD2             90.00 usec
PLW2         9.20670033 W
PLW12        0.25573999 W

F2 - Processing parameters
SI                32768
SF          100.6555151 MHz
WDW                  EM
SSB                   0
LB                 1.00 Hz
GB                    0
PC                 1.40
```

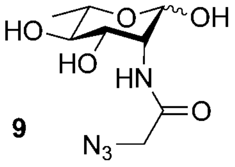

9

α:β=5:1

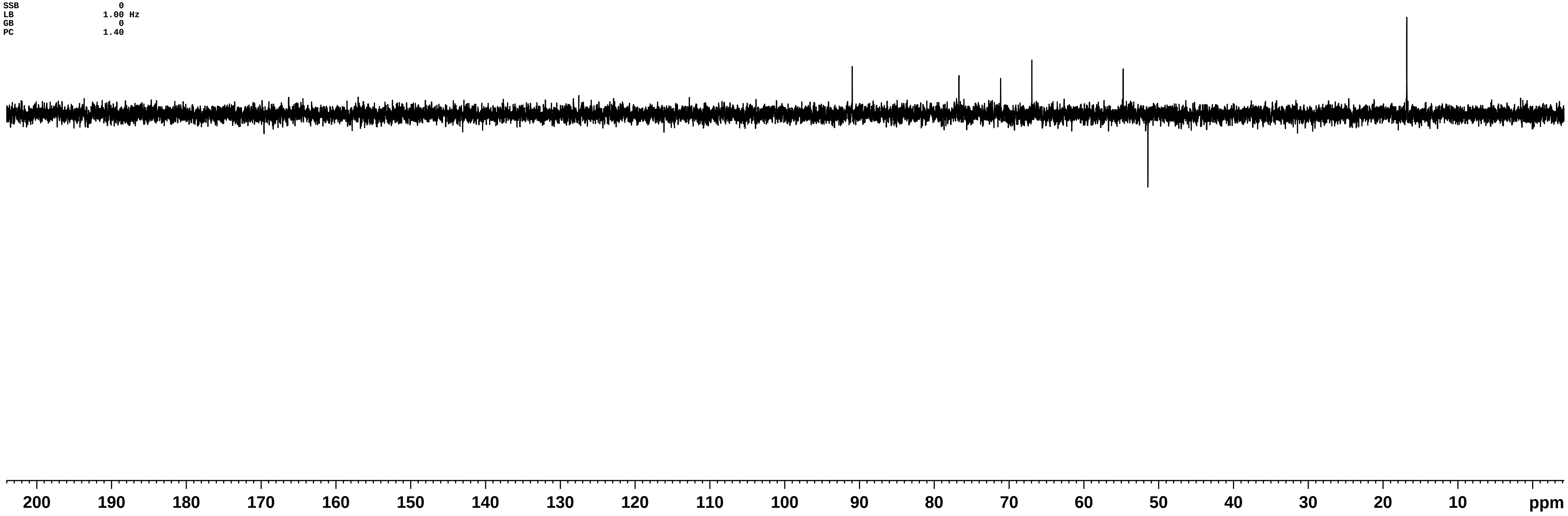

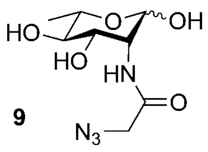

9

α:β=5:1

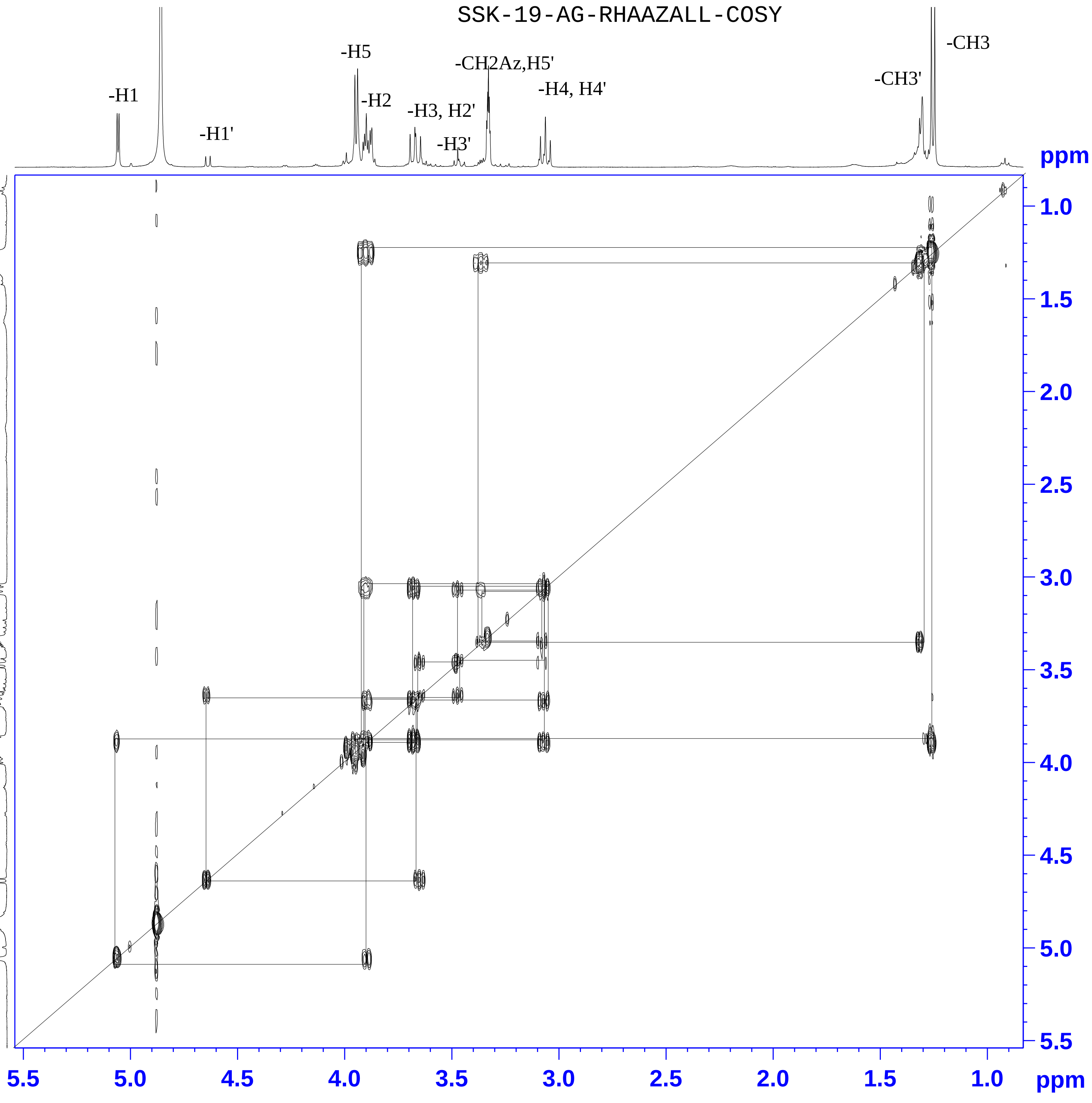


```
Current Data Parameters
NAME      SSK-19-AG-RHAAZALL-COSY
EXPNO                    2
PROCNO                   1

F2 - Acquisition Parameters
Date_             20220424
Time                 19.46
INSTRUM              spect
PROBHD    5 mm PABBO BB/
PULPROG          cosygpppqf
TD                    2048
SOLVENT               MeOD
NS                       6
DS                       0
SWH               2354.049 Hz
FIDRES            1.149438 Hz
AQ               0.4349952 sec
RG                  197.27
DW                 212.400 usec
DE                    6.50 usec
TE                   298.2 K
D0              0.00000300 sec
D1              1.00000000 sec
D11             0.03000000 sec
D12             0.00002000 sec
D13             0.00000400 sec
D16             0.00020000 sec
IN0             0.00042480 sec

======== CHANNEL f1 ========
SFO1           500.1315935 MHz
NUC1                    1H
P0                   13.35 usec
P1                   13.35 usec
P17                5000.00 usec
PLW1           16.00000000 W
PLW10           3.16840005 W

====== GRADIENT CHANNEL =====
GPNAM[1]        SMSQ10.100
GPZ1                 10.00 %
P16                1000.00 usec

F1 - Acquisition parameters
TD                      76
SFO1              500.1316 MHz
FIDRES           61.948658 Hz
SW                   4.707 ppm
FnMODE                  QF

F2 - Processing parameters
SI                    1024
SF             500.1300000 MHz
WDW                  QSINE
SSB                      0
LB                       0 Hz
GB                       0
PC                    1.40

F1 - Processing parameters
SI                    1024
MC2                     QF
SF             500.1300000 MHz
WDW                  QSINE
SSB                      0
LB                       0 Hz
GB                       0
```

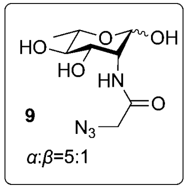

SSK-19-AG-RHAAZALL-HSQC

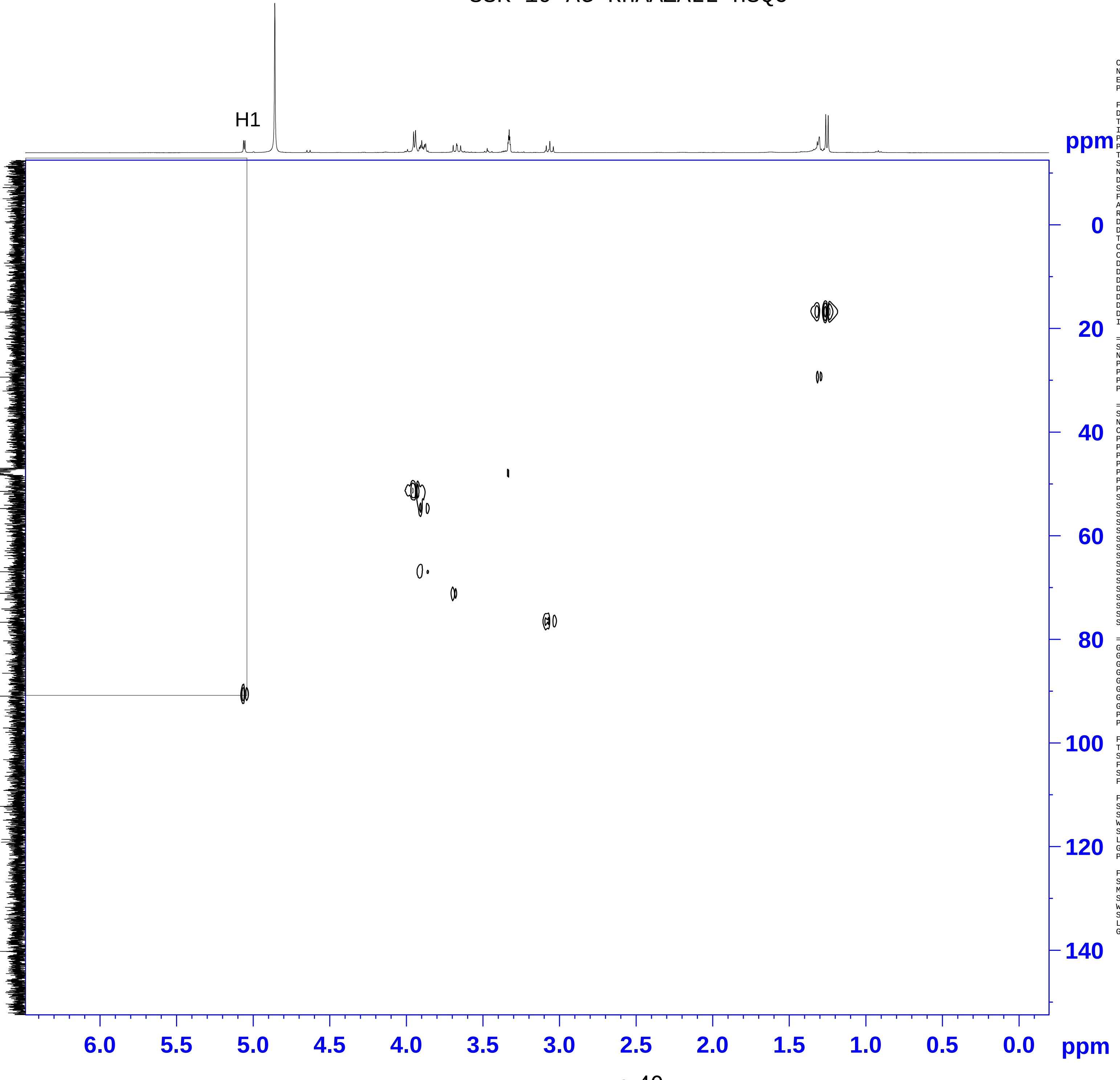


```
Current Data Parameters
NAME      SSK-19-AG-RHAAZALL-HSQC
EXPNO                    2
PROCNO                   1

F2 - Acquisition Parameters
Date_             20220424
Time                 19.58
INSTRUM              spect
PROBHD   5 mm PABBO BB/
PULPROG  hsqcedetgpsisp2.2
TD                    1024
SOLVENT               MeOD
NS                       8
DS                       0
SWH               3342.246 Hz
FIDRES            3.263912 Hz
AQ               0.1531904 sec
RG                  197.27
DW                 149.600 usec
DE                    6.50 usec
TE                   298.3 K
CNST2          145.0000000
CNST17          -0.5000000
D0              0.00000300 sec
D1              1.00000000 sec
D4              0.00172414 sec
D11             0.03000000 sec
D16             0.00020000 sec
D21             0.00360000 sec
D24             0.00089000 sec
IN0             0.00002410 sec

======== CHANNEL f1 ========
SFO1          500.1315733 MHz
NUC1                    1H
P1                   13.35 usec
P2                   26.70 usec
P28                2000.00 usec
PLW1           16.00000000 W

======== CHANNEL f2 ========
SFO2          125.7665916 MHz
NUC2                   13C
CPDPRG[2 bi_p5m4sp_4sp.2
P3                    8.90 usec
P14                 500.00 usec
P24                2000.00 usec
P63                1500.00 usec
PLW0                     0 W
PLW2          103.00000000 W
PLW12           1.66499996 W
SPNAM[3] Crp60,0.5,20.1
SPOAL3               0.500
SPOFFS3                  0 Hz
SPW3           12.46500015 W
SPNAM[7]      Crp60comp.4
SPOAL7               0.500
SPOFFS7                  0 Hz
SPW7           12.46500015 W
SPNAM[14 Crp32,1.5,20.2
SPOAL14              0.500
SPOFFS14                 0 Hz
SPW14           5.31860018 W
SPNAM[31 Crp32,1.5,20.2
SPOAL31              0.500
SPOFFS31                 0 Hz
SPW31           1.32969999 W

====== GRADIENT CHANNEL =====
GPNAM[1]      SMSQ10.100
GPNAM[2]      SMSQ10.100
GPNAM[3]      SMSQ10.100
GPNAM[4]      SMSQ10.100
GPZ1                 80.00 %
GPZ2                 20.10 %
GPZ3                 11.00 %
GPZ4                 -5.00 %
P16                1000.00 usec
P19                 600.00 usec

F1 - Acquisition parameters
TD                      58
SFO1              125.7666 MHz
FIDRES          715.409912 Hz
SW                 164.963 ppm
FnMODE     Echo-Antiecho

F2 - Processing parameters
SI                    1024
SF             500.1300000 MHz
WDW                  QSINE
SSB                      2
LB                       0 Hz
GB                       0
PC                    1.40

F1 - Processing parameters
SI                    1024
MC2          echo-antiecho
SF             125.7577890 MHz
WDW                  QSINE
SSB                      2
LB                       0 Hz
GB                       0
```

SSK-23-AP-FUC-NBD-1H

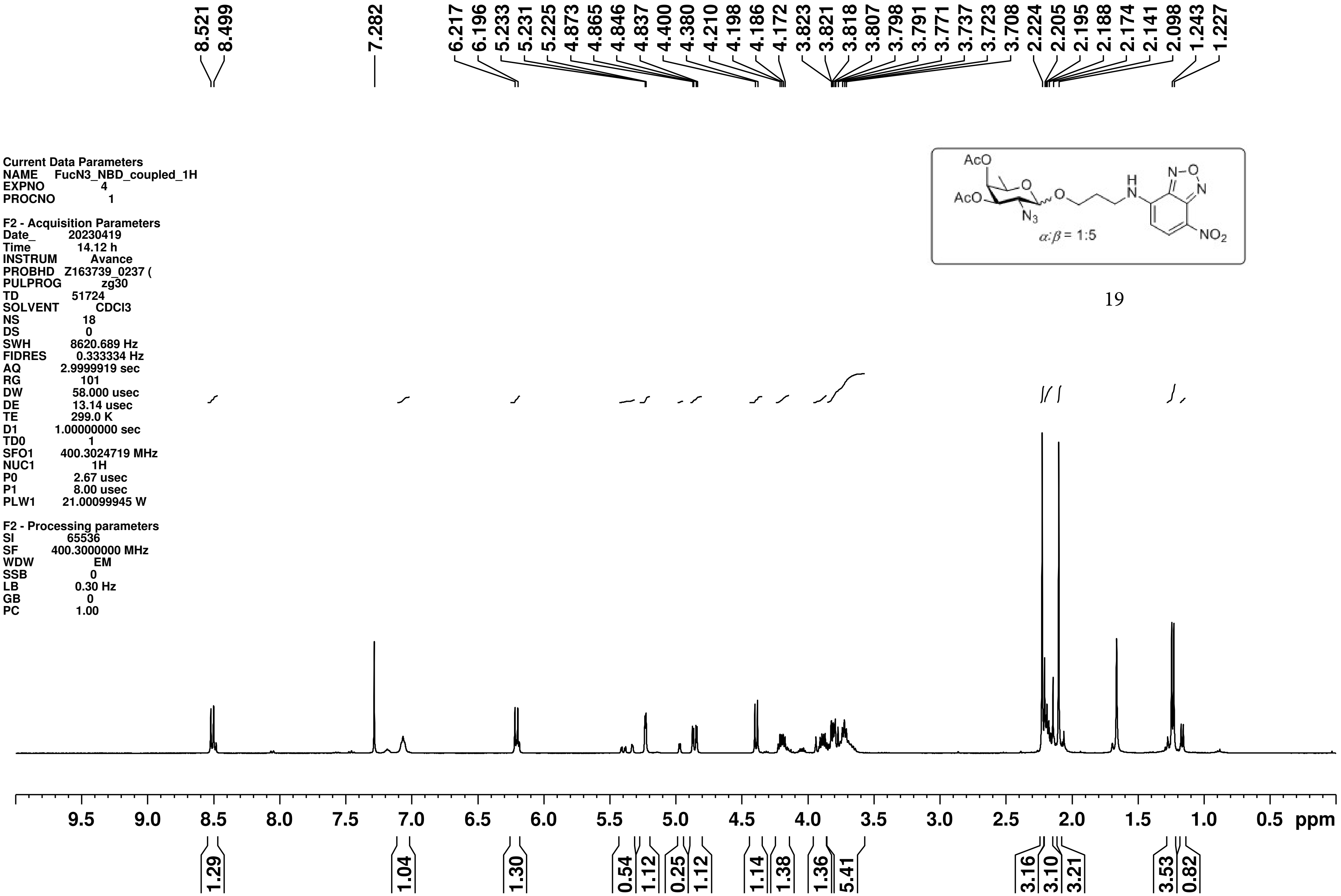

19

SSK-23-AP-866(P)-13C

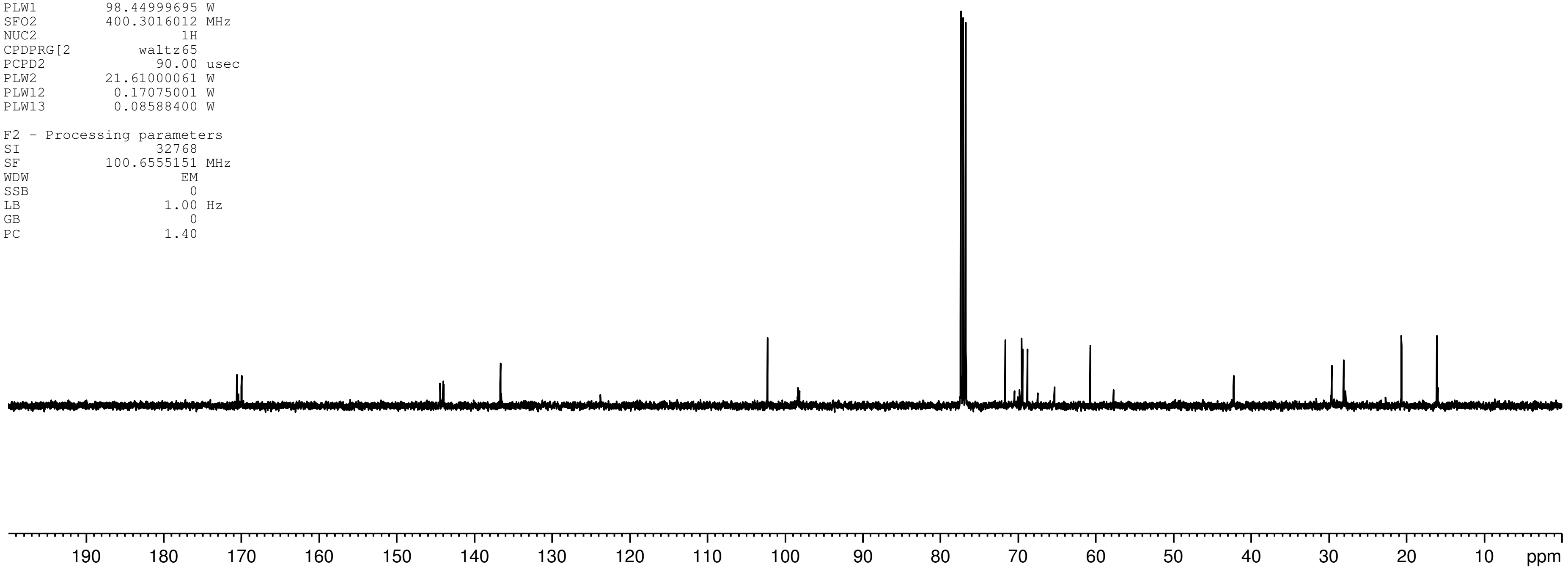


```
Current Data Parameters
NAME     FucN3_NBD_coupled-13C
EXPNO                  3
PROCNO                 1

F2 - Acquisition Parameters
Date_          20210618
Time              18.27 h
INSTRUM           Avance
PROBHD   Z163739_0237 (
PULPROG          zgpg30
TD                65536
SOLVENT           CDCl3
NS                  300
DS                    0
SWH           27777.777 Hz
FIDRES         0.847710 Hz
AQ            1.1796480 sec
RG                  101
DW               18.000 usec
DE                 6.50 usec
TE                300.4 K
D1           1.00000000 sec
D11          0.03000000 sec
TD0                   1
SFO1        100.6669898 MHz
NUC1                13C
P0                 2.67 usec
P1                 8.00 usec
PLW1        98.44999695 W
SFO2        400.3016012 MHz
NUC2                 1H
CPDPRG[2        waltz65
PCPD2             90.00 usec
PLW2        21.61000061 W
PLW12        0.17075001 W
PLW13        0.08588400 W

F2 - Processing parameters
SI                32768
SF          100.6555151 MHz
WDW                  EM
SSB                   0
LB                 1.00 Hz
GB                    0
PC                 1.40
```

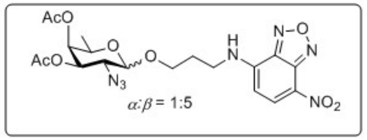

19

ssk-23-ap-866-dept

```
Current Data Parameters
NAME     ssk-23-ap-866-dept
EXPNO                     5
PROCNO                    1

F2 - Acquisition Parameters
Date_             20210614
Time                 19.38
INSTRUM              spect
PROBHD   5 mm PABBO BB/
PULPROG        deptsp135
TD                   65536
SOLVENT              CDCl3
NS                      48
DS                       4
SWH             20161.291 Hz
FIDRES           0.307637 Hz
AQ              1.6252928 sec
RG                  197.27
DW                  24.800 usec
DE                    6.50 usec
TE                   297.0 K
CNST2         145.0000000
D1             2.00000000 sec
D2             0.00344828 sec
D12            0.00002000 sec
TD0                      1

======== CHANNEL f1 ========
SFO1          125.7678486 MHz
NUC1                   13C
P1                    8.90 usec
P13                2000.00 usec
PLW0                     0 W
PLW1         103.00000000 W
SPNAM[5]      Crp60comp.4
SPOAL5               0.500
SPOFFS5                  0 Hz
SPW5           12.46500015 W

======== CHANNEL f2 ========
SFO2          500.1315995 MHz
NUC2                    1H
CPDPRG[2           waltz16
P3                   13.35 usec
P4                   26.70 usec
PCPD2                80.00 usec
PLW2          16.00000000 W
PLW12          0.44556001 W

F2 - Processing parameters
SI                   32768
SF            125.7577890 MHz
WDW                     EM
SSB                      0
LB                    1.00 Hz
GB                       0
PC                    1.40
```

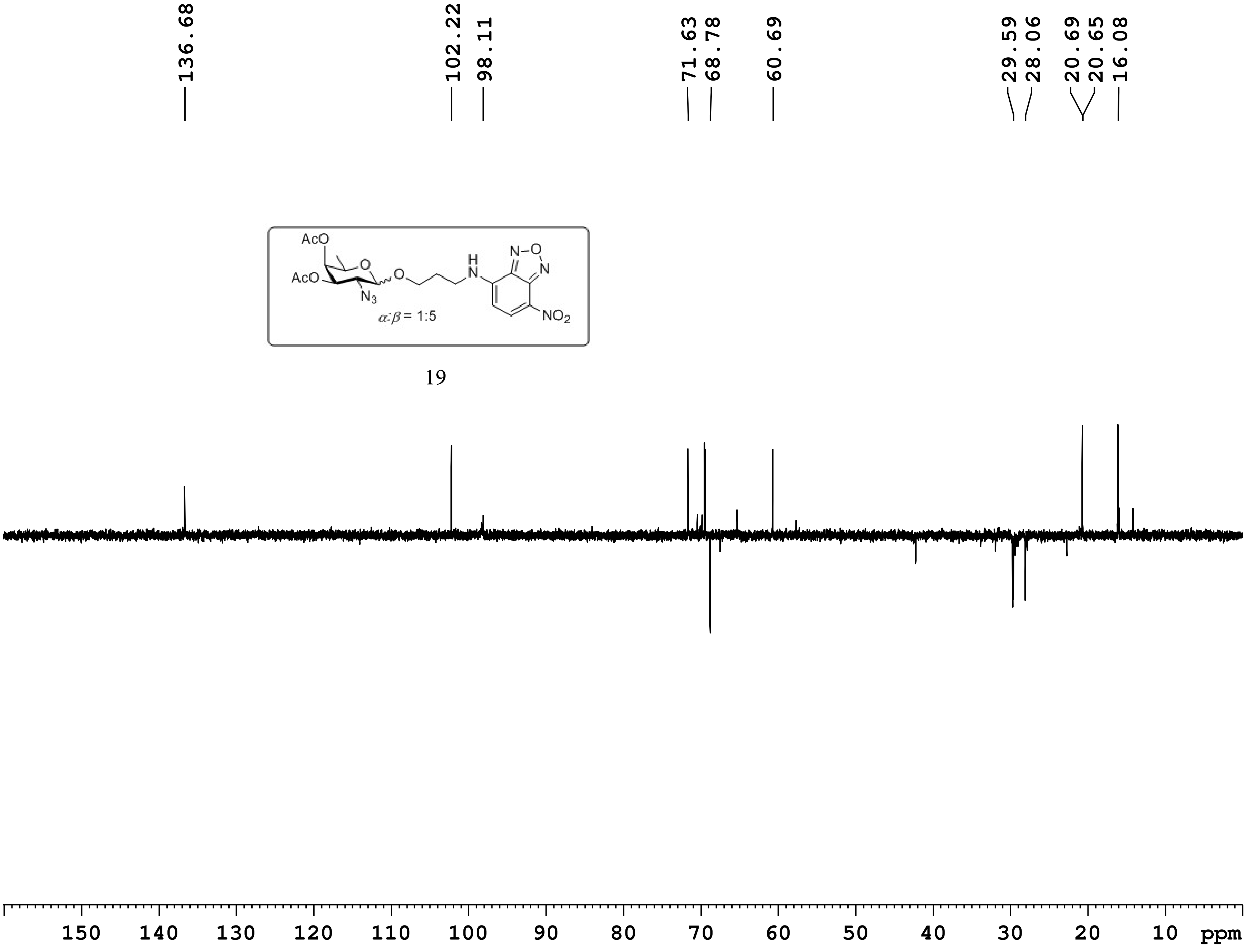

19

SSK-23-AP-FUC-NBD-F-4-1H

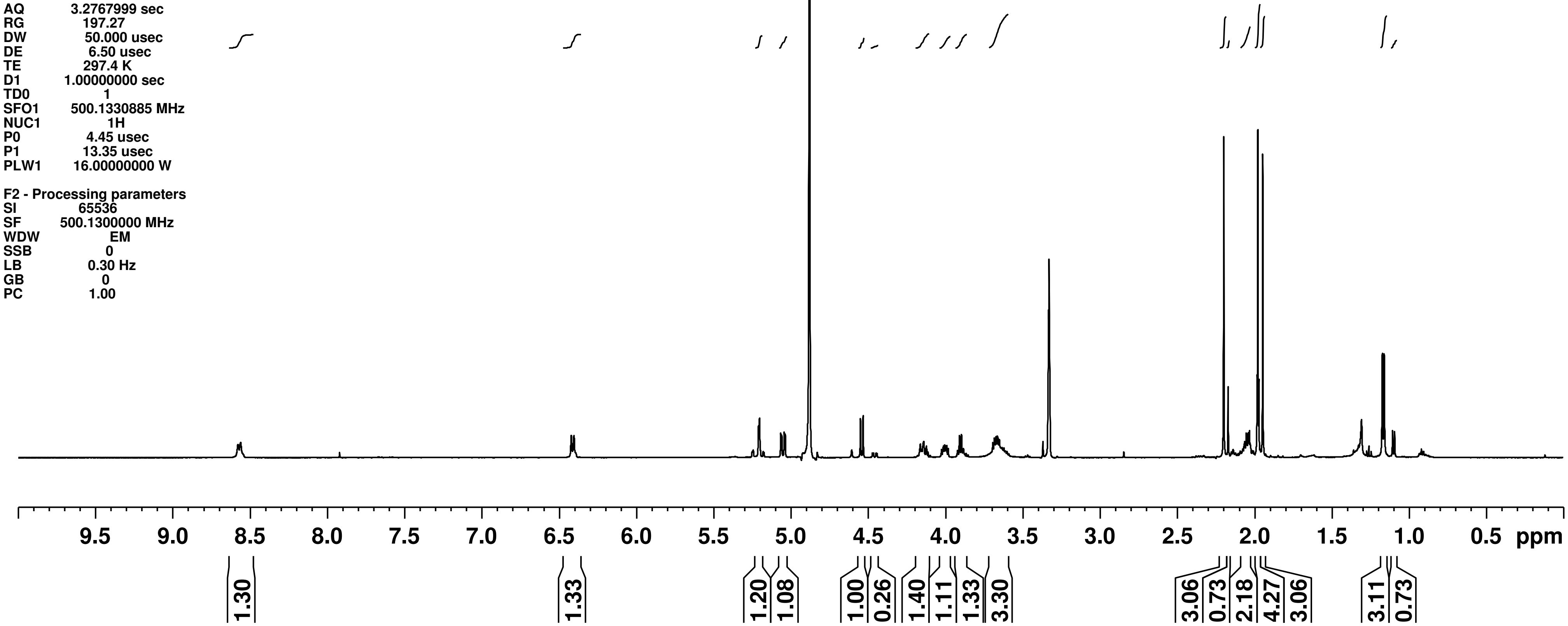

Current Data Parameters
NAME FucNHAc_NBD_Final_1H
EXPNO 1
PROCNO 1

F2 - Acquisition Parameters
Date_ 20230422
Time 15.58 h
INSTRUM spect
PROBHD Z119470_0087 (
PULPROG zg30
TD 65536
SOLVENT MeOD
NS 16
DS 0
SWH 10000.000 Hz
FIDRES 0.305176 Hz
AQ 3.2767999 sec
RG 197.27
DW 50.000 usec
DE 6.50 usec
TE 297.4 K
D1 1.00000000 sec
TD0 1
SFO1 500.1330885 MHz
NUC1 1H
P0 4.45 usec
P1 13.35 usec
PLW1 16.00000000 W

F2 - Processing parameters
SI 65536
SF 500.1300000 MHz
WDW EM
SSB 0
LB 0.30 Hz
GB 0
PC 1.00

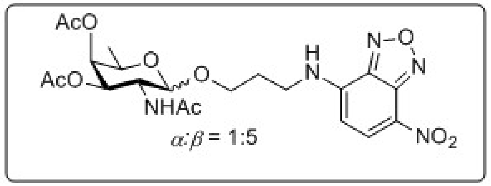

12

SSK-23-AP-FUC-NBD-F-4-13C

```
Current Data Parameters
NAME     FucNHAc_NBD_Final-13C
EXPNO                  2
PROCNO                 1

F2 - Acquisition Parameters
Date_           20230422
Time               17.01 h
INSTRUM            spect
PROBHD   Z119470_0087 (
PULPROG          zgpg30
TD                65536
SOLVENT            MeOD
NS                 1732
DS                    0
SWH           29761.904 Hz
FIDRES         0.908261 Hz
AQ            1.1010048 sec
RG               197.27
DW               16.800 usec
DE                 6.50 usec
TE                298.1 K
D1           1.00000000 sec
D11          0.03000000 sec
TD0                   1
SFO1        125.7703637 MHz
NUC1                13C
P0                 2.97 usec
P1                 8.90 usec
PLW1       103.00000000 W
SFO2        500.1320005 MHz
NUC2                 1H
CPDPRG[2        waltz16
PCPD2             80.00 usec
PLW2        16.00000000 W
PLW12        0.44556001 W
PLW13        0.22411001 W

F2 - Processing parameters
SI                32768
SF          125.7577890 MHz
WDW                  EM
SSB                   0
LB                 1.00 Hz
GB                    0
PC                 1.40
```

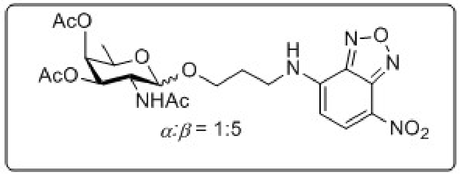

12

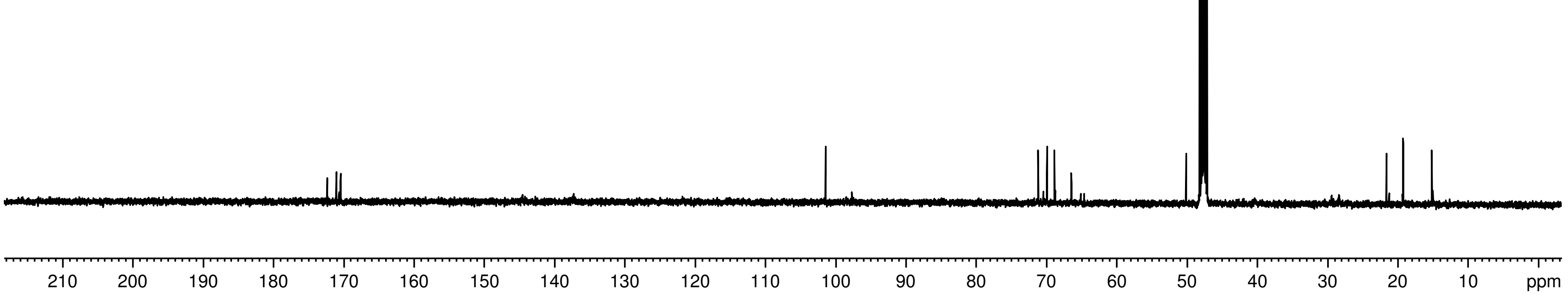

SSK-23-AP-876-HSQC

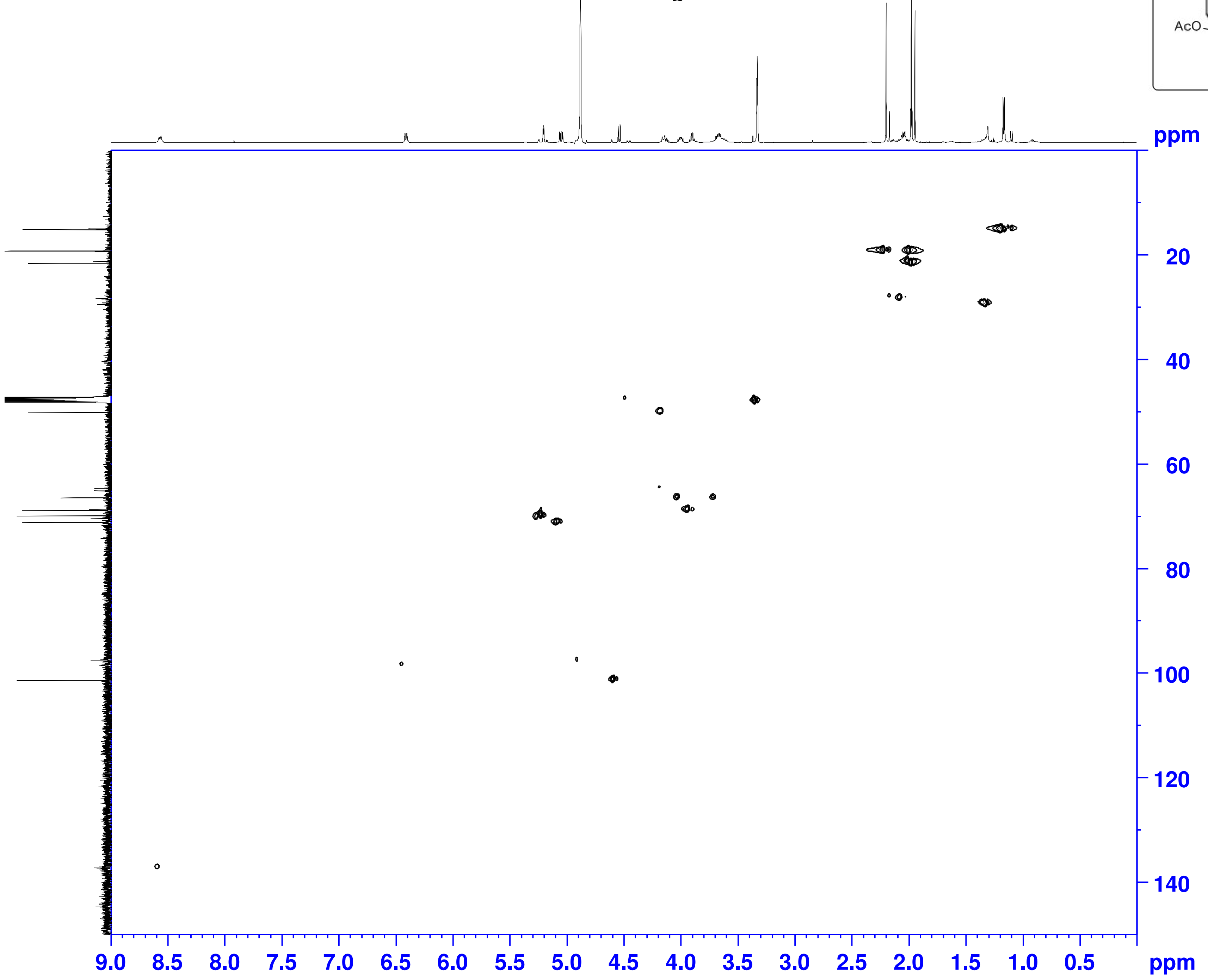

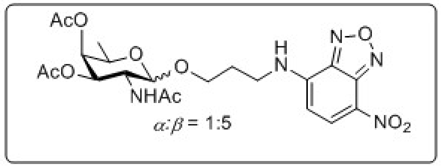

12

```
Current Data Parameters
NAME      FucNHAc_NBD_Final_HSQC
EXPNO                    9
PROCNO                   1

F2 - Acquisition Parameters
Date_             20210711
Time                 23.42
INSTRUM              spect
PROBHD   5 mm PABBO BB/
PULPROG  hsqcedetgpsisp2.2
TD                    1024
SOLVENT               MeOD
NS                       8
DS                       0
SWH               3924.647 Hz
FIDRES            3.832663 Hz
AQ               0.1304576 sec
RG                  197.27
DW                 127.400 usec
DE                    6.50 usec
TE                   297.1 K
CNST2          145.0000000
CNST17          -0.5000000
D0              0.00000300 sec
D1              1.00000000 sec
D4              0.00172414 sec
D11             0.03000000 sec
D16             0.00020000 sec
D21             0.00360000 sec
D24             0.00089000 sec
IN0             0.00002410 sec

======== CHANNEL f1 ========
SFO1           500.1324489 MHz
NUC1                    1H
P1                   13.35 usec
P2                   26.70 usec
P28                2000.00 usec
PLW1           16.00000000 W

======== CHANNEL f2 ========
SFO2           125.7665916 MHz
NUC2                   13C
CPDPRG[2 bi_p5m4sp_4sp.2
P3                    8.90 usec
P14                 500.00 usec
P24                2000.00 usec
P63                1500.00 usec
PLW0                     0 W
PLW2          103.00000000 W
PLW12           1.66499996 W
SPNAM[3] Crp60,0.5,20.1
SPOAL3               0.500
SPOFFS3                  0 Hz
SPW3           12.46500015 W
SPNAM[7]    Crp60comp.4
SPOAL7               0.500
SPOFFS7                  0 Hz
SPW7           12.46500015 W
SPNAM[14 Crp32,1.5,20.2
SPOAL14              0.500
SPOFFS14                 0 Hz
SPW14           5.31860018 W
SPNAM[31 Crp32,1.5,20.2
SPOAL31              0.500
SPOFFS31                 0 Hz
SPW31           1.32969999 W

====== GRADIENT CHANNEL =====
GPNAM[1]      SMSQ10.100
GPNAM[2]      SMSQ10.100
GPNAM[3]      SMSQ10.100
GPNAM[4]      SMSQ10.100
GPZ1                 80.00 %
GPZ2                 20.10 %
GPZ3                 11.00 %
GPZ4                 -5.00 %
P16                1000.00 usec
P19                 600.00 usec

F1 - Acquisition parameters
TD                     191
SFO1              125.7666 MHz
FIDRES          217.244904 Hz
SW                 164.963 ppm
FnMODE     Echo-Antiecho

F2 - Processing parameters
SI                    1024
SF             500.1299900 MHz
WDW                  QSINE
SSB                      2
LB                       0 Hz
GB                       0
PC                    1.40

F1 - Processing parameters
SI                    1024
MC2         echo-antiecho
SF             125.7577890 MHz
WDW                  QSINE
SSB                      2
LB                       0 Hz
GB                       0
```
